# Pentacyclic Triterpenoids Isolated from Celastraceae: A Focus in the ^13^C-NMR Data

**DOI:** 10.3390/molecules27030959

**Published:** 2022-01-31

**Authors:** Karen Caroline Camargo, Mariana Guerra de Aguilar, Acácio Raphael Aguiar Moraes, Raquel Goes de Castro, Daiane Szczerbowski, Elizabeth Luciana Marinho Miguel, Leila Renan Oliveira, Grasiely Faria Sousa, Diogo Montes Vidal, Lucienir Pains Duarte

**Affiliations:** Departamento de Química, Instituto de Ciências Exatas, Universidade Federal de Minas Gerais, Avenida Presidente Antônio Carlos, 6627, Pampulha, Belo Horizonte 31270-901, MG, Brazil; karen_camargo345@hotmail.com (K.C.C.); marianag.a9@gmail.com (M.G.d.A.); acacioramoraes@gmail.com (A.R.A.M.); raquelgooes@hotmail.com (R.G.d.C.); daianeszcz@gmail.com (D.S.); elizabethmmiguel@yahoo.com.br (E.L.M.M.); leila.renan97@hotmail.com (L.R.O.)

**Keywords:** Celastraceae, triterpenes, quinonemethide, ^13^C-NMR

## Abstract

The Celastraceae family comprises about 96 genera and more than 1.350 species, occurring mainly in tropical and subtropical regions of the world. The species of this family stand out as important plant sources of triterpenes, both in terms of abundance and structural diversity. Triterpenoids found in Celastraceae species display mainly lupane, ursane, oleanane, and friedelane skeletons, exhibiting a wide range of biological activities such as antiviral, antimicrobial, analgesic, anti-inflammatory, and cytotoxic against various tumor cell lines. This review aimed to document all triterpenes isolated from different botanical parts of species of the Celastraceae family covering 2001 to 2021. Furthermore, a compilation of their ^13^C-NMR data was carried out to help characterize compounds in future investigations. A total of 504 pentacyclic triterpenes were compiled and distinguished as 29 aromatic, 50 dimers, 103 friedelanes, 89 lupanes, 102 oleananes, 22 quinonemethides, 88 ursanes and 21 classified as others.

## 1. Introduction

The Celastraceae family comprises approximately 96 genera, reaching about 1350 species distributed in the tropical and subtropical regions of the world [1,2]. Species of this family stand out for producing compounds with several pharmacological activities, such as antitumor [3,4], anti-inflammatory [5], antimicrobial [6,7,8], antioxidant [9] antiviral [10], analgesic [5,11], antiulcerogenic [12], hepatoprotective [13], hypoglycemic [13,14], immunomodulatory [15], among others. Considering the chemical composition, species of the Celastraceae family are rich in pentacyclic triterpenes (PCTTs). PCTTs show a range of biological properties, characterizing these plants as research targets aiming to obtain new bioactive compounds or prototypes of new drugs [16,17,18,19,20,21,22].

PCTTs are structurally diverse compounds and are therefore classified according to their main skeletal structure. The main classes found in Celastraceae family possess friedelane, oleanane, lupane, ursane and quinonemethide skeletons. Quinonemethides are chemomarkers of this family are found exclusively in these species [13]. These PCTTs can occur as alcohols, ketones, carboxylic acids, lactones, aldehydes, epoxides, esters, or even glycosylated derivatives. Furthermore, these PCTTs can be sub-classified as *seco*, generally due to the opening of one of their rings, the most common being the ring ‘A’ opening between carbons 3 and 4, and sub-classified as *nor* when there is a lack of any of the methyl groups that constitute the basic skeleton.

This review aims to present the PCTTs reported for species of the Celastraceae family in the 21st century, exhibiting from which species they were isolated and contributing to the chemical characterization process of these compounds listing their ^13^C NMR data. The information about the PCTTs was obtained from SciFinder, Scopus, and Web of Science, using as key search terms: “Celastraceae and triterpenes”, “Celastraceae and compounds”, “Celastraceae and phytochemistry” and “Celastraceae and metabolites”. Articles with only ethnopharmacological information and data from *in vitro* and *in vivo* tests involving extracts or isolated substances were excluded. The period covering from January 2001 to September 2021 was considered since the group has already developed a free online database (in Portuguese) for the previous years [23]. This review reports a total of 504 pentacyclic triterpenoids, 29 aromatics (A), 50 dimers (D), 103 friedelanes (F), 89 lupanes (L), 102 oleananes (O), 22 quinonemethides (Q), 88 ursanes (U) and 21 classified as others. Table S1 (Supplementary Material) summarizes all these PCTTs, as well as the plant species and parts from which they were isolated. 

## 2. Pentacyclic Triterpenoids (PCTTs)

**Figure 1:**
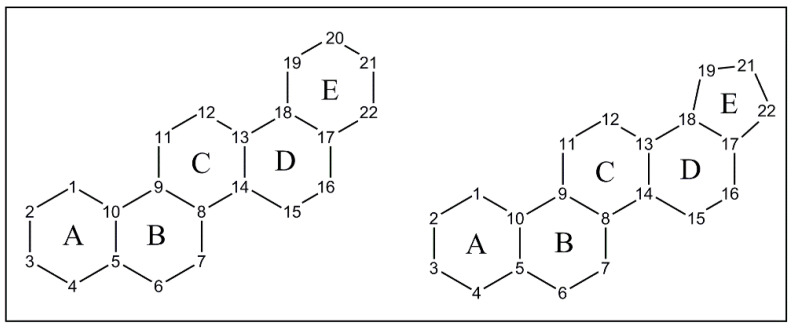
Core rings A, B, C, D, and E found in PCTTs.

PCTTs consist of 30 carbon atoms (six isoprene units) distributed over five fused rings (named A, B, C, D and E). This ring arrangement yields five six-membered rings or four six-membered rings fused to a 5-membered ring, numbered as shown in Figure 1 [24].

**Figure 2:**
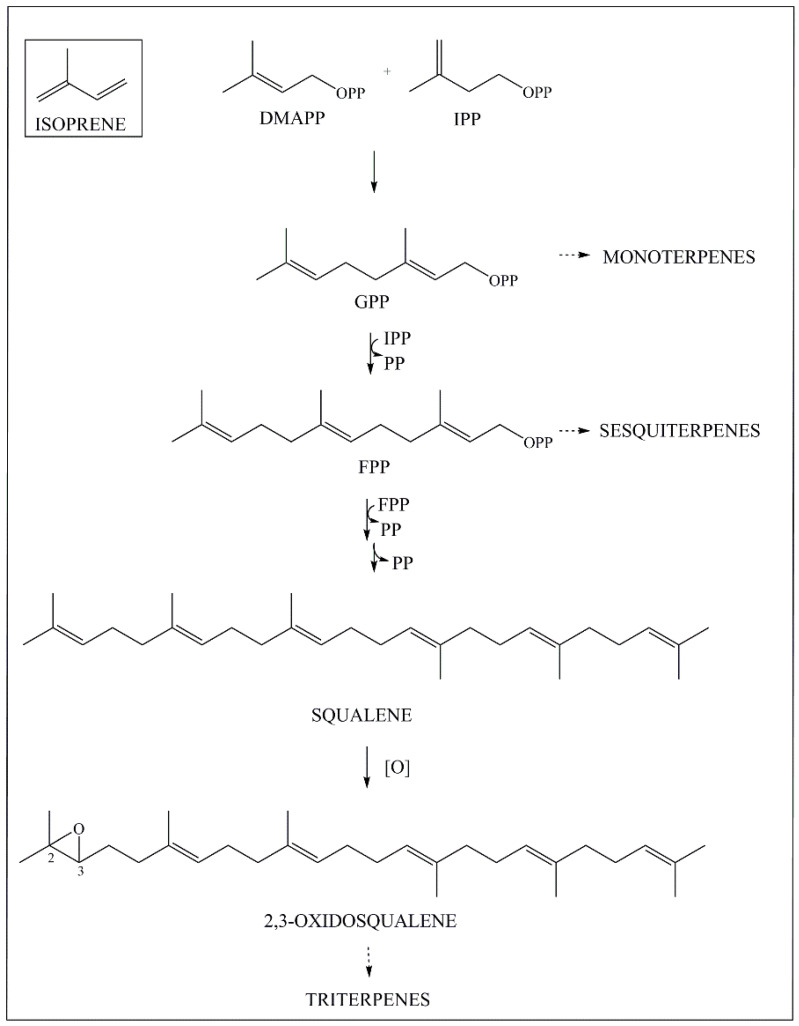
Simplified biosynthetic route of 2,3−oxidosqualene, the direct precursor of triterpenes, from isoprene. DMAPP: dimethylallyl diphosphate; IPP: isopentenyl diphosphate; GPP: geranyl diphosphate; FPP: farnesyl diphosphate; PP: diphosphate [26,28].

As terpenes, the biosynthesis of PCTTs starts by the coupling of active isoprene units. Initially, there is an electrophilic condensation of IPP (isopentenyl diphosphate), with DMAPP (dimethylallyl diphosphate), yielding the precursor of monoterpenes, geranyl diphosphate (GPP). The addition of IPP to GPP generates farnesyl diphosphate (FPP), which is the precursor of sesquiterpenes. Then a tail-tail condensation of two FPP molecules leads to squalene, after the release of a diphosphate unit and a 1,3-alkyl shift (Figure 2) [25].

**Figure 3:**
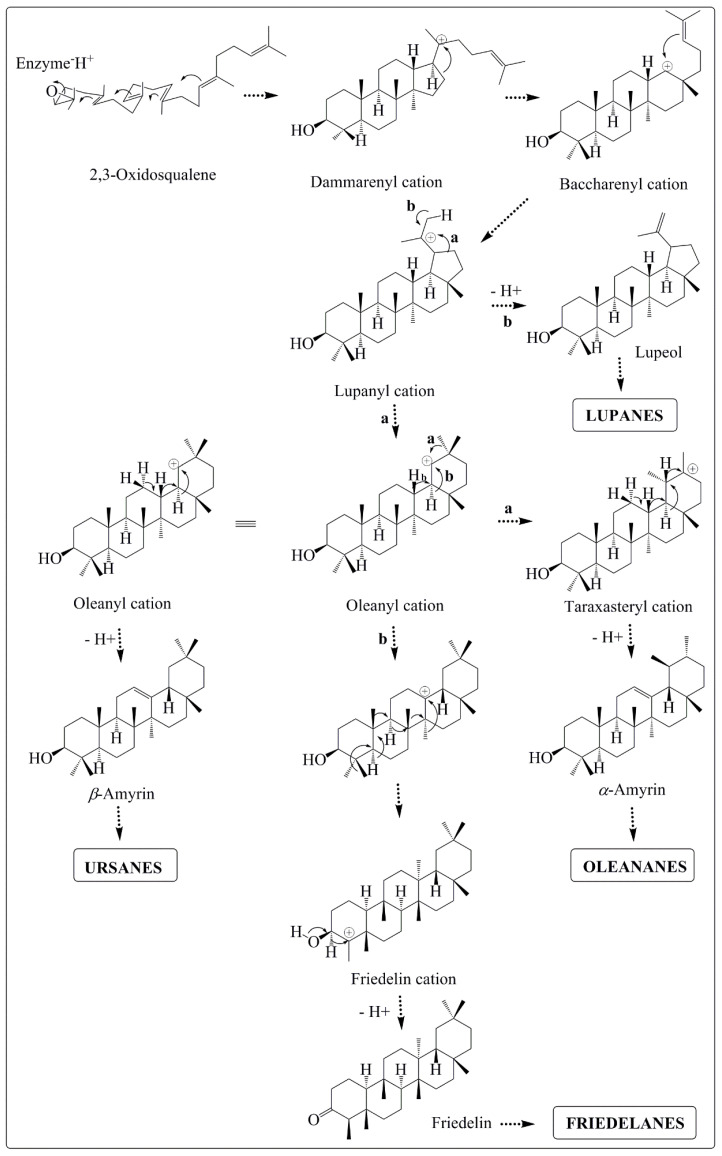
Simplified terpenoid biosynthetic route for the formation of the main pentacyclic triterpene skeletons isolated from Celastraceae species. “a” and “b” indicate two possible biosynthetic pathways [26,28].

The biosynthesis of PCTTs continues with the oxidation of squalene, catalyzed by squalene epoxidase, forming 2,3-oxidosqualene. This intermediary assumes the “chair-chair-chair-boat” conformation and after a sequence of cyclizations yields the dammarenyl cation, which then undergoes a rearrangement forming the baccharenyl cation. From the baccharenyl cation, the key step in PCTTs biosynthesis occurs, characterized by the formation of the lupanyl cation (Figure 3) [25,26]. Through a sequence of carbocation rearrangements (1,2-shifts), involving hydride, methyl, and ring-opening shifts, the lupanyl cation yields the different PCTTs skeletons, which then could oxidize, reduce, and isomerize, leading to the formation of the different currently known PCTTs [25,26].

The most powerful spectroscopic method in the structural elucidation of PCTTs is ^13^C Nuclear Magnetic Resonance (NMR). Comparison of experimental ^13^C NMR chemical shifts with literature data is a useful tool in identifying the basic skeleton of these compounds. Through this data, it is possible to make predictions about the influence of a functional group on the chemical displacement of carbons from its basic skeleton [27]. According to Mahato & Kundu [27], for example, the introduction of a hydroxyl group in the PCTT structure induces a deshielding of about 34–50 ppm of the *α* carbon, 2–10 ppm of the *β* carbons and 0–9 ppm of the *γ* carbons. The effect of the hydroxyl presence on the ^13^C NMR chemical shift of the *α*-carbon, is related to its configuration, and with the number of *γ*-gauche-type, and 1,3-diaxial-type interactions with the carbon atoms of the triterpene skeleton [27].

### 2.1. Friedelanes

**Figure 4:**
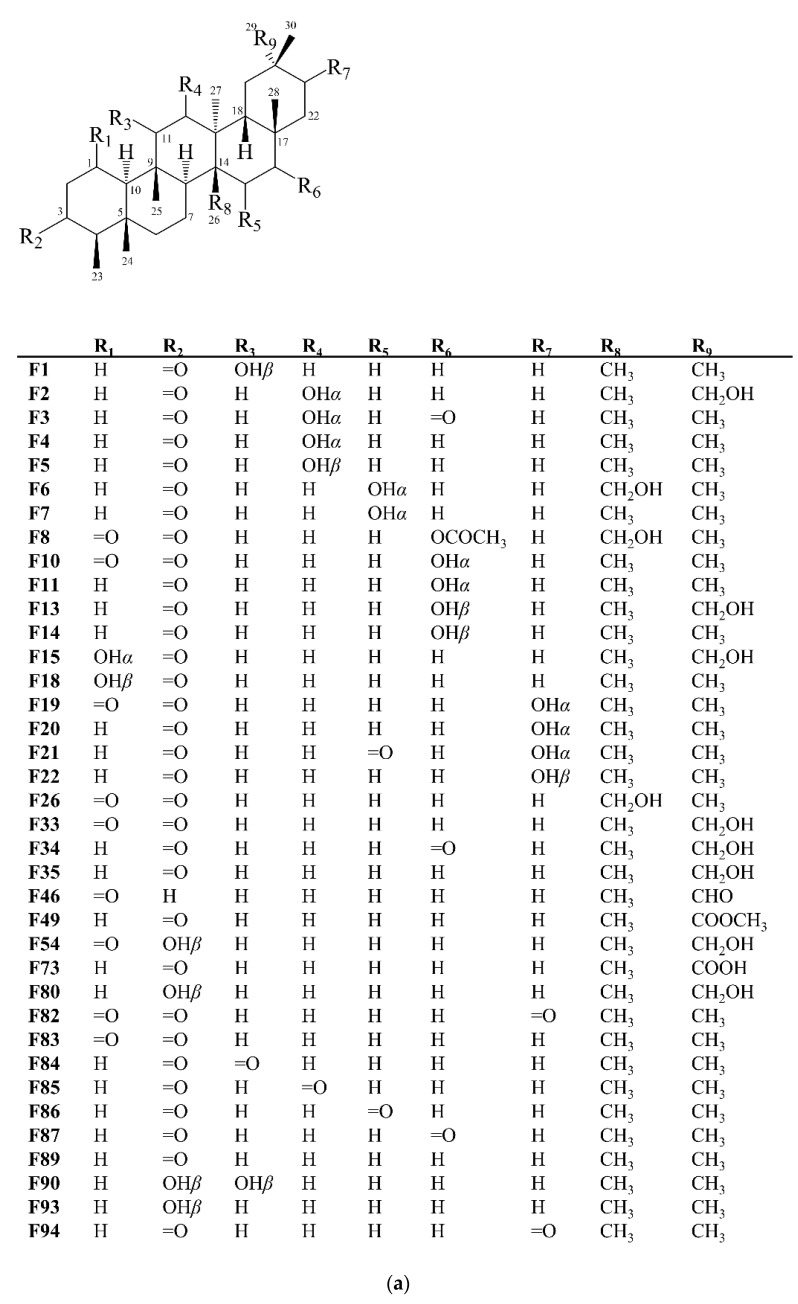

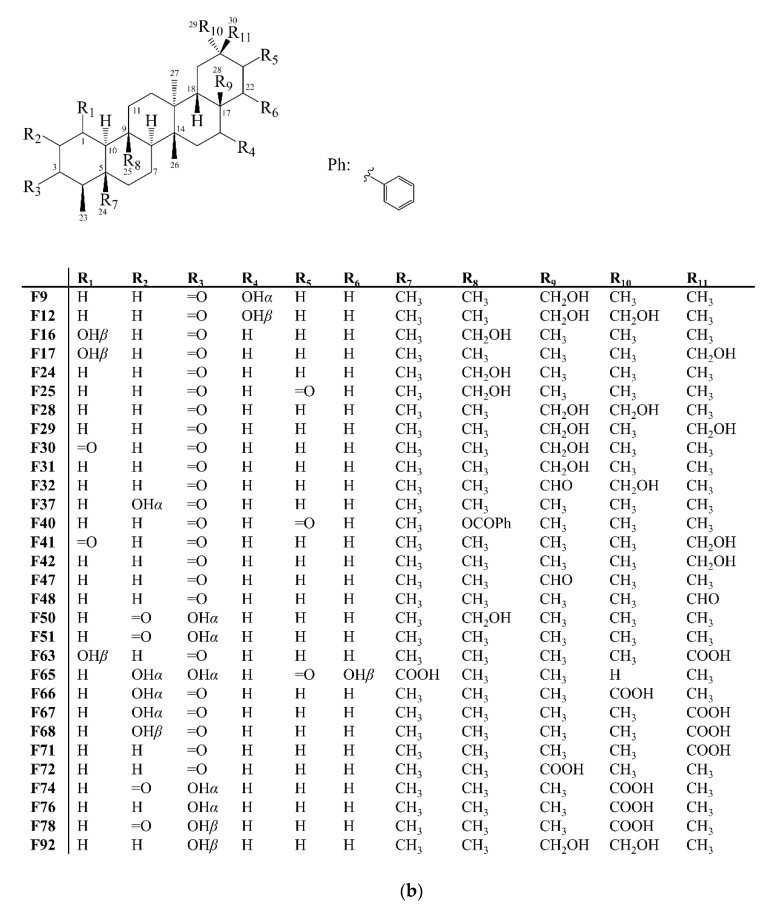

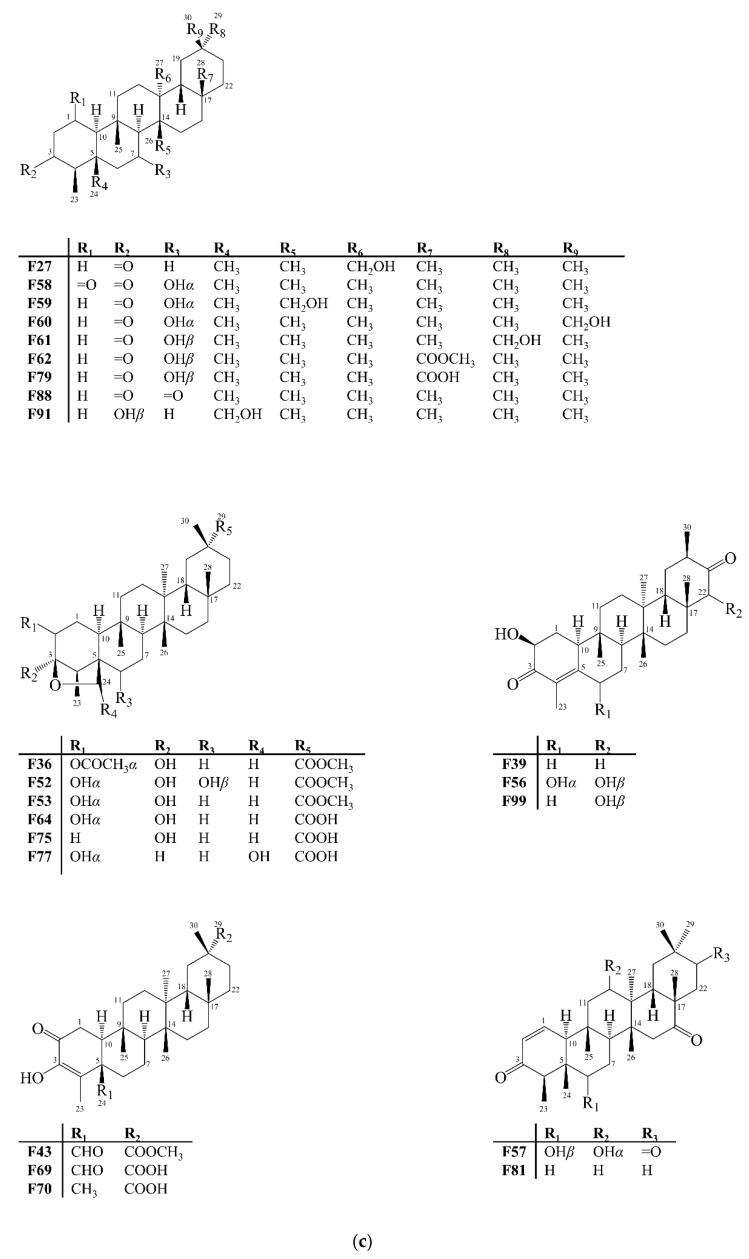

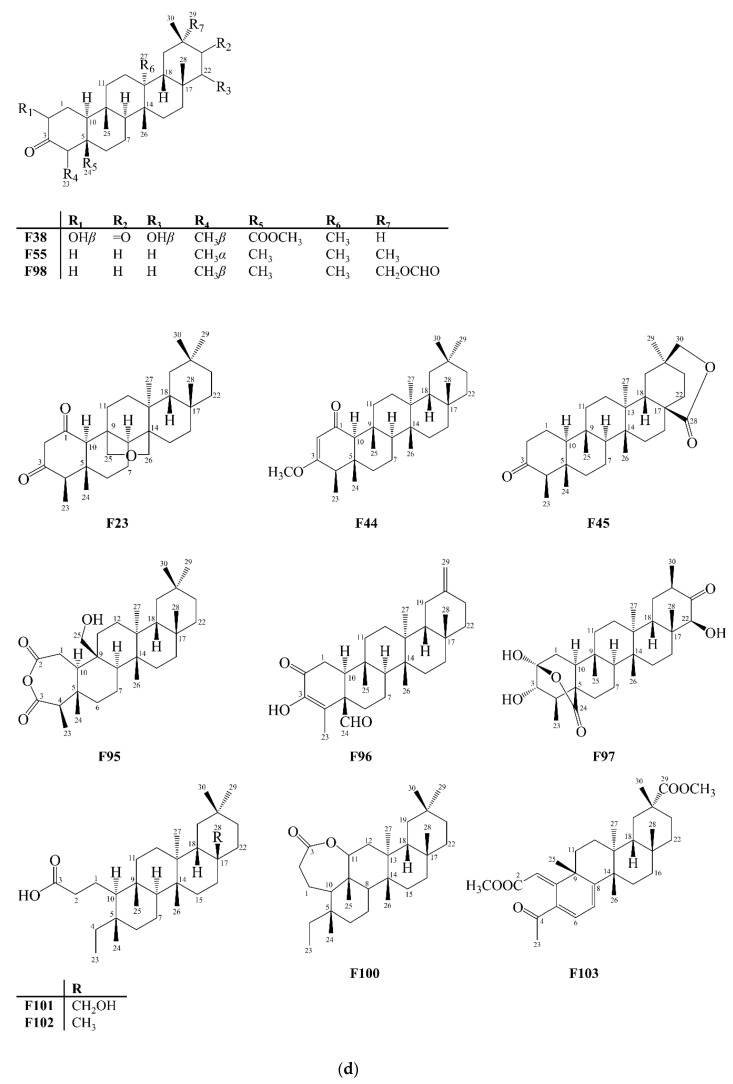
Structures of friedelane-type pentacyclic isolated from Celastraceae species (2001–2021). (**a**) Compounds **F1**−**F8**, **F10**, **F11**, **F13**−**F15**, **F18**−**F22**, **F26**, **F33**−**F35**, **F46**, **F49**, **F54**, **F73**, **F80**, **F82**−**F87**, **F89**, **F90**, **F93** and **F94**. (**b**) Compounds **F9**, **F12**, **F16**, **F17**, **F24**, **F25**, **F28**−**F32**, **F37**, **F40**−**F42**, **F47**, **F48**, **F50**, **F51**, **F63**, **F65**−**F68**, **F71**, **F72**, **F74**, **F76**, **F78** and **F92**. (**c**) Compounds **F27**, **F36**, **F39**, **F43**, **F52**, **F53**, **F56**, **F57**, **F58**−**F62**, **F64**, **F69**, **F70**, **F75**, **F77**, **F79**, **F81**, **F88**, **F91** and **F99**. (**d**) Compounds **F23**, **F38**, **F44**, **F45**, **F55**, **F95**−**F98**, **F100**−**F103**.

Compounds presenting a friedelane skeleton, together with the oleananes, are the most abundant PCTTs in the Celastraceae family, being found in the leaves, branches, roots and other parts of these plants [13]. These systems are formed by five six-membered rings fused. Rings A/B, B/C and C/D have *trans* configuration (H-10*α* and H-8*α*), while ring D/E is *cis* (H-18*β*). They have eight methyl groups; six attached to distinct carbons, at positions 4 (Me 23*β*), 5 (Me 24*β*), 9 (Me 25*β*), 13 (Me 27*α*), 14 (Me 26*β*) and 17 (Me 28*β*), and two geminal methyl groups at carbon 20 (Me 29*α* and 30*β*) [22,29]. In this work, 103 PCTTs of friedelan skeleton (**F**) are reported, compounds **F1**–**F103** (Figure 4).

An important observation in the ^13^C NMR data of 3-oxo friedelanes is the shielding of methyl group 23, which has a chemical shift value around *δ*_C_ 7.0 ppm. This occurs since this methyl is found in a cone region, generated by the *π* electrons of the carbonyl group at C-3, which promotes a region of shielding magnetic anisotropy [30].

### 2.2. Quinonemethides and Aromatics

**Figure 5:**
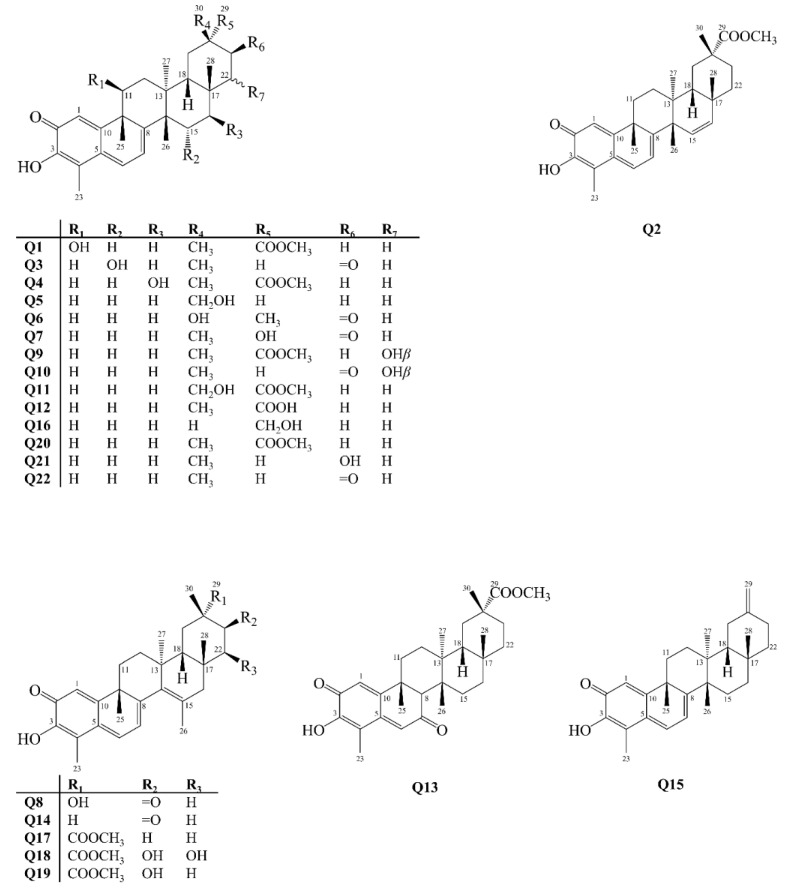
Structures of quinonemethide-type pentacyclic triterpenoids isolated from Celastraceae species (2001–2021).

**Figure 6:**
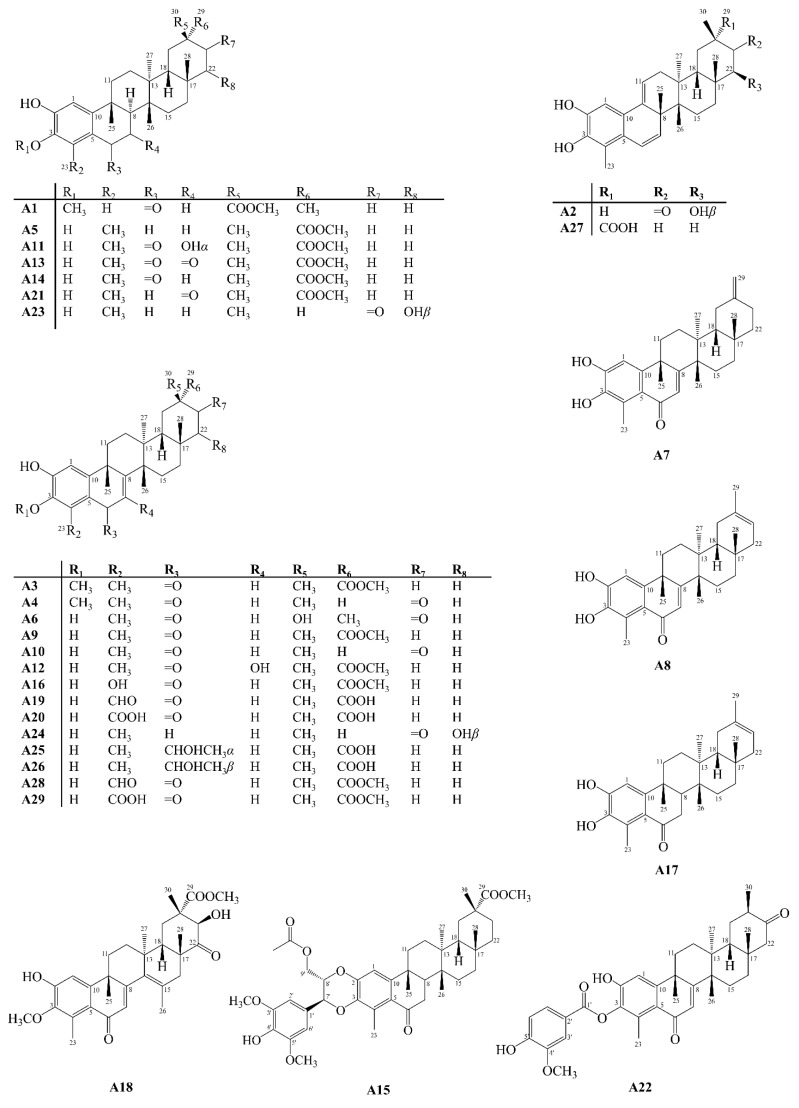
Structures of aromatic-type pentacyclic triterpenoids isolated from Celastraceae species (2001–2021).

Quinonemethides are compounds isolated exclusively in species of the Celastraceae family, and can also be found in the form of dimers or trimers [31]. Hypotheses about their origin assume that they are formed from friedelane derivatives, which are transported from the leaves to the roots, where they are converted into quinonemethides [32]. They are characterized as 24-*nor*-triterpenoids, due to the absence of methyl 24, and also they have functional oxygenated groups attached to carbons 2 and 3 [33]. Aromatic skeleton PCTTs are a subgroup of quinonemethides, which are characterized by the aromaticity of the A ring. Between 2001 and 2021 about 22 quinonemethides (**Q**), **Q1**–**Q22** (Figure 5), and 29 aromatics analogues (**A**), **A1**–**A29** (Figure 6) were isolated from Celastraceae species. 

In the ^13^C NMR spectra of the quinonemethides, signals are observed in the characteristic carbonyl region, between *δ*_C_ 170–200 ppm, and in the typical olefinic carbon region, around *δ*_C_ 110–160 ppm.

### 2.3. Dimers

Dimers are formed from PCTTs of the quinonemethide class and its aromatic derivatives, therefore they are also restricted to the Celastraceae family. According to Bazzocchi, Núñez and Reyes [31], these triterpenes are possibly biosynthesized through a Diels-Alder reaction, in which the different possible orientations of the monomers during the reaction result in a variety of isomers.

**Figure 7:**
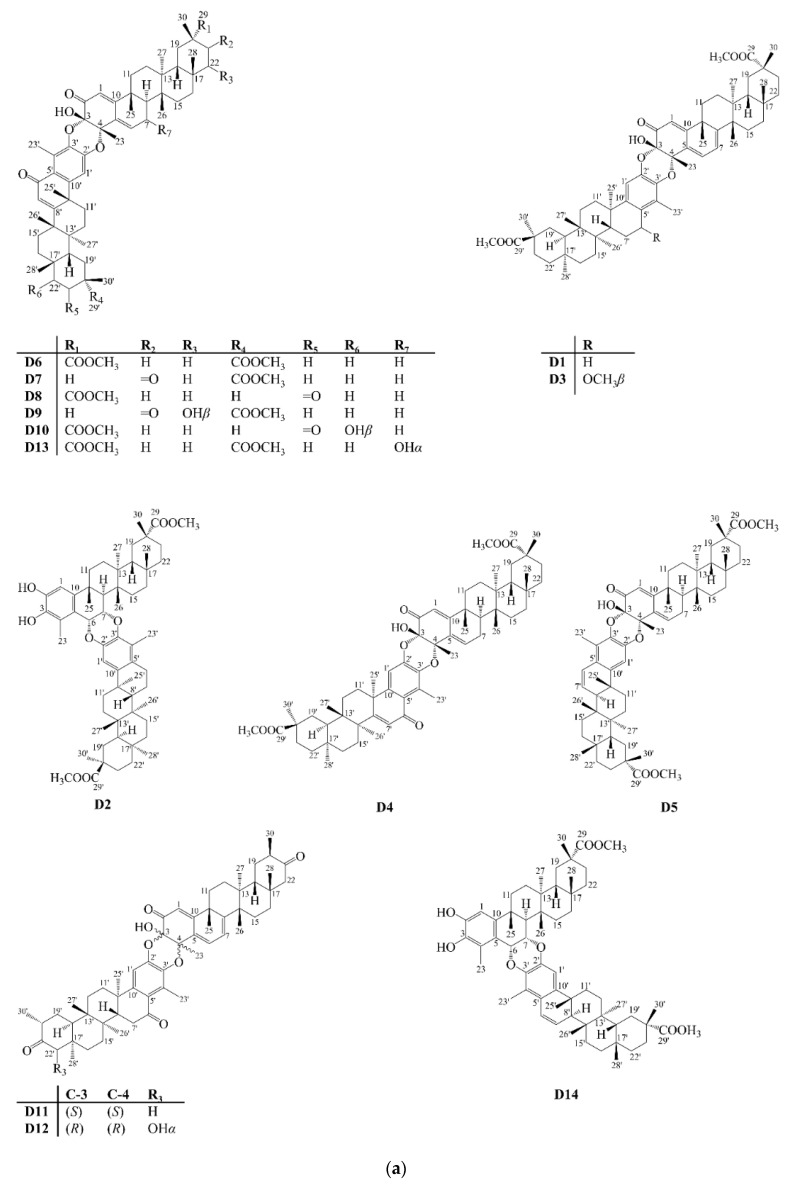

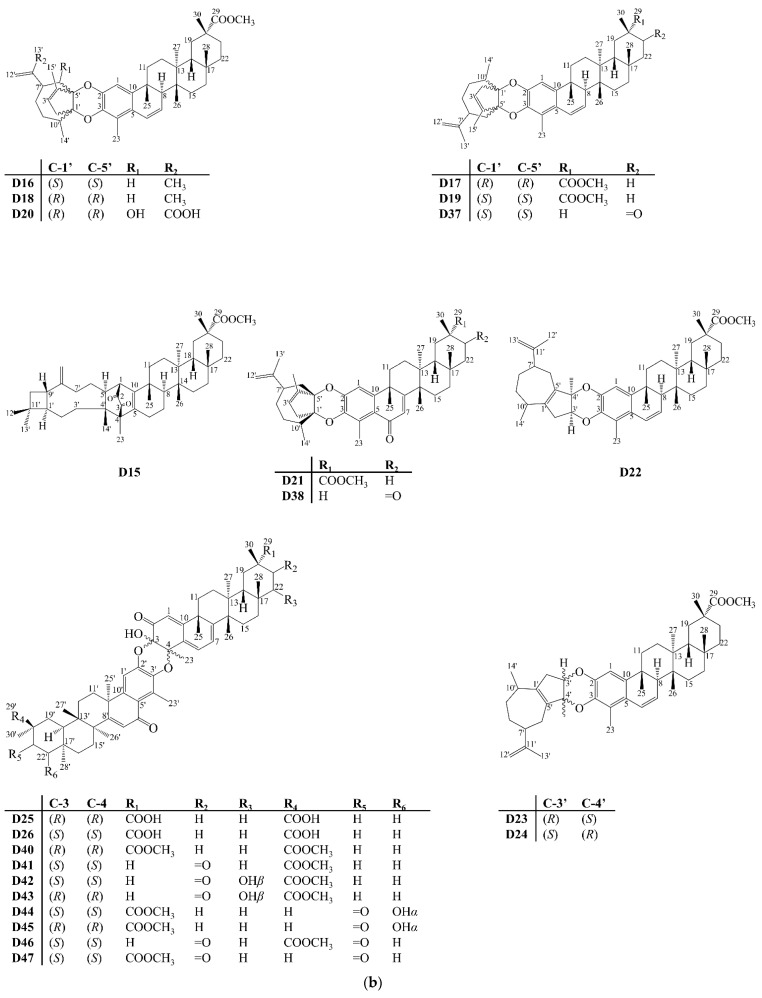

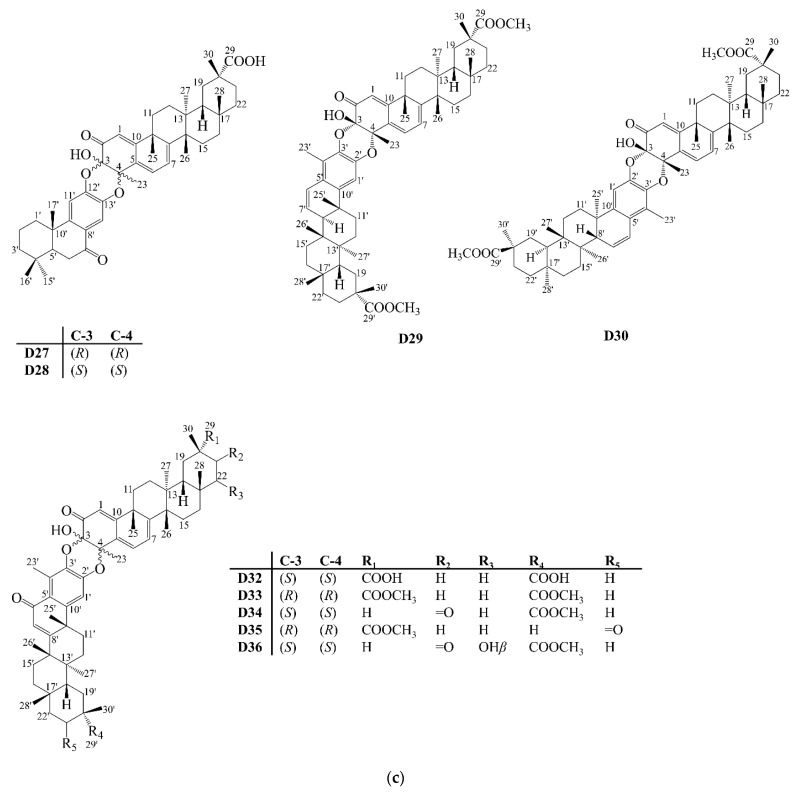

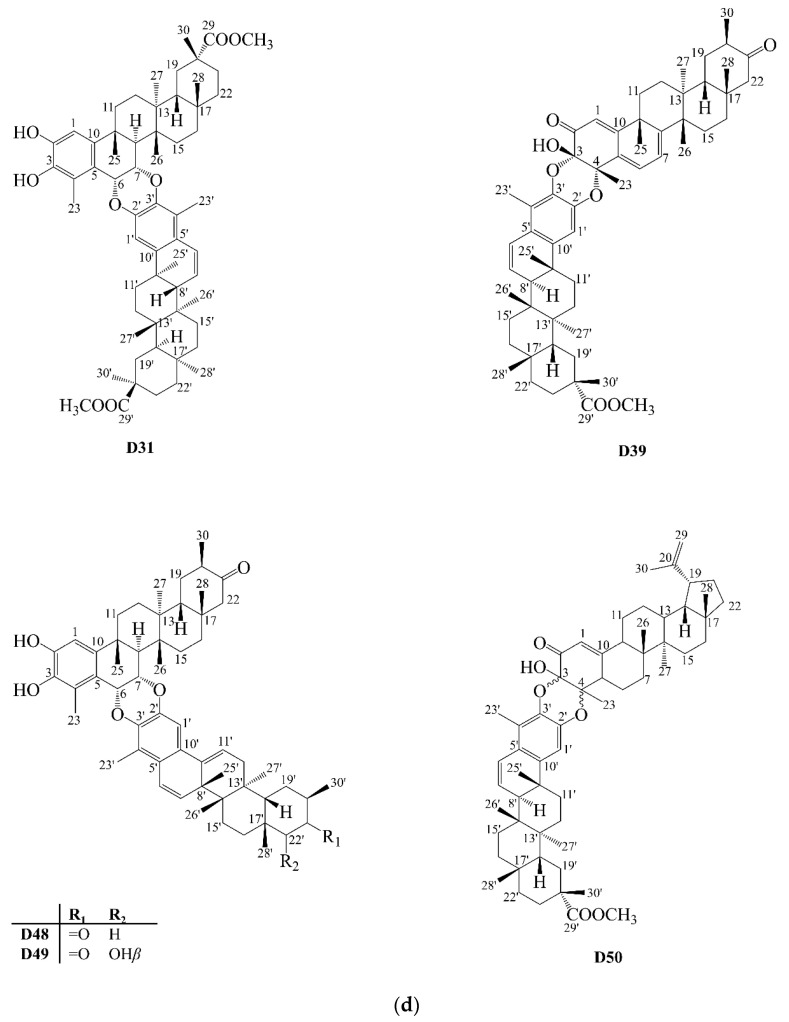
Structures of dimer-type pentacyclic triterpenoids isolated from Celastraceae species (2001–2021). (**a**) Compounds **D1**–**D14**. (**b**) Compounds **D15**–**D26**, **D37**, **D38** and **D40**–**D47**. (**c**) Compounds **D27**–**D30** and **D32**–**D36**. (**d**) Compounds **D31**, **D39** and **D48**–**D50**.

Between the years 2001 and 2021, 50 dimers (**D**), **D1**–**D50**, were reported (Figure 7). Most of these dimers are formed by two triterpenes with quinonemethide skeleton or their aromatic derivatives. However, the formation of adducts can also occur from the combination of a triterpene and a sesquiterpene (**D15**–**D24**; **D37**–**D38**).

### 2.4. Lupanes

**Figure 8:**
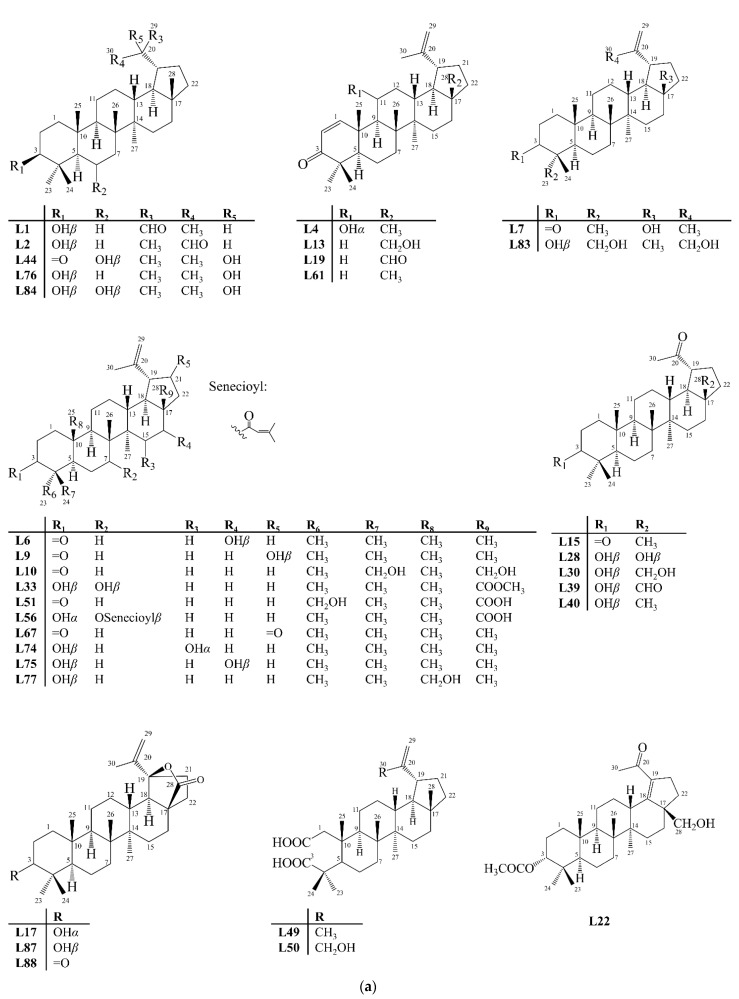

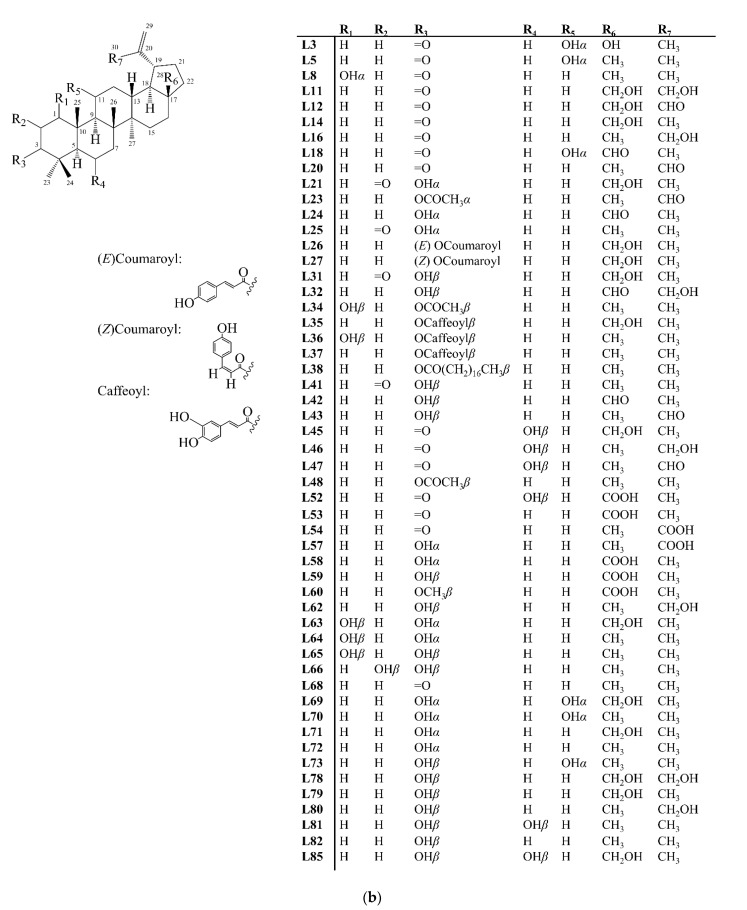

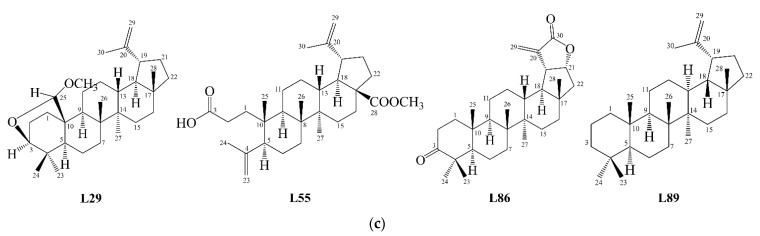
Structures of lupane-type pentacyclic triterpenoids isolated from Celastraceae species (2001–2021). (**a**) Compounds **L1**, **L2**, **L4**, **L6**, **L7**, **L9**, **L10**, **L13**, **L15**, **L17**, **L19**, **L22**, **L28**, **L30**, **L33**, **L39**, **L40**, **L44**, **L49-L51**, **L56**, **L61**, **L67**, **L74**–**L77**, **L83**, **L84**, **L87** and **L88**. (**b**) Compounds **L3**, **L5**, **L8**, **L11**, **L12**, **L14**, **L16**, **L18**, **L20**, **L21**, **L23**–**27**, **L31**, **L32**, **L34**–**L38**, **L41**–**L43**, **L45**–**L48**, **L52**–**L54**, **L57**–**L60**, **L62**–**L66**, **L68**–**L73**, **L78**–**L82** and **L85**. (**c**) Compounds **L29**, **L55**, **L86** and **L89**.

Unlike other skeletons, lupane-type PCTTs are formed by a *trans* pentacyclic ring system, in which the E ring is five-membered with an isopropenyl *α* substituent at carbon 19, containing a double bond between carbons 20 and 29 [19,22]. They have seven methyl groups, with two geminal ones attached to carbon 4 (Me 23*α* and 24*β*) and the others attached to carbon 8 (Me 26*β*), 10 (Me 25*β*), 14 (Me 27*α*), 17 (Me 28*β*), and 20 (Me 30), respectively. In this review, 89 pentacyclic triterpenoids of the lupane-type (**L**), **L1**–**L89**, were reported (Figure 8).

Characteristic ^13^C-NMR signals of the class of lupanes are those in the olefinic region, which appear around *δ*_C_ 109 (C-29) and δ_C_ 150 ppm (C-20), and signals from the methine carbons C-5 (H*α*), C- 9 (H*α*), C-13 (H*β*), C-18 (H*α*), and C-19 (H*β*), observed around δ_C_ 55, 50, 38, 48 and 47 ppm, respectively.

### 2.5. Oleananes

**Figure 9:**
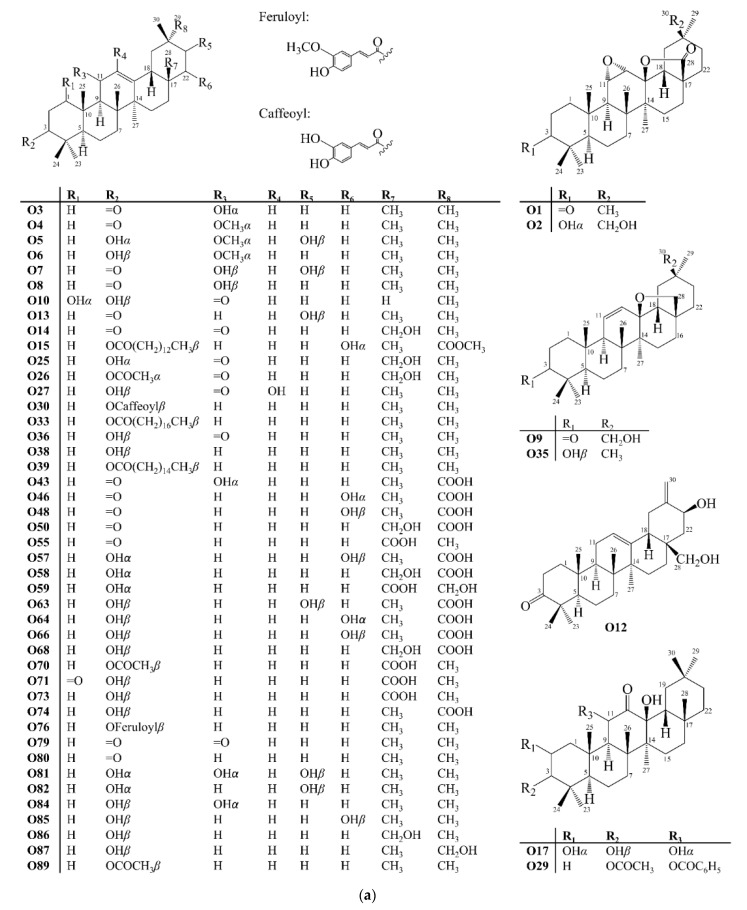

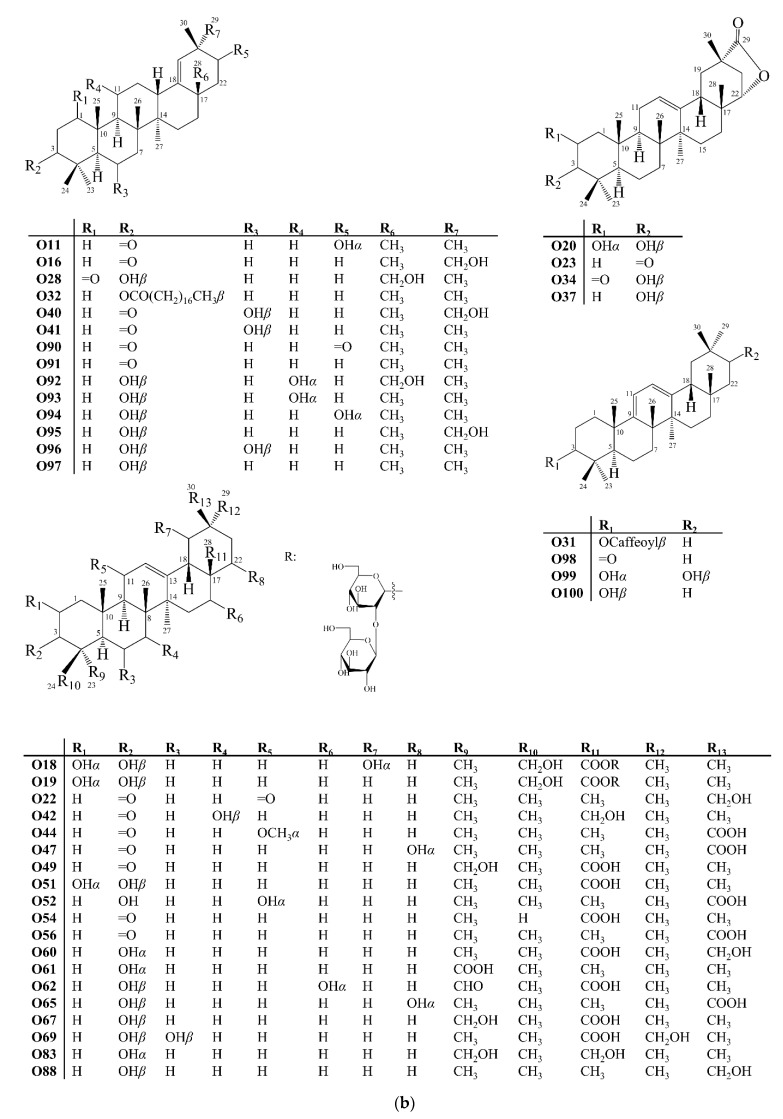

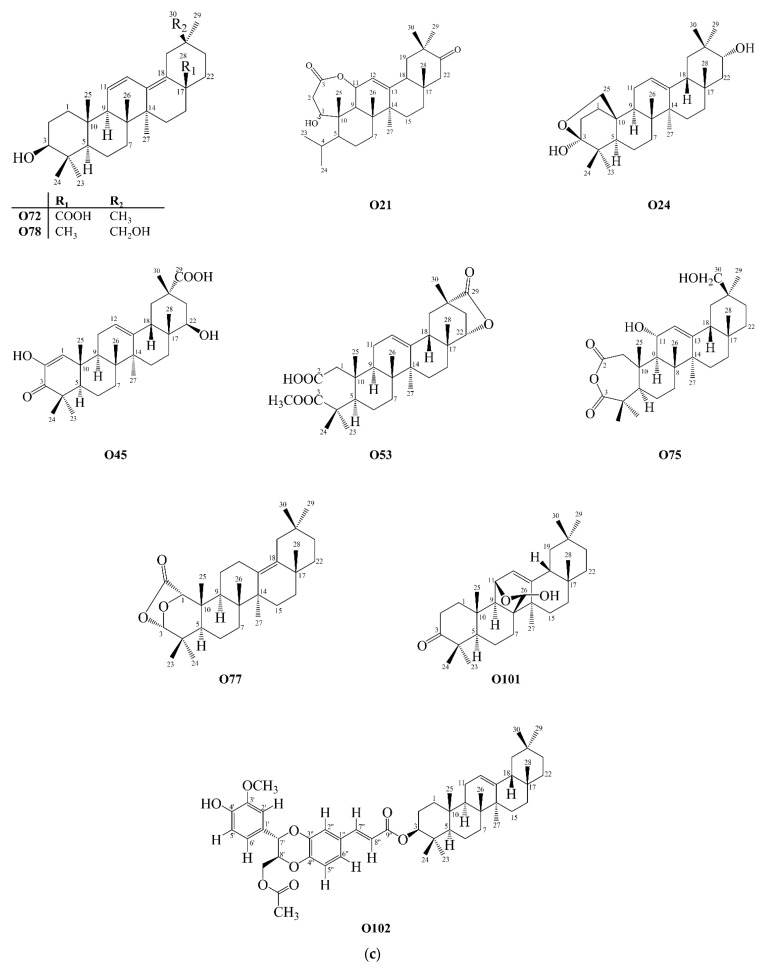
Structures of oleanane-type pentacyclic triterpenoids isolated from Celastraceae species (2001–2021). (**a**) Compounds **O1**–**O10**, **O12**–**O15**, **O17**, **O25**–**O27**, **O29**, **O30**, **O33**, **O35**, **O36**, **O38**, **O39**, **O43**, **O46**, **O48**, **O50**, **O55**, **O57**–**O59**, **O63**, **O64**, **O66**, **O68**, **O70**, **O71**, **O73**, **O74**, **O76**, **O79**–**O82**, **O84**–**O87** and **O89**. (**b**) Compounds **O11**, **O16**, **O18**–**O20**, **O22**, **O23**, **O28**, **O31**, **O32**, **O34**, **O37**, **O40**–**O42**, **O44**, **O47**, **O49**, **O51**, **O52**, **O54**, **O56**, **O60**–**O62**, **O65**, **O67**, **O69**, **O83**, **O88** and **O90**–**O100**. (**c**) Compounds **O21**, **O24**, **O45**, **O53**, **O72**, **O75**, **O77**, **O78**, **O101**, and **O102**.

Oleanane-type triterpenoids are characterized by the presence of a double bond, most commonly between carbons 12 and 13. Rings A/B, B/C, and C/D have *trans* configuration, whereas rings D/E are *cis*. They have eight methyl groups. Geminal ones 23 (*α*) and 24 (*β*) are connected to carbon 4, and 29 (*α*) and 30 (*β*) to carbon 20. The others are connected to carbons 8 (Me 26*β*), 10 (Me 25*β*), 14 (Me 27*α*) and 17 (Me 28*β*) [19]. In this work, 102 pentacyclic triterpenoids with oleanane skeleton (**O**), **O1**–**O102**, were reported (Figure 9).

In the ^13^C-NMR spectrum, the signals that characterize oleananes are those related to the double bond carbon atoms. For the most common oleananes with double bond between carbons 12 and 13, the chemical shifts are observed around *δ*_C_ 122 (C-12) and *δ*_C_ 145 ppm (C-13), except for those that have substituents close to these carbons [27].

### 2.6. Ursanes 

**Figure 10:**
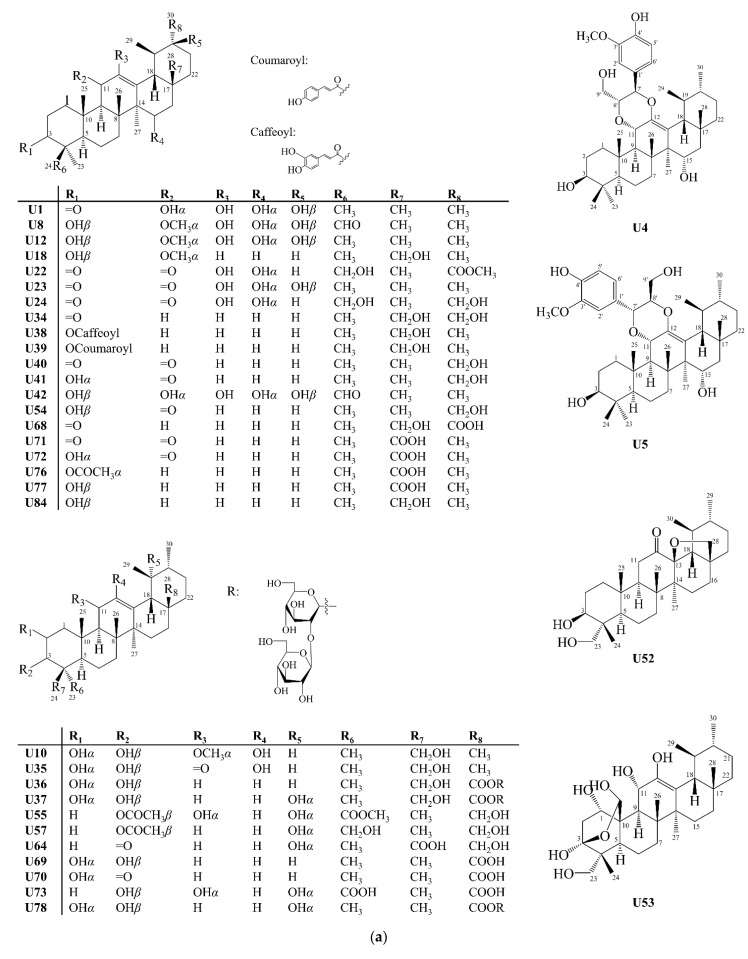

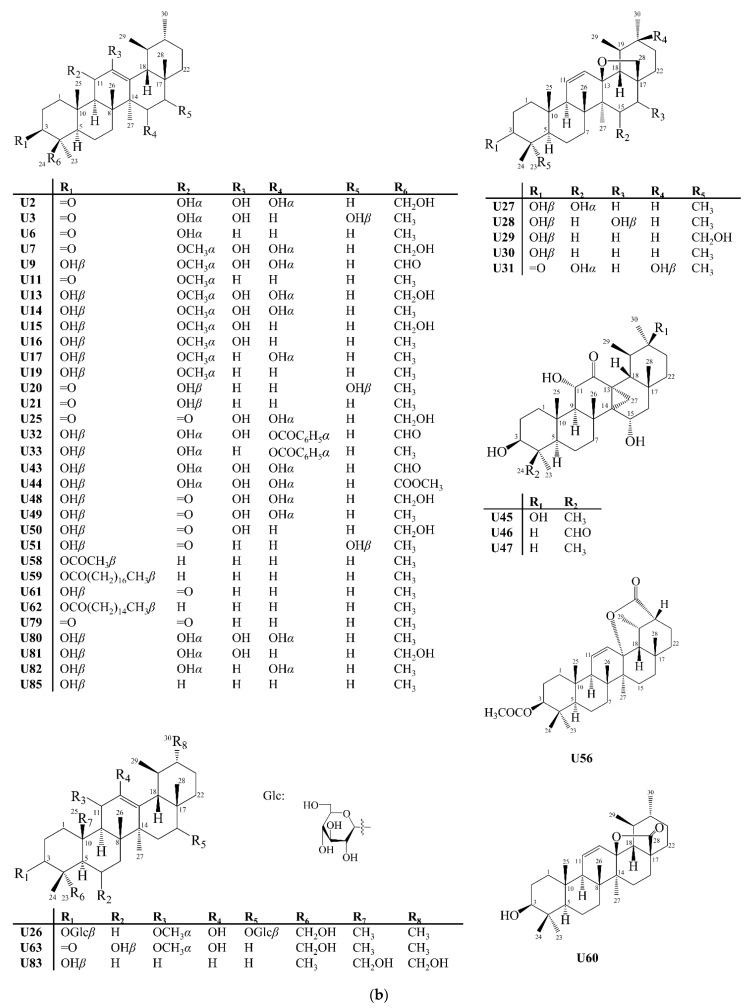

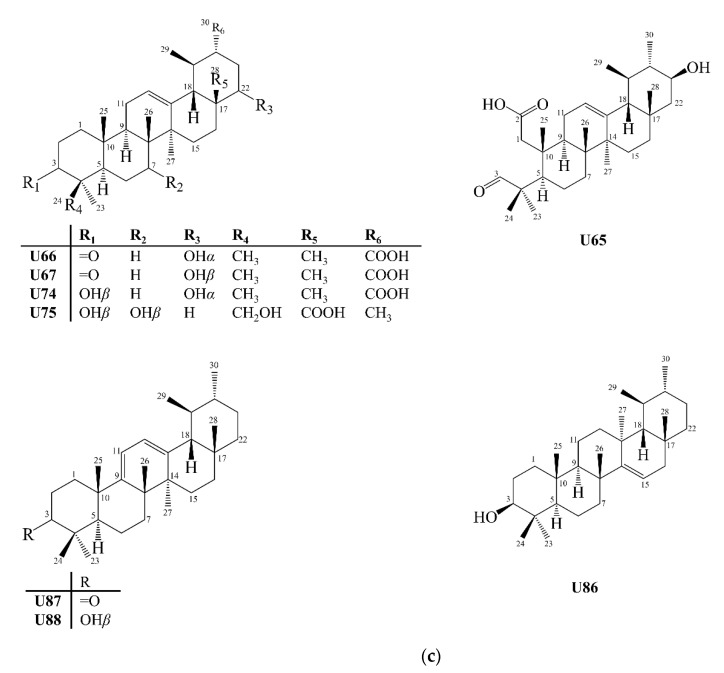
Structures of ursane-type pentacyclic triterpenoids isolated from Celastraceae species (2001–2021). (**a**) Compounds **U1**, **U4**, **U5**, **U8**, **U10**, **U12**, **U18**, **U22**–**U24**, **U34**–**42**, **U52**–**U55**, **U57**, **U64**, **U68**–**U73**, **U76**–**U78** and **U84**. (**b**) Compounds **U2**, **U3**, **U6**, **U7**, **U9**, **U11**, **U13**–**U17**, **U19**–**U21**, **U25**–**U33**, **U43**–**U51**, **U56**, **U58**–**U63**, **U79**–**U83 and U85**. (**c**) **U65**–**U67**, **U74**, **U75**, **U86**–**U88**.

Ursanes differ structurally from oleananes only by the position of methyl group 29, which is attached to carbon 19, in a *β* position. In the structure of ursanes, methyl group 30 is found in *α* position. Rings A/B, B/C and C/D have *trans* configuration, while rings D/E have *cis* configuration, like oleananes. The most common ursanes also present a double bond between carbons 12 and 13 [19]. There were 88 ursanes (**U**) isolated from Celastraceae species, triterpenoids **U1** to **U88**, were reported (Figure 10).

^13^C-NMR spectrum of ursanes differ from the spectrum of oleananes by the chemical shift signals of the olefinic carbon atoms, which are observed around *δ*_C_ 124 (C-12) and *δ*_C_ 139 ppm (C-13). In ursanes, the proximity of methyl group 29 with the double bond promotes a steric effect on these carbons, causing a shielding effect on C-13 and deshielding on C-12 [27,34]. This effect can be observed by comparing the ^13^C-NMR data of **O3** and **U6**, for example. Additionally, the number of quaternary carbon signals also represents a distinction parameter between these two skeletons, since 6 signals are observed in the oleananes spectrum and 5 signals in the ursanes spectrum.

### 2.7. Other Triterpenoid Skeletons Isolated from Celastraceae

**Figure 11:**
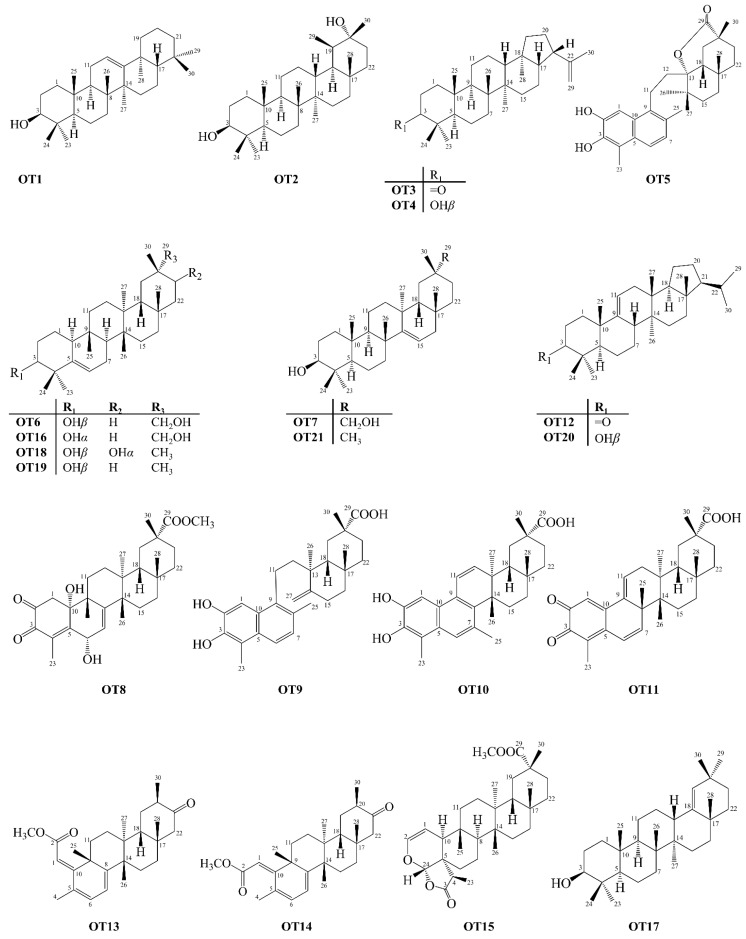
Structures of pentacyclic triterpenoids classified as others isolated from Celastraceae species (2001–2021).

In addition to the PCTT types described above, other 21 types of pentacyclic structures were also isolated from Celastraceae species (Figure 11). The terpenoid skeletons are gammacerane (**OT1**), taraxane (**OT2**), hopane (**OT3**, **OT4**), glutinane (**OT6**, **OT16**, **OT18**, **OT19**), taraxerane (**OT7**, **OT21**), germanicane (**OT17**) and unidentified types (**OT5**, **OT8**, **OT9**, **OT10**, **OT11**, **OT12**, **OT13**, **OT14**, **OT15**, **OT20**).

## 3. ^13^C-NMR Data of Pentacyclic Triterpenoids Isolated from Celastraceae Species (2001–2021)

Table 1, Table 2, Table 3, Table 4, Table 5, Table 6, Table 7 and Table 8 list the literature ^13^C-NMR data of the PCTTs that were isolated and characterized in the period of 2001–2021.

## 4. Conclusions

This review describes 504 pentacyclic triterpenoids isolated from Celastraceae species, classified as aromatics (29), dimers (50), friedelanes (103), lupanes (89), oleananes (102), quinonemethides (22), ursanes (88) and others (21). The data reported highlights the abundance and structural diversity of pentacyclic triterpenes isolated from plants of this family. The chemical complexity of these compounds helps to rationalize the various biological properties associated with these plant species, as well as these pure metabolites. The compilation of PCTTs ^13^C-NMR data presented in this review represents a contribution to the structural elucidation of new compounds of this class of terpenes.

## Figures and Tables

**Table 1:** ^13^C-NMR data of friedelane-type pentacyclic triterpenoids isolated from Celastraceae species (2001–2021).

C	F1	F2	F3	F4 ^a^	F6 ^b^	F7	F8 ^b^	F9	F10	F11
1	25.0	22.3	22.3	22.3	22.8	22.3	202.8	22.2	202.4	22.8
2	41.6	41.4	41.2	41.4	42.7	41.5	60.6	41.4	60.4	41.9
3	213.3	212.7	212.3	212.7	211.9	213.0	204.0	213.0	203.8	212.1
4	52.1	58.1	58.0	58.1	57.8	58.2	58.9	58.1	58.6	58.3
5	43.1	41.8	41.8	41.9	42.4	42.0	38.1	42.0	37.4	42.4
6	42.2	41.1	40.8	41.2	41.7	41.3	41.5	41.1	40.2	41.5
7	17.9	18.3	18.4	18.1	22.7	20.0	20.4	18.3	17.9	18.9
8	52.8	51.3	52.0	52.9	54.4	53.5	52.4	49.9	48.8	50.8
9	44.1	38.3	38.1	38.2	38.0	37.8	37.1	37.4	36.6	38.0
10	60.1	59.3	59.1	59.4	59.8	59.4	72.3	59.5	71.5	59.8
11	76.9	47.0	47.3	47.4	37.1	35.6	34.9	34.9	33.7	35.6
12	42.0	72.1	71.2	72.7	31.6	31.2	29.9	29.3	29.1	30.4
13	41.1	44.8	45.7	45.3	41.0	40.6	39.7	39.9	39.3	39.9
14	38.2	41.1	40.8	40.4	46.8	44.1	42.9	39.2	38.8	40.2
15	32.4	31.6	50.3	33.4	75.5	74.6	31.3	36.8	38.7	40.2
16	35.9	35.9	218.3	36.1	48.4	48.4	78.1	74.3	75.5	75.7
17	30.0	31.3	45.7	30.8	30.9	30.2	35.0	41.0	37.0	37.7
18	42.5	44.3	45.4	44.2	42.6	41.6	45.5	41.7	45.6	46.66
19	35.4	31.7	38.5	38.4	32.2	35.6	35.7	33.7	33.8	34.3
20	28.1	33.3	27.8	28.4	28.4	28.1	28.1	28.0	27.7	28.6
21	32.7	29.9	31.5	32.7	36.2	31.9	31.8	33.8	33.2	35.0
22	39.2	38.1	30.7	39.6	39.5	38.7	34.9	19.8	26.2	27.8
23	6.9	6.8	6.8	6.8	7.2	6.8	7.3	6.7	7.0	7.5
24	14.8	14.6	14.6	14.6	14.4	14.5	15.8	14.7	15.7	15.1
25	12.9	19.2	18.7	19.3	16.9	17.9	18.2	19.2	18.8	19.4
26	20.1	18.7	20.5	20.5	65.8	14.0	63.2	16.5	16.8	17.7
27	19.5	11.6	9.0	11.6	19.7	18.7	20.9	19.7	19.1	20.0
28	32.0	31.7	27.3	31.8	32.7	32.6	25.9	71.3	29.9	31.0
29	31.7	71.6	31.3	34.9	35.7	30.9	37.8	37.0	31.8	32.7
30	35.0	29.2	35.0	31.9	31.0	35.6	30.4	31.8	36.2	36.9
C=O							171.3			
OCH_3_							21.3			
Ref	[35]	[36]	[37]	[38]	[39]	[40]	[41]	[42]	[43]	[44]
**C**	**F12 ^a^**	**F13**	**F14 ^b^**	**F15 ^a^**	**F16**	**F17**	**F18**	**F19**	**F20**	**F21**
1	21.7	22.2	22.3	68.8	75.8	74.0	71.4	202.7	22.3	22.3
2	40.9	41.4	41.6	51.9	35.0	30.1	52.7	60.6	41.5	41.3
3	211.1	212.8	212.5	210.4	212.3	213.2	211.3	204.1	213.2	212.7
4	57.4	58.0	58.3	57.5	55.9	53.3	59.1	59.1	58.2	58.1
5	41.5	42.1	42.3	39.4	43.3	42.7	43.9	37.8	42.0	41.8
6	40.5	41.1	41.4	41.8	41.8	41.2	34.3	40.6	41.2	40.2
7	18.0	18.4	18.6	17.8	17.9	18.2	18.8	18.1	18.0	21.2
8	53.0	53.3	53.5	53.4	53.8	53.0	53.9	50.6	51.2	44.7
9	36.9	37.4	37.6	36.6	42.0	36.9	38.6	37.3	37.5	37.9
10	58.7	59.3	59.7	64.8	57.4	52.4	62.6	71.9	59.5	59.0
11	35.1	35.6	35.8	37.0	30.2	35.9	35.7	34.2	35.3	36.8
12	30.0	30.7	30.8	29.9	31.0	30.5	30.4	29.8	30.2	33.9
13	39.6	40.1	39.3	39.4	39.8	39.8	39.8	38.8	39.0	47.5
14	38.8	39.1	40.1	39.3	37.5	38.4	38.5	38.7	38.8	56.3
15	44.7	44.3	44.4	33.2	32.7	32.1	32.7	30.5	30.4	213.3
16	75.7	74.4	75.6	36.0	36.0	35.4	36.2	35.9	36.1	53.8
17	40.5	36.4	32.1	30.4	29.9	30.0	30.2	32.5	32.5	34.0
18	39.9	44.1	44.8	41.9	42.7	42.7	43.0	44.2	44.3	45.3
19	29.3	30.4	35.8	30.8	35.3	28.9	35.5	36.0	35.9	38.9
20	32.7	33.1	28.0	33.2	28.1	33.4	28.2	34.3	34.4	28.0
21	27.7	27.5	32.1	27.8	32.7	28.2	33.0	74.3	74.3	71.7
22	29.5	36.4	36.0	39.7	39.3	28.1	39.3	47.0	47.0	46.3
23	6.4	6.9	6.8	7.1	6.7	6.9	6.9	7.3	6.8	6.8
24	14.0	14.6	14.7	16.0	14.7	14.3	17.4	16.0	14.6	14.9
25	17.5	18.1	18.2	18.4	63.0	17.9	19.2	18.2	17.7	18.9
26	21.1	20.1	20.1	18.6	20.1	20.0	20.2	17.8	18.2	15.4
27	19.3	21.4	21.5	21.0	18.6	18.7	18.7	19.3	19.3	12.1
28	66.7	25.4	24.9	32.1	32.2	32.2	32.2	33.1	33.2	31.5
29	74.0	74.4	30.8	74.5	35.0	28.9	31.8	24.9	31.9	31.4
30	25.4	25.7	35.5	25.9	31.7	72.0	34.9	31.8	24.9	33.8
Ref	[45]	[46]	[27]	[45]	[47]	[48]	[48]	[49]	[50]	[51]
**C**	**F22**	**F23**	**F24**	**F25**	**F26**	**F27**	**F28 ^b^**	**F29**	**F30**	**F31**
1	22.4	202.8	24.7	24.6	202.8	22.5	22.4	22.3	202.7	22.1
2	41.6	60.5	42.6	42.5	60.6	41.6	41.6	41.5	60.6	41.3
3	213.2	230.7	212.9	212.8	204.1	213.3	211.7	212.9	204.1	213.6
4	58.4	59.7	58.5	58.5	58.9	58.4	57.9	58.3	59.0	57.8
5	42.4	37.5	42.4	42.4	38.1	42.3	42.1	42.1	37.2	41.9
6	41.4	38.5	41.8	41.7	41.7	41.5	41.2	41.4	40.6	41.0
7	18.4	17.0	17.9	18.0	20.4	18.6	18.4	18.3	18.0	18.1
8	53.4	45.2	53.7	53.9	52.0	53.8	53.2	53.5	51.5	52.2
9	37.6	37.1	42.0	42.0	37.1	37.6	37.5	37.5	37.8	37.3
10	59.6	69.0	60.7	60.7	72.4	59.7	59.2	59.6	71.9	59.1
11	35.8	35.8	29.9	30.0	35.0	37.8	35.7	35.5	33.4	35.3
12	30.8	26.5	31.2	31.4	29.9	24.1	30.5	30.0	29.7	29.9
13	39.8	38.1	39.7	39.9	39.7	45.4	40.1	39.6	39.2	39.1
14	38.4	40.0	37.7	37.9	42.0	38.4	38.2	38.4	39.1	38.0
15	32.5	34.7	32.7	33.0	20.1	32.2	29.5	31.1	31.2	31.3 *
16	38.8	36.8	36.0	35.0	35.3	36.2	32.6	29.2	29.0	29.0
17	30.0	30.8	30.1	33.1	30.4	30.3	36.6	35.1	35.1	35.1
18	42.8	44.0	42.7	41.9	43.5	43.3	39.0	38.9	39.3	39.2
19	36.0	35.4	35.3	37.0	35.4	37.1	29.8	31.5	34.4	34.4
20	33.9	28.3	28.1	42.7	28.3	28.5	33.6	33.3	28.1	27.9
21	75.8	32.5	32.7	218.7	32.9	32.6	29.2	30.2	31.4	31.4 *
22	48.6	38.9	39.2	55.0	39.1	40.1	33.0	28.3	34.3	33.2
23	6.9	7.5	7.0	7.0	7.3	7.1	7.2	6.8	7.3	6.7
24	14.8	15.7	14.7	14.7	15.7	14.9	14.7	14.7	15.9	14.5
25	18.0	67.1	63.0	62.7	17.8	18.2	18.0	18.2	18.1	18.0
26	20.8	69.9	20.1	20.9	64.0	22.3	20.1	18.6	19.1	18.9
27	18.9	19.3	18.5	18.4	19.7	63.4	19.0	19.1	19.2	19.1
28	34.5	30.0	32.1	33.6	31.7	32.8	67.1	69.0	68.0	67.0
29	34.9	31.4	35.0	28.8	34.5	35.8	73.6	28.6	34.2	32.9
30	24.1	35.1	31.7	25.0	32.0	30.6	27.5	73.4	32.8	34.2
Ref	[52]	[53]	[48]	[54]	[41]	[55]	[56]	[57]	[58]	[57]
**C**	**F32**	**F34**	**F35**	**F36**	**F37**	**F38**	**F39^c^**	**F40**	**F41**	**F42**
1	22.7	22.2	22.5	25.9	32.4	31.7	28.2	24.8	202.7	22.3
2	41.4	41.3	41.6	75.4	76.9	74.4	71.0	42.6	60.6	41.5
3	213.4	212.2	212.8	105.6	212.0	209.7	199.6	212.3	204.1	212.9
4	58.6	57.9	58.6	47.4	52.7	52.4	127.1	58.6	59.1	58.3
5	42.3	42.0	42.4	46.8	42.7	54.2	158.8	42.3	37.8	42.1
6	41.9	40.8	41.7	33.3	40.8	36.7	30.4	41.6	40.6	41.4
7	18.4	18.5	18.5	19.2	17.5	19.4	20.3	18.2	18.0	18.3
8	53.2	52.3	53.7	49.8	52.7	49.6	47.3	54.0	52.0	53.1
9	38.0	37.5	37.8	37.1	38.0	37.5	37.2	41.1	37.2	37.5
10	59.6	59.0	59.9	53.2	52.7	55.7	51.8	60.3	71.9	59.6
11	35.7	35.2	36.0	34.3	35.6	34.5	32.4	30.1	34.5	35.7
12	31.0	28.9	30.8	29.0	30.2	29.4	28.9	30.7	30.1	29.4
13	37.6	39.0	38.6	39.2	39.4	40.2	39.2	39.9	39.6	39.9
14	39.3	40.6	40.3	39.1	38.9	39.4	39.5	37.9	38.3	38.2
15	28.6	50.2	33.0	29.1	32.7	28.3	27.9	33.1	32.1	32.2
16	32.8	218.4	36.2	36.2	36.5	29.6	35.1	34.9	35.8	29.7
17	48.6	45.7	30.8	30.1	29.8	44.8	37.7	33.0	30.0	30.0
18	35.9	43.2	42.3	44.5	42.4	45.4	43.4	41.8	42.6	42.9
19	29.8	30.1	39.8	30.4	35.0	31.6	31.3	36.9	29.4	30.6
20	33.7	32.7	33.4	40.6	27.8	41.4	41.9	42.7	33.4	33.4
21	28.0	27.0	30.0	30.0	32.0	214.2	213.7	218.7	28.1	28.3
22	33.7	31.1	28.1	36.6	40.8	77.5	53.3	55.0	38.1	39.9
23	7.2	6.9	7.0	7.1	6.3	7.9	10.7	7.0	7.3	6.8
24	15.0	14.6	14.9	72.4	13.8	174.2		14.8	16.0	14.7
25	17.5	17.2	18.1	16.7	17.9	16.8	17.0	65.1	18.1	18.0
26	20.5	20.3	18.6	16.2	18.4	15.5	15.0	21.5	20.0	18.6
27	19.2	15.9	20.9	17.5	19.9	19.4	17.9	18.6	18.6	19.9
28	208.9	27.4	32.3	31.9	32.1	25.4	32.3	33.8	32.1	32.2
29	24.4	74.1	75.0	179.4	31.7			28.8	28.9	29.0
30	74.7	25.8	26.1	32.1	34.7	15.0	14.7	25.0	72.0	72.1
OCH_3_						51.7				
C=O								166.8		
*Iso*								130.2		
*Orto*								129.5		
*Meta*								128.6		
*Para*								133.1		
Ref	[48]	[46]	[59]	[48]	[60]	[61]	[62]	[54]	[49]	[57]
**C**	**F43**	**F44**	**F45**	**F47**	**F49**	**F50**	**F51**	**F52**	**F53**	**F54**
1	31.3	200.5	27.6	22.2	22.3	39.0	36.1	26.7	26.7	210.9
2	193.1	102.6	41.2	41.5	41.5	211.4	211.7	72.7	73.2	53.9
3	146.5	175.5	213.0	213.0	213.0	77.2	77.0	106.3	106.8	75.4
4	126.9	49.9	57.9	58.2	58.2	54.8	54.6	45.6	45.7	50.0
5	54.7	41.6	41.7	42.1	42.1	38.0	38.1	52.8	47.0	44.5
6	30.7	41.1	40.7	41.2	41.3	41.1	40.7	72.8	33.6	42.7
7	18.4	17.6	17.8	18.0	18.2	17.3	17.6	30.4	19.2	18.2
8	49.4	52.3	51.5	52.8	50.4	53.8	53.2	46.5	49.7	53.2
9	37.0	37.1	37.4	37.1	37.5	41.8	37.7	37.0	37.0	37.2
10	55.8	68.7	59.0	59.2	59.6	60.8	60.5	52.7	52.6	71.8
11	33.1	35.3	34.8	35.4	35.2	29.5	35.1	34.0	34.1	35.2
12	29.2	30.4	30.4	30.6	30.5	30.5	20.3	29.7	29.0	30.9
13	39.2	39.4	37.5	38.7	39.3 *	39.7	39.7	39.2	39.1	40.6
14	39.1	38.3	38.4	37.6	39.4 *	38.2	38.4	38.9	49.1	38.8
15	28.9	32.5	32.6	32.4	30.0	32.7	32.4	29.1	29.0	33.3
16	36.0	36.0	29.3	34.9	36.2	35.9	36.0	36.1	36.2	36.6
17	30.1	29.9	42.4	47.7	30.2	30.0	30.0	30.5	30.1	31.1
18	44.4	42.7	38.5	36.4	44.6	42.6	42.9	44.5	44.5	42.6
19	30.4	35.1	34.1	35.4	29.2	35.3	35.4	30.1	30.4	30.7
20	40.5	28.2	32.8	28.3	40.6	28.1	28.2	40.5	40.5	34.0
21	29.9	32.8	31.0	32.4	29.6	32.7	32.8	30.0	30.0	28.7
22	36.5	39.3	22.0	28.0	36.6	39.2	39.3	36.5	36.4	40.5
23	10.6	8.5	6.6	6.8	6.8	10.9	10.8	9.9	6.9	12.4
24	194.9	15.1	14.3	14.6	14.6	14.0	14.2	64.6	72.2	18.4
25	17.4	18.1	17.0	17.2	17.5	63.7	17.4	16.5	16.7	19.0
26	15.9	20.4	19.0	20.0	18.5	20.3	20.2	16.1	16.1	21.3
27	17.2	18.7	16.1	18.8	16.1	18.5	18.5	1.4	17.4	19.1
28	31.8	32.0	177.0	209.1	31.9	32.2	31.1	31.8	31.8	32.7
29	179.1	35.0	26.7	34.5	179.3	35.0	31.8	179.1	179.1	74.7
30	31.9	31.7	79.7	29.4	31.9	31.7	35.0	32.0	32.0	26.9
OCH_3_	51.5	55.6			51.5					
Ref	[63]	[64]	[48]	[65]	[66]	[47]	[67]	[68]	[48]	[69]
**C**	**F55**	**F56**	**F57**	**F59 ^b^**	**F60**	**F61**	**F62**	**F63**	**F64 ^b^**	**F65 ^c^**
1	21.7	29.6	146.3	35.6	21.9	22.3	22.4	74.0	28.7	26.6
2	37.1	72.4	130.1	41.1	41.2	41.7	41.7	29.7	74.2	68.5
3	216.6	202.3	200.9	212.4	212.4	212.8	212.7	231.2	108.1	71.2
4	58.7	129.5	57.7	58.1	58.2	58.8	58.8	53.3	46.8	42.8
5	39.9	157.8	49.1	42.5	42.6	41.3	41.3	42.7	47.5	49.0
6	37.4	65.4	77.2	50.4	52.2	49.0	48.9	41.2	34.0	37.2
7	17.7	29.9	28.7	69.2	68.6	68.4	68.3	18.1	19.7	19.0
8	53.5	40.9	48.2	58.2	58.6	52.9	52.6	53.1	50.4	49.2
9	37.0	38.5	36.7	38.8	39.0	37.2	37.6	36.9	37.5	44.0
10	49.4	48.9	60.3	59.3	58.9	60.0	59.8	52.4	53.3	50.0
11	35.7	33.4	45.4	36.0	35.9	37.6	31.2	32.7	34.7	33.8
12	30.5	29.5	69.3	29.9	29.6	30.5	37.5	29.5	29.5	28.8
13	39.7	39.3	40.0	40.3	40.3	40.4	39.5	39.7	39.6	39.6
14	38.3	40.6	45.6	44.2	40.0	39.1	44.9	38.0	39.3	38.8
15	32.4	28.3	49.6	27.0	29.5	32.4	32.8	30.2	29.7	27.8
16	36.0	29.8	214.2	35.5	38.3	35.8	29.1	35.3	36.7	28.9
17	30.0	45.2	47.1	30.1	30.4	30.5	38.3	30.0	30.5	36.4
18	42.7	45.8	44.7	43.3	42.3	41.6	38.0	42.4	44.8	45.0
19	35.3	32.0	39.6	21.9	35.3	29.6	35.1	31.3	30.9	30.9
20	28.1	41.7	42.3	28.3	33.5	33.1	28.5	40.2	40.7	40.8
21	32.7	214.1	218.1	32.4	27.9	27.9	35.9	28.2	30.5	213.2
22	39.2	78.1	47.2	39.2	36.1	39.4	32.5	38.2	37.4	76.7
23	13.5	11.1	9.8	6.9	6.9	6.9	6.9	6.8	8.4	11.3
24	23.1		8.7	15.9	15.9	16.1	16.1	14.4	72.1	176.5
25	18.0	17.0	19.6	18.8	18.9	19.0	18.8	17.5	16.9	15.2
26	20.4	15.8	19.9	64.2	13.1	21.8	21.4	20.9	16.8	17.3
27	18.7	19.3	8.7	20.1	20.8	18.3	18.5	17.7	18.1	18.6
28	32.1	25.9	29.1	31.2	31.8	32.1	179.2	32.0	32.1	25.5
29	35.0		28.4	34.7	29.1	74.5	34.4	31.8	181.3	
30	31.7	15.4	24.4	31.1	71.3	26.0	29.6	183.1	32.3	14.9
Ref	[70]	[71]	[72]	[39]	[73]	[48]	[48]	[48]	[74]	[75]
**C**	**F66**	**F67 ^+^**	**F68**	**F69 ^b^**	**F70 ^b^**	**F71**	**F72**	**F73**	**F74**	**F75 ^b^**
1	30.8	28.2	32.6	30.9	39.0	22.3	22.2	22.3	36.5	20.3
2	74.1	76.5	75.0	193.5	194.6	41.5	42.0	41.5	212.3	38.9
3	213.3	208.1	212.4	149.0	144.2	213.2	213.3	213.3	77.3	105.9
4	52.7	54.3	55.6	125.8	139.9	58.2	58.2	58.3	54.8	53.7
5	43.1	43.1	43.1	54.9	40.0	42.1	44.7	42.0	39.6	47.0
6	41.3	41.0	41.2	32.7	33.6	41.2	41.5	41.3	40.9	33.9
7	18.5	18.2	18.1	18.9	18.2	18.2	18.4	18.2	17.9	19.4
8	50.9	50.4	53.2	49.5	50.8	53.1	52.9	50.7	51.0	50.0
9	37.2	36.6	37.4	37.1	37.8	37.5	37.7	37.4	38.3	37.3
10	52.6	53.3	56.5	55.5	56.1	59.4	59.2	59.8	60.9	57.1
11	35.4	35.1	35.3	33.3	35.0	35.6	35.9	36.1	35.0	34.6
12	30.5	30.1	30.2	30.9	30.7	30.3	31.0	29.5	29.7	29.4
13	39.5	39.2	39.7	39.4	39.7	39.7	41.0	39.2	38.0	39.4
14	39.7	39.3	38.1	39.6	39.8	38.0	38.8	39.1	39.5	39.2
15	29.8	30.4	32.8	30.5	30.6	32.8	29.3	29.4	30.1	29.3
16	37.8	36.2	35.4	36.6	36.8	35.4	32.6	36.6	36.5	36.5
17	30.4	29.1	29.6	30.5	31.2	29.5	37.6	30.1	30.5	30.3
18	44.8	44.5	42.5	44.8	45.0	42.5	37.6	44.2	44.7	44.6
19	30.8	36.9	31.3	29.5	29.9	31.2	35.4	29.3	30.6	30.7
20	40.7	40.6	40.3	40.7	40.9	40.3	28.4	40.4	40.6	40.5
21	29.7	29.9	28.2	29.4	30.1	28.2	34.8	30.2	29.7	30.3
22	36.7	29.4	38.2	37.4	37.2	38.2	32.6	35.2	37.1	37.2
23	6.8	6.5	6.5	10.7	10.5	6.8	6.8	6.9	11.2	8.4
24	14.2	14.1	14.7	195.8	19.1	14.6	14.6	14.6	14.6	72.9
25	18.0	18.3	17.8	17.2	18.2	17.7	17.5	18.4	18.1	16.7
26	18.5	16.1	20.9	17.9	18.5	20.9	20.7	16.3	18.2	17.9
27	16.1	17.5	17.7	16.2	17.0	17.7	18.5	18.0	16.6	16.5
28	32.1	31.8	31.8	32.3	32.0	32.0	185.0	31.8	32.2	31.9
29	181.2	31.9	183.3	181.5	181.2	31.9	29.7	184.8	32.1	181.2
30	32.1	179.4	32.0	32.1	32.3	184.7	34.5	31.5	182.4	32.2
COOCH_3_		179.4								
COOCH_3_		51.6								
COOCH_3_		169.8								
COOCH_3_		21.2								
Ref	[76]	[77]	[78]	[59]	[79]	[80]	[56]	[81]	[82]	[83]
**C**	**F76**	**F77 ^c^**	**F79**	**F80**	**F81**	**F82**	**F83**	**F84**	**F85**	**F86**
1	19.3	26.6	22.4	16.0	148.2	202.7	202.8	22.2	22.3	22.3
2	36.3	67.6	41.7	35.4	130.4	60.6	60.6	40.8	41.1	41.4
3	71.9	83.1	212.7	73.0	201.5	204.0	204.2	212.1	212.1	213.1
4	52.9	43.3	58.8	49.4	57.6	59.0	59.1	57.9	58.1	58.2
5	37.4	48.6	41.3	38.0	43.7	37.8	37.8	42.2	42.2	42.0
6	40.9	33.3	48.9	41.9	39.5	40.5	40.6	41.0	41.0	40.5
7	17.4	18.7	68.3	17.7	18.1	18.1	18.1	18.4	18.6	21.3
8	52.6	49.6	52.9	53.7	51.5	52.4	52.2	53.0*	53.1	45.3
9	37.0	36.8	37.6	37.3	36.8	37.2	37.2	55.5	43.8	37.2
10	59.6	52.2	59.8	61.5	61.9	71.8	71.9	53.1*	59.3	59.3
11	35.6	33.7	31.1	35.8	34.4	34.5	34.6	214.2	51.4	34.4
12	30.9	28.7	37.8	30.9	28.8	30.3	30.2	51.2	214.2	29.4
13	37.8	38.6	39.5	40.1	39.0	39.7	39.5	44.0	55.5	42.4
14	38.6	38.6	44.6	38.5	40.5	38.1	38.2	43.8	44.0	54.2
15	32.3	29.4	32.8	32.8	49.8	32.7	32.4	31.6	35.6	214.9
16	29.2	35.9	29.2	36.1	218.7	35.0	35.9	36.1	36.2	54.0
17	44.5	29.7	38.4	30.7	45.3	33.2	30.0	29.6	29.7	33.5
18	37.6	44.0	38.4	42.0	43.9	41.8	42.7	36.4	36.6	44.0
19	34.5	29.7	35.1	29.9	35.4	37.0	35.3	35.4	31.7	34.9
20	28.2	40.6	28.5	33.3	27.6	42.8	28.2	28.3	28.4	27.9
21	35.1	29.4	35.8	28.0	31.6	218.8	32.8	33.0	33.1	33.8
22	32.2	36.3	32.4	39.7	30.7	55.0	39.3	38.9	39.1	38.6
23	9.6	14.3	6.9	11.8	6.7	7.3	7.3	6.8	6.9	6.8
24	14.3	96.5	16.1	16.6	13.7	16.0	16.0	14.5	14.6	15.0
25	17.4	16.3	18.8	18.4	19.1	17.8	18.0	18.1	18.2	17.4
26	20.3	16.1	21.7	20.8	19.8	21.3	20.3	19.0	19.9	14.7
27	18.3	17.2	18.5	18.7	16.2	18.5	18.7	19.8	19.1	18.9
28	183.7	31.7	183.3	32.3	27.4	33.5	32.0	31.8	31.9	32.2
29	29.5	179.6	34.3	75.0	35.2	28.8	31.8	31.7	31.8	33.3
30	34.2	31.6	29.7	26.0	31.1	25.0	35.0	34.2	34.3	33.4
Ref	[48]	[84]	[48]	[69]	[45]	[52]	[85]	[86]	[35]	[51]
**C**	**F87**	**F88**	**F89**	**F90**	**F91 ^b^**	**F92**	**F93 ^a^**	**F94**	**F95**	**F96**
1	22.2	21.6	22.2	18.6	17.0	16.0	16.2	41.3	27.8	31.4
2	41.4	40.8	41.4	35.4	40.3	35.5	36.1	41.6	170.4	193.2
3	212.5	210.6	212.8	72.0	74.7	72.0	71.6	213.0	177.0	146.5
4	58.2	57.8	58.1	49.4	491	49.4	49.6	59.5	47.3	127.0
5	42.1	47.0	42.0	38.3	41.4	37.9	38.1	42.2	38.9	54.7
6	41.0	56.9	41.2	41.9	36.0	41.8	42.0	35.6	34.5	30.6
7	18.6	210.2	18.1	17.5	19.3	17.7	17.7	30.7	17.1	18.3
8	52.4	63.4	53.0	52.9	53.8	53.2	53.3	53.4	52.0	48.7
9	37.7	42.4	37.4	43.9	36.8	37.1	37.2	37.5	35.3	37.0
10	59.4	59.0	59.4	62.3	61.6	61.5	61.6	58.2	46.1	56.0
11	35.4	35.5	35.5	76.6	36.5	35.5	35.7	22.4 *	32.0	33.3
12	29.1	29.8	30.4	42.0	31.4	30.3	30.7	32.9	30.1	28.8
13	39.2	39.4	39.6	38.5	37.6	39.7	38.4	38.3	39.6	40.7
14	40.5	37.5	38.2	40.9	39.9	38.0	39.7	40.0	38.0	39.5
15	50.2	31.6	32.2	32.3	32.5	32.3	32.3	18.4 *	32.3	28.2
16	218.8	36.3	35.9	36.0	35.6	32.0	35.9	37.1	35.7	35.9
17	45.3	30.1	29.9	30.0	30.7	36.1	30.0	42.8	30.0	31.4
18	44.0	41.8	42.8	42.6	43.6	38.5	42.9	42.0	42.6	45.3
19	35.5	34.9	35.2	35.7	35.6	29.3	35.4	35.1	35.2	29.8
20	27.6	28.0	28.0	28.1	28.8	33.1	28.2	33.3	28.1	149.1
21	31.7	32.8	32.7	32.8	32.9	28.4	32.9	218.8	32.6	30.7
22	30.8	38.6	39.2	39.2	39.0	28.7	39.3	55.1	39.1	38.0
23	6.8	6.8	6.7	12.1	14.8	11.9	12.1	6.9	12.5	10.7
24	14.7	15.1	14.5	16.7	65.8	16.5	16.6	17.9	20.6	194.9
25	17.3	18.2	17.8	13.5	18.4	18.3	18.3	14.8	72.6	18.1
26	20.3	19.2	18.5	19.9	18.9	20.0	20.1	33.6	20.2	15.1
27	16.2	19.4	20.1	19.5	20.8	18.8	18.7	21.4	18.5	17.9
28	27.4	32.1	32.0	32.0	33.6	66.7	32.1	18.6	32.1	31.4
29	31.1	31.8	31.7	35.0	35.6	73.8	35.0	25.1	35.0	
30	35.2	34.6	34.9	31.7	32.7	26.5	31.8	28.9	31.6	107.6
Ref	[57]	[57]	[57]	[87]	[88]	[69]	[89]	[90]	[47]	[91]
**C**	**F97^C^**	**F98**	**F99**	**F100**	**F101**	**F102**	**F103**			
1	26.9	22.0	28.7	19.1	21.3	21.1	116.0			
2	105.0	41.2	71.5	34.5	38.0	37.2	166.1			
3	77.1	212.8	200.1	175.6	176.2	177.8				
4	47.7	57.9	127.7	36.1	35.8	36.2	197.9			
5	44.6	41.9	159.2	36.8	37.5	37.9	135.5			
6	30.1	41.0	30.9	38.8	38.7	39.0	136.0			
7	18.6	18.0	20.8	18.0	17.9	18.2	118.6			
8	48.1	53.1	47.7	52.6	52.1	53.1	163.3			
9	37.5	37.2	37.6	42.9	38.8	39.1	39.2			
10	52.1	59.2	52.3	58.2	59.6	59.9	165.9			
11	33.3	35.3	33.1	84.1	34.7	35.3	31.2			
12	28.8	29.8	29.5	37.6	29.5	30.3	29.2			
13	40.2	38.0	39.5	40.7	39.0	39.7	38.3			
14	39.1	39.6	40.0	37.9	37.9	38.4	47.6			
15	28.3	32.3	28.1	32.1	31.1	32.4	28.5			
16	29.6	35.5	29.7	35.9	29.0	36.1	36.6			
17	44.8	30.1	44.9	30.0	35.0	30.1	30.4			
18	45.6	41.6	45.3	42.6	39.3	43.0	44.6			
19	31.7	30.3	31.7	35.3	34.3	35.4	31.1			
20	41.5	31.4	41.3	28.1	27.8	28.2	40.4			
21	213.9	27.7	214.0	32.7	33.2	32.9	29.7			
22	77.4	38.8	77.2	39.2	31.3	39.3	34.4			
23	16.1	6.6	11.2	7.7	7.3	7.6	18.1			
24	175.3	14.4		22.1	19.2	19.5				
25	16.6	17.6	15.6	13.6	17.8	18.0	25.6			
26	15.8	18.2	19.1	19.9	18.6	20.2	24.0			
27	19.3	20.7	17.5	19.3	19.0	18.7	20.3			
28	26.1	31.8	25.2	32.0	67.3	32.2	31.5			
29		74.3		34.9	32.6	35.0	179.1			
30	15.6	25.9	14.8	31.7	33.9	31.9	32.8			
C=O		161.2								
OCH_3_							51.8			
OCH_3_							51.8			
Ref	[62]	[92]	[93]	[94]	[95]	[95]	[96]			

Ref: References; ^+ 13^C-NMR data of acetylated compound; * Values bearing the same superscript are interchangeable; Solvent: CDCl_3_; ^a^ CDCl_3_ + Pyridine-d_5_, ^b^ Pyridine-d_5_; ^c^ DMSO-d_6_; ^13^C-NMR data of some compounds were not found. In these cases, the reported identification was performed by comparison of other physical data: F5 [97], F33 [98], F46 (^1^H-NMR) [99], F48 (^1^H-NMR) [100], F58 (IR, MS) [101] and F78 (X-ray) [102].

**Table 2:** ^13^C-NMR data of quinonemethide-type pentacyclic triterpenoids isolated from Celastraceae species (2001–2021).

C	Q1	Q2	Q3	Q4	Q5	Q6 ^b^	Q7	Q8	Q9	Q10
1	121.6	119.8	119.8	164.7	119.5	119.7	119.8	120.0	119.5	119.8
2	178.8	178.4	178.3	178.3	178.1	178.4	178.4	178.1	178.3	178.4
3	145.9	146.0	146.2	146.0	145.9	146.1	146.2	146.3	146.0	146.1
4	118.0	117.0	117.5	118.2	118.0	117.2	117.1	116.8	117.2	117.2
5	128.6	127.5	128.5	127.5	127.3	127.9	127.9	127.9	127.3	127.8
6	132.2	133.8	132.1	134.0	134.0	133.3	133.3	134.4	134.0	133.6
7	118.8	117.9	118.6	117.2	118.0	118.3	118.3	121.9	118.0	118.2
8	167.2	165.7	164.9	169.7	170.1	168.7	168.7	18.6	169.7	168.4
9	48.1	43.0	43.0	47.8	43.0	42.9	42.9	44.6	42.4	42.6
10	161.6	164.4	164.2	164.0	164.8	164.2	164.2	159.7	164.6	164.7
11	65.3	34.3	32.5	33.7	33.0	33.2	33.2	37.3	33.5	34.0
12	43.4	27.5	31.1	30.2	30.0	29.9	29.9	35.9	29.5	30.0
13	40.6	40.3	39.0	42.6	40.7	40.0	40.0	42.4	39.2	40.6
14	44.9	47.3	49.6	44.6	45.0	44.2	44.2	136.9	44.5	44.3
15	28.7	129.4	73.0	28.4	28.6	29.4	29.4	128.6	28.2	28.3
16	36.2	135.6	41.4	68.2	36.5 *	35.7	35.7	40.5	36.1	29.5
17	30.87	33.7	37.6	30.2	31.6	35.9	35.9	39.8	38.1	44.8
18	43.9	42.6	43.7	46.9	43.2	43.3	43.3	43.7	46.1	45.1
19	30.81	30.7	31.9	29.6	24.8 **	36.9	36.9	40.7	30.8	32.0
20	40.4	41.0	41.8	39.4	35.7	73.7	73.7	75.0	42.6	40.9
21	29.7	29.4	213.7	28.4	24.8 **	214.9	214.9	213.0	38.1	213.5
22	34.3	32.3	54.2	38.3	36.5 *	50.5	50.5	50.5	67.9	76.4
23	10.4	10.3	10.3	10.3	10.4	10.3	10.3	10.4	10.1	10.3
24										
25	34.4	37.4	41.0	38.3	38.9	38.5	38.5	29.6	38.1	39.2
26	21.4	28.8	23.6	21.7	21.6	23.3	23.3	22.1	21.6	21.6
27	18.5	18.0	23.5	19.7	21.4	19.4	19.4	23.7	19.0	20.5
28	31.4	27.4	32.9	24.2	31.4	33.2	33.2	30.4	24.0	25.0
29	178.7	178.3		178.2		29.0			178.2	
30	32.7	31.3	15.0	32.4	69.6		29.0	24.8	32.2	14.7
OCH_3_	51.8	51.6		51.8					51.6	
Ref	[103]	[104]	[104]	[105]	[106]	[107]	[108]	[104]	[109]	[63]
**C**	**Q11**	**Q12**	**Q13**	**Q14**	**Q15**	**Q16**	**Q17**	**Q18**	**Q19**	**Q20 ^c^**
1	119.4	120.6	119.7	120.0	119.6	119.4	119.9	120.0	119.4	119.8
2	178.4	178.3	181.1	178.1	178.3	178.4	178.0	178.1	178.8	178.6
3	146.0	147.0	161.9	146.3	146.0	146.0	146.2	146.2	146.1	146.3
4	117.1	127.5	140.9	116.8	117.1	117.2	116.7	116.7	118.6	117.4
5	127.5	120.4	117.2	128.1	127.4	127.4	127.5	127.7	127.6	127.6
6	133.9	135.4	131.7	134.4	133.9	134.2	134.9	134.5	137.8	134.4
7	118.2	118.3	200.4	122.1	117.9	118.2	121.6	121.5	122.2	118.3
8	169.8	165.3	41.9	158.6	170.1	170.7	159.7	159.0	160.2	170.4
9	42.9	39.3	58.4	44.2	42.9	43.3	44.5	44.5	45.5	43.0
10	164.7	172.6	146.6	159.7	165.0	164.4	159.7	159.9	163.5	165.1
11	33.6	28.7	32.1	36.0	33.9	33.1	37.5	37.4	37.3	33.6
12	29.8	29.3	30.2	31.8	29.7	29.4	35.6	34.8	35.2	29.7
13	39.4	39.9	39.6	42.1	41.3	40.0	43.1	42.4	43.3	39.5
14	45.0	43.1	29.2	135.6	44.8	44.0	135.3	136.0	135.5	45.1
15	28.6	29.5	27.9	127.9	28.4	29.7	128.3	126.6	129.8	28.7
16	36.4	32.4	35.9	45.6	36.0	36.5	37.8	37.9	39.0	36.4
17	30.8	45.3	31.0	36.3	31.6	30.2	33.7	38.9	35.6	30.9
18	43.6	44.2	44.2	47.8	44.9	43.7	43.9	38.7	43.6	44.4
19	25.5	33.8	28.8	37.8	30.4	25.2	33.9	33.9	34.2	30.9
20	46.2	31.0	40.5	41.7	147.9	33.0	42.6	47.4	48.0	40.5
21	25.1	34.5	29.8	213.8	30.5	22.5	28.6	69.2	68.0	29.9
22	34.0	36.3	36.5	49.8	36.9	35.2	36.1	79.4	39.0	34.8
23	10.3	10.5	10.2	10.4	10.2	10.3	10.3	10.3	10.5	10.3
24										
25	38.4	38.4	38.2	28.5	38.9	37.7	29.4	29.5	29.4	38.3
26	21.7	21.5	21.5	21.3	21.3	23.4	21.9	21.8	21.9	21.7
27	18.4	18.7	18.3	23.1	19.7	17.9	24.0	24.5	24.3	18.4
28	31.5	31.5	31.5	30.2	31.1	36.2	31.5	27.0	31.1	31.7
29	177.4	182.5	178.8			69.3	179.3	179.0	178.8	179.1
30	74.1	30.7	30.8	16.0	108.2		19.8	13.7	17.5	32.7
OCH_3_	51.9		51.4				51.8	52.7	52.3	51.7
Ref	[91]	[110]	[111]	[112]	[113]	[91]	[104]	[109]	[109]	[114]
**C**	**Q21**	**Q22**								
1	119.8	119.8								
2	178.6	178.4								
3	146.3	146.0								
4	117.4	117.1								
5	127.8	127.7								
6	134.1	133.6								
7	118.3	118.1								
8	170.3	168.7								
9	43.2	42.7								
10	165.1	164.7								
11	34.0	33.8								
12	30.2	29.9								
13	40.7	40.6								
14	45.2	44.6								
15	28.9	28.5								
16	37.4	35.5								
17	30.6	38.2								
18	44.3	43.5								
19	25.0	32.0								
20	31.4	41.8								
21	71.2	213.6								
22	44.4	52.5								
23	10.4	10.2								
24										
25	38.9	39.0								
26	21.8	21.5								
27	21.4	19.7								
28	35.4	32.5								
29										
30	18.6	15.1								
Ref	[115]	[116]								

Ref: References; *^,^**: Values bearing the same superscript are interchangeable; Solvent: CDCl_3_; ^b^ C_6_D_6_:CDCl_3_; ^c^ CD_2_Cl_2._

**Table 3:** ^13^C-NMR data of aromatic-type pentacyclic triterpenoids isolated from Celastraceae species (2001–2021).

C	A1	A3	A5	A6	A7	A8 ^+^	A9 ^a^	A10 ^b^	A11	A12 ^c^
1	109.5	109.1	108.4	108.6	125.6	105.9	110.0	108.4	106.8	109.0
2	150.8	144.2	140.9	148.0	147.7	156.0	144.0	148.8	150.0	151.7
3	152.8	132.9	139.7	140.6	140.3	146.2	126.8	141.4	143.0	143.5
4	113.2	132.0	122.0	125.4	125.1	134.1	122.7	126.1	128.0	127.9
5	141.9	154.9	126.6	122.5	122.6	123.7	151.4	122.3	119.0	119.9
6	201.2	187.2	28.3	187.6	187.7	187.7	187.3	187.9	201.2	182.8
7	43.8	126.1	18.5	126.1	108.8	126.1	126.8	126.0	74.5	147.4
8	35.4	151.9	44.1	171.2	172.1	171.4	150.7	170.8	50.3	139.1
9	38.6	40.4	36.8	40.1	44.4	40.6	40.6	40.3	38.11	40.7
10	123.5	171.2	143.8	151.2	151.9	154.9	170.7	151.2	152.8	152.8
11	32.4	34.0	34.1	33.8	34.4	35.6	34.6	35.6	36.3	34.2
12	29.9	29.8	30.2	30.0	29.9	30.2	30.2	30.3	30.6	30.5
13	36.8	38.9	38.9	39.6	40.8	40.6	39.3	40.0	40.5	40.0
14	35.9	44.7	39.4	43.8	40.1	44.8	44.6	44.3	39.1	47.2
15	30.3	28.5	29.0	29.0	28.2	28.8	28.9	28.4	29.7	29.6
16	NR	36.4	36.5	35.5	36.8 **	37.5	36.6	32.1	36.2	37.8
17	35.7	30.8	30.3	36.2	31.6	30.8	30.6	38.4	29.8	31.1
18	40.4	44.3	44.5	43.3	44.8	42.7	44.4	43.5	44.1	45.4
19	29.5	30.5	30.6	36.8	30.5 *	27.9	31.1	34.3	29.8	31.9
20	39.1	40.3	40.6	73.8	148.2	132.4	44.4	42.0	41.0	41.7
21	29.6	29.7	30.0	215.0	30.5 *	119.9	30.1	214.7	29.9	30.9
22	28.2	34.8	36.2	50.2	36.8 **	35.2	35.1	52.7	34.0	36.2
23		14.7	11.3	13.6	13.6	14.4	14.8	13.7	14.0	14.0
24										
25	31.9	37.6	27.4	37.9	38.4	39.2	37.8	38.6	27.5	41.6
26	25.3	20.8	15.9	22.1	20.4	21.4	20.9	20.8	15.9	19.6
27	16.7	18.3	17.3	19.4	19.6	15.7	18.5	19.7	15.9	20.6
28	15.2	31.6	31.8	33.1	31.1	33.1	31.6	32.6	31.7	32.1
29	179.4	178.8	179.3	28.9	106.5	24.0	178.7		179.8	180.7
30	31.5	32.7	31.9				32.6	15.1	31.9	32.9
OCH_3_	55.7	51.5				56.0				
OCH_3_	55.1	61.1	51.5			60.7	51.5		51.4	52.2
Ref	[117]	[118]	[119]	[120]	[106]	[121]	[122]	[123]	[124]	[125]
**C**	**A13**	**A14**	**A15**	**A16**	**A17 ^+^**	**A18**	**A19 ^d^**	**A20**	**A21**	**A22**
1	107.9	107.5	109.2	107.9	104.1	110.1	116.6	112.4	107.8	108.8
2	150.0	148.4	146.6	150.0	156.3	151.7	150.7	152.6 *	141.7	148.2
3	141.4	140.9	139.5	141.4	146.1	144.7	150.0	143.5	140.4	140.7
4	129.1	127.0	130.0	129.1	135.1	134.0	117.0	120.4	122.0	125.6
5	122.8	124.5	125.0	122.8	125.1	122.4	122.3	121.8	123.9	122.5
6	182.0	201.9	200.7	182.0	201.0	185.8	186.3	185.8	43.9	187.7
7	197.0	37.9	37.7	197.0	37.7	129.3	125.2	124.8	209.8	125.9
8	60.3	42.9	42.6	60.3	42.5	160.1	174.4	177.4 **	58.3	171.3
9	38.7	37.6	37.2	38.7	38.0	42.2	40.5	41.7	38.8	40.3
10	153.6	153.2	152.2	153.6	156.2	151.2	150.4	151.7 *	142.4	151.7
11	33.5	36.6	33.2	33.5	33.8	37.1	33.7	35.0	33.7	34.3
12	27.8	30.15	29.9	27.8	29.6	35.8	29.6	30.8	29.4	30.2
13	39.5	39.7	38.8	39.5	40.2	40.7	39.4	40.7	39.1	40.1
14	39.2	39.2	39.4	39.2	40.3	134.7	45.1	46.4	38.7	44.4
15	38.7	28.8	28.5	38.7	28.2	125.9	28.7	30.0	28.4	28.4
16	35.8	36.4	36.2	35.8	38.5	38.0	26.4	37.6	36.1	35.5
17	30.2	30.7	30.3	30.2	30.7	48.8	30.5	31.6	30.3	38.2
18	43.6	45.1	44.7	43.6	42.9	46.2	44.3	45.7	43.6	43.5
19	30.6	31.1	30.5	30.6	27.6	34.2	30.8	31.9	30.6	32.0
20	40.7	41.1	40.1	40.7	133.0	50.3	40.1	41.3	40.6	41.9
21	30.3	30.1	29.8	30.3	120.2	74.3	29.8	30.9	29.8	214.0
22	35.8	33.5	36.1	35.8	35.4	214.0	34.8	36.0	35.9	52.6
23	13.8	14.0	13.2		14.3	15.1	200.2	173.1 **	11.5	13.7
24										
25	31.5	26.0	25.6	31.5	27.0	21.6	36.3	37.4	27.9	38.5
26	14.7	15.7	15.4	14.7	15.4	21.7	20.5	21.3	15.1	20.7
27	16.9	15.7	16.9	16.9	15.4	23.9	18.7	19.4	16.8	19.7
28	31.5	32.1	31.7	31.5	33.0	22.5	31.6	32.1	31.5	32.6
29	180.0	180.1	179.1	180.0	24.0	175.2	181.2	182.5 **	179.4	
30	32.5	32.9	32.2	32.5		13.7	32.6	33.2	32.3	15.1
OCH_3_					56.0	52.7				
OCH_3_	51.8	52.2	51.4	51.8	60.6	61.7			51.6	
1′			126.5							170.0
2′			104.2							121.3
3′			147.4							112.2
4′			135.8							146.2
5′			147.4							150.8
6′			104.2							125.2
7′			77.2							114.2
8′			75.5							
9′			62.8							
OCH_3_			56.4							56.1
OCH_3_			51.4							
OCH_3_			20.6							
C=O			170.3							
Ref	[119]	[124]	[126]	[119]	[124]	[109]	[127]	[127]	[119]	[128]
**C**	**A23**	**A24**	**A25 ^a^**	**A26 ^a^**	**A27 ^d^**	**A28**	**A29**			
1	108.3	107.7	110.7	110.6	108.5	116.5	113.8			
2	141.4	141.6	143.4	141.3	142.9	150.5	173.7			
3	139.8	139.5	145.2	143.1	141.9	149.7	155.5			
4	122.5	120.9	121.4	122.5	119.6	116.3	111.3			
5	126.3	125.7	126.7	126.0	122.8	117.1	119.4			
6	28.0	27.8	45.4	46.1	120.9	186.0	188.0			
7	18.3	126.0	119.6	120.9	137.3	122.8	124.4			
8	43.3	139.0	151.1	NR	43.3	149.1	153.4			
9	36.7	36.2	38.3	38.1	144.1	45.3	45.6			
10	143.8	140.0	142.6	145.1	128.5	125.3	152.8			
11	34.2	33.0	37.6	37.7	119.4	36.4	36.6			
12	30.0	29.4	31.4	30.6	32.6	28.6	28.6			
13	40.0	43.5	38.8	38.7	39.4	39.7	39.7			
14	39.3	58.0	44.7	44.5	40.1	40.5	40.5			
15	27.9	211.4	29.9	29.7	23.3	31.0	30.9			
16	29.6	47.5	37.6	37.4	36.7	34.8	34.8			
17	44.9	49.4	31.1	30.9	31.1	44.1	43.0			
18	45.4	44.3	45.1	44.9	46.3	44.1	44.2			
19	31.7	30.7	31.3	31.1	30.7	29.8	29.8			
20	41.3	40.0	40.8	40.6	38.3	30.7	30.5			
21	214.2	212.4	31.4	31.2	29.2	29.8	29.8			
22	77.6	77.8	35.9	35.7	37.2	33.5	34.4			
23	11.5	11.6	13.4	13.8	11.1	200.3	178.7			
24										
25	28.2	33.4	36.5	35.7	22.4	36.4	36.8			
26	15.3	25.6	22.7	22.4	19.1	20.6	20.2			
27	19.2	21.4	18.9	18.8	18.7	32.8	32.7			
28	25.2	24.6	32.0	31.9	32.7	18.3	18.3			
29			181.3	181.0	179.7	178.9	179.8			
30	14.8	14.8	33.5	33.3	30.4	31.8	31.6			
CH_3_			22.7	21.4		51.6	51.6			
CHOH			70.8	72.1						
Ref	[129]	[39]	[130]	[130]	[127]	[131]	[131]			

Ref: References; ^+ 13^C-NMR data of methylated compound; *^,^**: Values bearing the same superscript are interchangeable; NR: Not reported; Solvent: CDCl_3_; ^a^ Pyridine-*d_5_,* ^b^ CDCl_3_+CD_3_OD, ^c^ CD_3_OD; ^d^ DMSO-*d_6_*; ^13^C-NMR data for A2 were not found. The reported identification was performed by comparison of ^1^H-NMR data from Sotanaphun [132]. ^13^C-NMR data for A4 were reported by Shirota et al. [122], but the chemical shifts were not attributed to each carbon atom.

**Table 4:** ^13^C-NMR data of dimer-type pentacyclic triterpenoids isolated from Celastraceae species (2001–2021).

C	D1	D2	D3	D6	D7	D8	D9	D10	D11	D12
1	115.5	108.2	115.5	113.0	113.0	113.1	113.0	113.1	115.5	115.0
2	191.2	144.3	191.0	191.5	191.5	191.5	191.6	191.4	190.2	189.4
3	91.7	140.6	91.8	91.3	91.3	91.2	91.3	91.3	92.0	91.1
4	79.9	123.2	79.0	79.5	79.5	79.5	79.4	79.5	79.4	76.9
5	130.8	124.6	130.8	134.1	134.3	134.1	134.3	134.1	130.2	132.2
6	126.1	71.4	125.9	134.1	133.9	133.9	134.1	133.8	126.6	128.5
7	116.2	73.7	116.1	24.2	24.2	24.2	24.2	24.2	116.3	117.2
8	160.6	44.7	160.6	41.6	41.2	41.6	41.1	41.6	160.4	163.3
9	41.7	40.2	41.7	37.4	37.4	37.4	37.3	37.4	41.7	43.4
10	173.8	143.7	173.5	170.2	169.8	170.0	169.8	170.0	173.8	172.9
11	32.7	34.7	32.7	30.7	30.5	30.6	30.7	32.0	33.2	33.1
12	29.5	30.7	29.5	29.4	29.4	29.4	29.7	29.4	29.8 ^1^	29.8
13	38.1	39.6	38.1	38.9	40.1^1^	38.9	40.0 ^1^	40.1	39.5 ^2^	39.9
14	44.5	40.00	44.5	40.1	40.2^1^	40.1	39.9 ^1^	38.9	44.3	44.0
15	28.4	31.4	28.4	28.3	27.9	28.4^1^	27.7	28.3	28.3	28.5
16	36.5	36.1	36.4	36.0	35.3	36.0	29.3	36.0	35.4 ^3^	35.4
17	30.5	29.8	30.2	30.2	38.1	30.2	44.7 ^2^	30.1	38.3 ^4^	38.2
18	44.6	43.6	44.1	44.6	43.9	44.6	45.4	44.6	43.4	43.6
19	30.9	29.9	30.9	30.5	31.8	30.5	31.7	30.5	32.1	31.9
20	40.5	40.6	40.5	40.5	42.3	40.5	41.2	40.5	42.3	41.9
21	29.9	30.7	29.9	29.9	213.8	29.9	213.8	29.7	213.6	213.6
22	34.8	37.0	34.7	36.0	53.5	36.0	77.2	36.0	52.5	52.4
23	22.4	11.1	22.5	22.7	22.7	22.7	22.8	22.7	22.3	24.6
24										
25	34.8	28.0	34.8	22.1	22.9	22.1	23.0	22.1	35.6	39.7
26	22.5	17.6	22.5	16.0	15.7	16.0	15.8	16.0	22.3	22.4
27	18.6	18.3	18.6	16.9	18.1	16.9	18.9	16.8	20.1	19.7
28	31.6	31.9	31.7	31.7	32.7	31.7	25.1	31.7	32.8 ^5^	32.5
29	179.1^1^	179.4	179.0 ^1^	179.0		179.0		179.0		
30	32.9	31.7	31.9	32.3	15.2	32.3	14.9	32.3	15.2 ^6^	15.1
OCH_3_	51.7^2^	51.3	51.5 ^2^	51.7		51.7		51.7		
1′	110.4	109.5	110.4	110.5	110.6	110.4	110.6	110.4	109.6	108.8
2′	139.1	140.1	141.3	144.5	144.5	144.5	144.5	144.5	145.0	145.3
3′	136.2	138.9	137.0	138.3	138.3	138.4	138.2	138.4	137.6	137.4
4′	122.9	125.6	125.5	129.4	129.4	129.5	129.3	129.6	129.0	129.7
5′	127.9	123.6	127.2	123.3	123.3	123.4	123.7	123.4	126.0	125.3
6′	26.4	28.0	75.2	187.2	187.2	187.1	187.2	187.0	201.1	200.0
7′	18.5	18.5	21.8	126.3	126.3	126.3	126.3	126.3	37.6	37.4
8′	43.9	44.1	38.5	171.0	171.0	170.0	171.1	169.7	41.9	41.8
9′	36.8	36.9	37.6	40.1	40.1 ^1^	39.9	40.1	39.7	37.1	37.0
10′	144.4	145.1	144.7	151.8	151.8	151.7	151.8	151.7	151.7	152.2
11′	33.9	34.3	33.8	34.2	34.3	34.4	34.3	34.6	32.9	33.2
12′	30.0	30.3	29.8	29.7	29.9	30.2	29.9	29.8	29.7 ^1^	29.5
13′	38.9	39.0	38.9	39.0	39.0	40.2	39.0	40.1	39.4 ^2^	39.2
14′	39.4	39.4	39.1	44.7	44.7	44.3	44.8 ^2^	44.0	40.0	39.8
15′	28.9	29.1	29.0	28.5	28.6	28.3 ^1^	28.6	28.2	28.0	27.7
16′	36.4	36.3	36.1	36.4	36.4	35.5	36.4	29.6	35.3 ^3^	29.3
17′	30.6	30.2	29.3	30.5	30.5	38.2	30.5	44.9^1^	38.2 ^4^	45.0
18′	44.1	44.4	44.4	44.3	44.3	43.5	44.3	45.0^1^	44.0	45.3
19′	30.4	29.5	30.5	31.0	31.0	32.0	31.1	30.1	31.8	31.7
20′	40.4	40.6	40.4	40.6	40.6	41.9	40.6	40.9	41.9	41.3
21′	30.3	30.7	30.3	29.9	29.7	213.7	29.7	213.6	214.1	214.0
22′	36.3	36.6	36.6	35.0	35.0	52.7	35.0	76.1	53.6	77.2
23′	10.9	10.9	10.6	13.4	13.4	13.4	13.4	13.4	13.0	13.3
24′										
25′	27.2	27.2	31.5	37.7	37.7	38.7	37.6	38.9	26.2	26.5
26′	15.8	16.1	16.3	20.9	20.9	20.8	20.8	20.9	15.0	15.1
27′	17.2	17.3	17.4	18.6	18.5	20.0	18.6	20.8	18.2	19.0
28′	31.8	31.9	26.4	31.6	31.6	32.6	31.6	25.0	32.6 ^5^	25.1
29′	178.7 ^1^	179.8	178.7 ^1^	179.2	179.3		179.4			
30′	32.7	30.8	32.7	32.9	32.9	15.1	33.0	14.8	15.1 ^6^	14.8
OCH_3_	51.5 ^2^	51.6	51.7 ^2^	51.6	51.6		51.7			
OCH_3_			55.4							
Ref	[133]	[134]	[133]	[135]	[136]	[136]	[137]	[137]	[136]	[136]
**C**	**D13**	**D14**	**D15**	**D16**	**D17**	**D18**	**D19**	**D20**	**D21**	**D22**
1	114.0	108.3	49.1	108.6	107.8	107.6	109.1	108.8	111.4	108.9
2	191.2	144.4	193.1	146.5	142.0	141.4	144.9	141.1	149.6	137.9
3	91.3	140.7	192.1	141.7	137.7	141.4	142.7	141.7	143.2	142.2
4	79.1	123.2	60.5	123.0	121.4	120.8	122.3	121.3	126.0	121.1
5	134.7	124.7	131.7	125.4	124.3	124.4	125.7	126.0	124.1	125.0
6	135.9	71.5	28.2	124.3	124.4	123.6	124.3	124.2	187.7	124.3
7	68.5	74.5	18.3	128.6	128.4	127.7	128.7	129.2	126.1	128.8
8	51.7	45.1	45.7	45.4	45.6	44.8	44.4	45.6	171.2	45.5
9	41.1	40.3	37.4	37.5	37.2	36.3	37.2	37.2	40.05	37.0
10	168.7	143.7	148.8	143.6	142.2	141.3	143.3	142.8	151.7	141.8
11	31.2	34.8	31.0	31.0	31.1	30.4	30.5	31.2	34.12	31.9
12	29.4	30.4	29.7	30.0	30.4	29.2	38.2	30.5	28.5	30.5
13	39.4	39.7	39.6	38.9	39.3	38.1	38.8	38.9	39.0	38.8
14	41.8	40.0	39.3	39.0	39.2	38.2	45.4	39.0	44.6	39.0
15	31.0	31.7	28.9	28.1	28.1	27.3	28.1	28.2	29.8	28.1
16	36.2	36.2	36.4	35.5	36.0	35.6	35.4	36.0	35.4	36.2
17	30.0	29.8	30.3	30.3	30.6	29.6	30.3	30.4	30.5	30.4
18	44.8	43.6	44.5	44.4	44.5	43.7	43.6	44.5	44.3	44.4
19	30.6	30.0	30.4	30.6	30.0	29.2	30.0	30.1	30.8	31.0
20	40.6^1^	40.7	40.7	40.6	40.5	39.7	40.5	40.5	40.4	40.5
21	29.8	31.6	30.0	29.7	29.9	29.8	29.9	29.9	29.9	29.4
22	35.8	37.1	36.7	36.5	36.4	36.1	35.9	36.4	36.4	36.3
23	22.4	11.1	9.0	10.8	10.8	9.9	10.7	10.7	13.1	10.9
24										
25	24.0	28.0	22.3	22.2	16.8	21.6	22.1	22.3	37.7	22.3
26	16.5	17.8	16.0	17.0	22.2	16.0	16.8	16.9	20.8	16.8
27	17.4	18.3	17.2	17.5	17.4	16.6	17.3	17.4	18.3	17.4
28	31.7	32.00	31.9	31.8	31.8	31.0	31.7	31.8	31.6	31.8
29	179.0	179.4	179.2	179.3	179.1	178.3	179.1	179.1	178.8	179.1
30	32.4	32.00	31.9	32.2	32.1	31.3	32.1	32.1	32.7	32.1
OCH_3_	51.7	51.6	51.6	51.6	51.5	50.7	51.6	51.5	51.5	51.5
1′	110.6	107.9	60.8	92.7	87.1	90.3	92.2	90.5	92.7	139.9
2′	144.2	141.6	24.4	38.6	41.8	44.6	36.3	45.8	38.4	36.0
3′	138.0	139.1	41.3	128.7	124.2	124.4	128.2	128.2	128.1	74.2
4′	129.3	122.0	39.8	140.5	151.2	141.2	140.6	140.6	140.6	90.1
5′	123.5	124.1	44.4	97.1	84.3	89.4	96.8	89.7	96.8	134.6
6′	187.2	124.3	25.1	38.1	35.2	35.2	38.9	84.6	39.1	30.0
7′	126.2	128.6	36.1	43.7	45.3	39.9	45.5	41.4	43.3	49.4
8′	171.0	45.8	151.4	36.0	38.9	35.8	32.7	29.7	34.8	29.9
9′	40.2	37.5	42.5	32.7	39.0	29.7	31.1	29.6	33.0	39.8
10′	151.9	143.8	36.2	40.1	32.9	43.0	39.4	43.2	39.3	42.6
11′	34.2	31.7	34.8	151.0	151.9	150.6	151.1	140.1	150.8	150.7
12′	29.9	30.0	21.9	108.7	108.4	108.0	108.6	119.1	108.8	108.6
13′	39.0	38.9	30.0	19.9	19.4	20.1	19.9	169.7	20.0	20.5
14′	44.7	39.1	16.7	19.1	20.3	17.8	19.1	19.0	19.1	17.1
15′	28.6	30.4	111.8	12.6	22.9	10.7	12.5	14.4	12.4	13.9
16′	36.4	36.1								
17′	30.5	30.5								
18′	44.3	44.5								
19′	30.9	29.4								
20′	40.5 ^1^	40.6								
21′	29.8	31.6								
22′	35.0	36.6								
23′	13.4	10.8								
24′										
25′	37.6	22.3								
26′	20.9	17.2								
27′	18.4	17.3								
28′	31.6	31.8								
29′	179.0	179.6								
30′	32.8	31.6								
OCH_3_	51.7	51.4								
Ref	[136]	[134]	[75]	[138]	[138]	[138]	[138]	[138]	[138]	[138]
**C**	**D23**	**D24**	**D25**	**D26**	**D27**	**D28**	**D30**	**D31**	**D32**	**D33**
1	107.6	108.2	116.0	114.8	116.5	114.6	115.4	108.2	115.9	114.9
2	145.8	142.4	187.8	187.3	193.3	189.6	191.0	144.4	190.8	189.6
3	136.2	138.6	92.2	90.8	93.1	91.3	91.8	140.6	92.0	90.7
4	122.7	121.6	79.1	76.9	79.2	77.2	79.1	123.0	79.4	77.2 ^11^
5	124.0	124.6	130.3	131.7	130.7	131.3	130.6	124.7	130.5	132.1
6	124.2	124.2	126.6	128.8	126.7	129.8	126.1	71.3	125.9	128.7
7	128.3	128.6	116.0	117.3	115.7	117.2	116.2	74.2	116.2	116.9
8	45.6	45.6	161.8	164.4	162.4	164.8	160.8	45.0	161.2	164.5
9	37.3	37.3	41.9	43.8	42.0	43.9	41.8	40.3	41.6	44.1
10	143.0	142.7	174.4	173.4	174.8	173.8	173.8	143.9	173.4	173.3
11	31.1	31.1	32.8	32.9	33.4	32.88	32.8	34.6	32.8	32.9
12	30.1	30.0	29.2	29.4	29.3	29.3	29.5	30.3	29.2	29.9 ^1^
13	39.0	38.9	38.1	38.9	38.0	38.5	38.2	39.6	37.9	38.7
14	38.9	39.0	44.6	44.3	44.6	44.4	44.5	39.9	44.6	44.4
15	28.2	28.2	28.3	28.6	28.4	28.6	28.3	28.5	28.5	28.6 ^2^
16	36.4	36.0	36.3	36.2	36.2	36.2	36.4	36.1	36.3	36.4
17	30.4	30.4	30.5	30.4	30.4	30.4	30.4	29.9	30.5	30.6
18	44.5	44.5	44.0	44.1	44.1	44.1	44.2	43.9	44.2	44.4 ^3^
19	30.6	30.6	30.9	30.6	31.0	30.7	30.9	30.7	30.6	30.9 ^4^
20	40.5	40.5	40.0	40.3	39.7	40.1	40.5	40.7	40.3	40.5 ^5^
21	30.0	29.8	29.6	29.8	29.7	29.4	29.8	29.7	29.4	29.9 ^6^
22	36.0	36.4	34.6	34.8	34.3	34.4	34.8	36.9	34.7	34.8 ^7^
23	11.0	10.9	22.2	24.5	22.2	24.4	22.0	11.1	22.4	24.2
24										
25	22.3	22.2	34.8	39.2	35.2	39.1	34.8	28.2	37.5	39.3
26	16.9	16.9	22.4	22.5	18.4	18.6	22.3	17.1	22.0	22.4
27	17.5	17.4	18.8	18.5	22.2	22.2	18.7	18.4	18.9	18.3 ^8^
28	31.8	31.8	31.6	31.5	31.4	31.4	31.6	31.8	31.6	31.6 ^9^
29	179.1	179.1	184.5	184.3	182.4	183.5	178.7	179.4	184.4	178.9 ^10^
30	32.1	32.1	32.6	32.4	31.8	32.4	32.7	31.3	32.7	32.8
OCH_3_	51.5	51.5					51.7	51.6		51.6
1′	135.4	142.7	111.2	110.8	36.2	37.9	108.8	108.2	110.4	110.7
2′	36.5	34.5	144.4	145.0	18.7	18.8	140.8	142.1	144.7	144.2
3′	74.2	78.8	137.5	137.4	40.4	41.3	136.5	138.0	138.2	138.5
4′	89.9	86.1	127.2	128.3	33.0	33.1	121.3	121.0	129.5	128.2
5′	137.7	138.0	124.4	123.6	49.8	49.2	126.0	125.7	23.0	124.0
6′	32.1	35.0	192.0	189.9	35.7	35.9	124.0	124.4	187.5	187.8
7′	49.6	46.3	126.0	126.1	197.1	198.4	129.4	128.9	126.0	126.2
8′	29.7	30.0	171.4	171.0	126.0	125.9	45.5	45.9	171.4	171.5
9′	39.6	38.5	40.3	40.0	150.3	151.3	37.4	37.6	40.0	39.9
10′	42.6	34.7	150.1	151.1	37.7	37.7	142.8	141.9	151.9	151.1
11′	150.7	151.2	34.0	33.8	112.6	112.1	30.6	31.1	34.2	34.2
12′	108.7	108.5	29.3	29.1	147.2	147.0	30.0	30.0	29.9	29.6 ^1^
13′	20.4	20.4	38.9	38.5	139.8	139.4	38.9	39.0	39.0	39.0
14′	17.1	19.3	44.7	44.6	114.9	115.8	39.0	39.1	44.6	44.7
15′	14.0	22.3	28.3	28.4	32.8	32.5	28.4	29.9	28.5	28.5 ^2^
16′			36.2	36.2	21.3	21.3	36.0	36.1	36.3	36.4
17′			30.4	30.3	23.3	23.2	30.6	30.5	30.4	30.6
18′			44.1	44.1			44.5	44.5	44.0	44.3 ^3^
19′			30.5	30.7			31.0	30.7	31.1	30.8 ^4^
20′			39.7	40.1			40.4	40.6	40.2	40.4 ^5^
21′			29.5	29.7			29.7	30.0	29.3	29.7 ^6^
22′			34.4	34.5			36.4	36.7	34.4	34.7 ^7^
23′			12.9	13.2			10.8	11.0	13.2	12.8
24′										
25′			36.8	37.5			22.5	22.2	34.7	38.0
26′			20.7	20.9			16.9	17.1	20.9	20.9
27′			18.6	18.8			17.4	17.6	18.8	18.2 ^8^
28′			31.3	31.5			31.8	31.8	32.5	31.6 ^9^
29′			183.6	184.3			178.7	179.5	183.6	178.7 ^10^
30′			32.4	32.2			32.1	32.1		32.8
OCH_3_							51.6	51.5	32.4	51.6
Ref	[138]	[138]	[139]	[139]	[139]	[139]	[133]	[134]	[139]	[136]
**C**	**D34**	**D35**	**D36**	**D37**	**D38**	**D39**	**D40**	**D41**	**D42**	**D43**
1	116.0	114.9	116.0	109.0	111.4	115.8	114.7	115.5	115.5	115.0
2	190.4	189.5	190.4	145.1	149.7	191.1	189.4	190.2	190.3	189.5
3	91.8	90.6	91.8	142.8	143.3	91.9	91.0	92.0	92.0	91.0
4	79.4	76.9	79.3	122.5	125.2	78.8	77.3	79.3	79.4	76.8
5	130.9	132.0	130.9	125.7	122.4	131.1	131.8	130.3	130.3	132.1
6	126.1	128.7	126.2	124.6	187.5	126.1	128.8	126.6	126.6	128.7
7	116.2	116.8	116.4	128.3	126.2	116.4	117.2	116.3	116.3	117.3
8	160.2	164.6	159.9	44.9	170.2	159.5	164.4	160.3	160.1	163.0
9	41.5	44.2	41.4	39.4	39.3	41.3	43.9	41.7	41.6	43.7 ^1^
10	173.2	173.4	173.2	143.3	151.7	173.4	173.2	173.7	173.8	173.0
11	33.3	32.8	33.5	31.7	34.3	33.5	32.8	33.2	33.4	33.3
12	29.8 ^1^	29.5	29.9 ^1^	29.9	30.2	30.0	29.8	29.9 ^1^	29.9 ^1^	29.9
13	39.5	38.6	39.5	38.3	39.9	39.5	38.6	39.4	39.4	39.8
14	44.2	44.4 ^1^	43.9	32.8	44.3	44.1	44.4	44.3	44.0	43.61
15	28.3	28.6	28.1	27.5	28.4	28.3	28.6	28.3	28.0	28.2
16	35.5	36.4	29.5 ^2^	35.3	35.6	29.7	36.4	35.4	29.5	29.5
17	38.2	30.5	44.7	35.4	38.2	39.0	30.5	38.2	44.7	45.0
18	43.4	44.1	44.9	43.5	43.5	43.8	44.2	43.4	44.9	44.8
19	32.2	30.8	32.1	31.0	32.0	32.1	30.9	32.1	32.1	31.9
20	41.9	40.4	40.8	40.1	41.9	41.8	40.5	41.9	40.8	40.9
21	213.6	29.9	213.5	214.3	213.7	213.6	29.8	213.6	213.6	213.5
22	52.5	34.7	76.5	53.9	52.6	52.5	35.0	52.5	76.5	76.4
23	22.1	24.2	22.2	10.8	13.2	22.1	24.6	22.3	22.2	24.6
24										
25	35.7	39.3	35.8	32.8	38.7	35.6	39.2	35.6	35.6	40.1
26	22.3	22.4	22.4	18.6	20.8	22.3	22.3	22.3	22.4	22.3
27	20.1	18.2	20.9	16.1	19.7	15.0	18.2	20.0	20.9	20.4
28	32.5	31.5	25.0	22.7	32.6	32.5	31.6	32.6	25.0	25.0
29		178.8					179.1			
30	15.1	32.7	14.7	15.1	15.1	20.0	32.9	15.1	14.8	14.8
OCH_3_		51.6					51.6			
1′	110.5	110.7	110.6	92.9	92.8	108.1	110.6	111.4	111.3	110.6
2′	144.4	144.3	144.4	38.4	38.8	141.6	145.2	144.6	144.6	145.1
3′	138.3	138.5	138.3	128.2	128.1	137.6	137.5	137.6	137.6	137.5
4′	129.3	128.3	129.3	140.6	140.7	122.5	128.3	127.6	127.7	128.4
5′	123.3	123.9	123.4	96.8	96.8	125.0	123.8	124.5	124.5	123.9
6′	187.2	187.6	187.2	38.2	38.4	124.0	187.4	187.9	187.8	187.3
7′	126.3	126.1	126.2	43.6	43.3	129.2	126.1	126.1	126.2	126.1
8′	171.0	170.4	171.1	35.7	35.4	45.5	171.2	171.7	171.6	171.7
9′	40.1	39.7	40.8	32.7	33.0	38.2	40.0	40.0	39.9	40.0
10′	151.8	151.0	151.8	42.2	39.3	143.8	151.1	150.5	150.5	151.2
11′	34.3	34.3	34.3	151.2	150.9	36.6	34.0	34.2	34.2	34.0
12′	30.0	30.2	29.8 ^1^	108.6	108.8	36.4	29.8	29.9 ^1^	29.9^1^	29.9
13′	39.0	40.2	39.0	19.9	20.0	37.5	39.0	39.0	39.0	39.0
14′	44.7	44.3^1^	44.9	19.0	19.1	38.2	44.7	44.7	44.9	44.7
15′	28.6	28.4	28.6	12.5	12.4	28.3	28.5	28.5	28.5	28.5
16′	36.4	35.5	36.4			35.4	36.4	36.4	36.4	36.4
17′	30.5	38.2	30.5			30.4	30.5	30.5	30.5	30.5
18′	44.3	43.5	44.3			44.4	44.2	44.3	44.3	44.2
19′	31.1	32.0	31.1			29.8	30.8	30.9	30.8	30.9
20′	40.6	41.9	40.7			40.6	40.4	40.4	40.4	40.5
21′	29.7 ^1^	214.7	29.7 ^2^			29.8	29.5	29.9 ^1^	29.8	29.7
22′	35.0	52.6	35.0			35.8	34.7	34.7	34.8	35.0
23′	13.3	12.8	13.3			10.8	13.2	13.0	13.0	13.2
24′										
25′	37.6	38.9	37.7			22.3	37.7	37.6	37.6	37.7
26′	20.9	20.8	20.8			17.5	20.9	20.8	20.8	20.9
27′	18.5	19.7	18.6			17.0	18.4	18.3	18.3	18.5
28′	31.6	32.6	31.6			31.8	31.5	31.6	31.6	31.6
29′	179.3		179.4			179.3	178.8	178.7	178.8	179.1
30′	32.9	15.1	33.0			31.8	32.7	32.7	32.7	32.9
OCH_3_	51.6		51.8			51.5	51.4	51.6	51.6	51.4
Ref	[136]	[136]	[137]	[128]	[128]	[128]	[140]	[136]	[137]	[137]
**C**	**D44**	**D45**	**D46**	**D47**	**D48**	**D49**	**D50**			
1	115.2	114.6	115.5	115.5	108.2	108.2	128.3			
2	190.2	189.4	190.2	189.5	144.5	144.5	183.6			
3	92.0	91.1	92.0	92.0	140.7	140.7	96.9			
4	79.4	77.2	79.4	79.4	122.8	122.9	92.3			
5	129.8	131.8	130.3	130.2	124.7	124.7	39.5			
6	126.8	128.9	126.6	126.5	71.4	71.4	28.1			
7	116.1	117.2	116.2	116.3	74.1	74.1	32.8			
8	161.5	164.4	160.4	160.5	43.6	43.6	30.4			
9	42.0	44.0	41.7	41.8	40.4 ^1^	40.4 ^1^	43.7			
10	174.3	173.2	173.7	173.6	143.7	143.7	140.7			
11	32.9	32.8	33.2	32.8	34.5	34.5	35.4			
12	29.5	29.6^1^	29.8	29.5	30.2	30.2	30.6			
13	38.1	38.6	39.4	38.5	40.6 ^1^	40.6 ^1^	44.5			
14	44.7	44.4	44.3	44.2	40.4 ^1^	40.4 ^1^	40.5			
15	28.4	28.6	28.3	28.4	29.3	29.3	30.0			
16	36.3	36.4	35.4	35.5	35.5	35.5	36.4			
17	30.5	30.5	38.2	37.1	37.9	37.9	40.5			
18	44.1	44.3	43.4	43.6	43.8	43.7	43.7			
19	30.9	30.8	32.1	34.2 ^1^	31.9	31.9	43.7			
20	40.4	40.4	41.9	53.6	42.2	42.2	151.2			
21	29.8	30.1 ^2^	213.6	209.4	214.7	214.6 ^2^	39.0			
22	34.7	34.7	52.5	51.9	53.9	53.9	38.2			
23	22.2	24.6	22.3	22.3	11.3	11.3	12.6			
24										
25	34.9	39.1	35.6	35.2	29.1	29.1				
26	22.5	22.4	22.3	22.8	15.7	15.7	19.1			
27	18.7	18.2	20.0	18.2	19.3	19.2	32.1			
28	31.6	31.6	32.6	32.6	33.1	33.1	20.5			
29	178.9	178.8		175.1			108.7			
30	32.7	32.8	15.1	25.1	15.2	15.2	19.9			
OCH_3_	51.6	51.6		52.6						
1′	111.4	110.5	111.4	111.4	109.7	109.7	109.2			
2′	144.7	145.2	144.7	144.6	141.5	141.4	145.0			
3′	137.7	137.6	137.7	137.6	140.3	140.3	142.8			
4′	127.8	128.6	127.8	127.8	121.8	121.8	122.4			
5′	124.5	123.9	124.5	124.5	124.3	124.3	125.7			
6′	187.7	187.2	187.7	187.7	119.7	119.8	124.4			
7′	126.1	126.2	126.2	126.1	138.2	138.1	128.7			
8′	170.4	169.9	170.8	170.7	43.9	43.9	45.5			
9′	39.7	39.7	39.8	39.8	142.8	143.0	37.3			
10′	150.4	151.1	150.3	150.4	131.8	131.8	143.3			
11′	34.5	34.3	34.1	34.3 ^1^	122.7	122.6	31.2			
12′	30.2	30.1 ^2^	29.9	30.1	37.5	37.4	30.0			
13′	40.1	40.1	39.2	40.2	40.1	40.1	38.9			
14′	44.0	43.9	44.3	44.3	40.7	40.4 ^1^	38.9			
15′	28.2	28.2	28.6	28.4	24.0	23.6	28.1			
16′	29.6	29.5 ^1^	35.7	35.5	35.9	29.5	36.4			
17′	44.9	44.9	37.1	38.2	39.2	45.3	30.4			
18′	45.0	45.0	43.9	43.5	45.7	46.7	44.5			
19′	32.0	31.9	34.2	32.0	32.5	32.1	30.6			
20′	40.9	40.9	53.6	41.9	42.4	41.3	40.5			
21′	213.7	213.6	209.4	213.7	214.9	214.5 ^2^	30.0			
22′	76.5	76.7	51.9	52.6	51.2	75.4	36.0			
23′	13.0	13.2	13.0	13.0	10.7	10.7	10.8			
24′										
25′	38.7	38.9	38.2	38.4	22.4	22.4	22.1			
26′	20.9	20.9	21.2	20.8	19.6	19.8	16.9			
27′	20.6	20.7	17.8	19.7	20.6	21.4	17.4			
28′	25.0	25.0	32.6	32.6	31.5	24.2	31.8			
29′			175.0				179.2			
30′	14.8	14.7	25.1	15.1	15.4	15.0	32.1			
OCH_3_			52.5				51.6			
Ref	[137]	[137]	[137]	[137]	[141]	[141]	[142]			

Ref: References; ^1,2,3,4,5,6,7,8,9,10^: Values bearing the same superscript are interchangeable; ^11^: Signal bearing this superscript was superimposed on solvent signals; Solvent: CDCl_3_; ^13^C-NMR data for D4, D5 and D29 were reported by Gonzalez et al. [143] but the chemical shifts were not attributed to each carbon atom.

**Table 5:** ^13^C-NMR data of lupane-type pentacyclic triterpenoids isolated from Celastraceae species (2001–2021).

C	L1 ^+^	L2	L3	L4	L5	L6	L7	L8	L9	L10
1	38.4	38.7	41.8	123.6	42.1	39.6	39.6	79.5	39.8	39.3
2	23.7	27.4	34.0	165.1	34.2	34.1	34.1	42.9	34.3	34.3
3	80.9	79.0	218.3	205.3	218.8	218.0	217.7	216.1	218.2	221.4
4	37.9	38.9	47.5	45.0	47.6	47.3	47.2	47.1	47.5	50.7
5	55.4	55.2	54.6	52.9	54.8	54.9	54.9	51.2	55.1	55.3
6	18.2	18.3	19.4	18.9	19.6	19.6	19.6	19.6	19.8	19.2
7	34.3	34.3	33.9	34.5	34.3	33.5	33.6	32.9	33.7	33.6
8	40.9	40.8	42.2	42.9	42.4	40.8	40.6	41.0	41.0	40.7
9	49.9	50.0	54.5	48.9	54.9	49.3	49.8	50.6	50.0	49.5
10	37.1	37.1	38.0	40.6	38.2	36.8	36.9	45.1	37.0	36.6
11	20.9	20.8	70.1	70.6	70.5	21.4	21.5	22.9	21.6	21.7
12	27.6 *	26.5	37.2	37.3	37.4	24.8	25.1	25.1	25.0	25.2
13	37.9	37.8	36.1	37.1	37.2	37.4	37.7	37.9	37.8	37.5
14	43.1	42.9	42.2	42.8	42.6	44.1	41.9	42.9	42.8	42.8
15	27.2	27.2	26.8	27.3	27.4	36.8	26.9	27.5	27.3	27.0
16	35.3	35.3	33.6	35.3	35.4	76.9	33.1	35.5	35.9	34.0
17	43.0	43.1	47.3	43.0	43.0	48.6	80.2	42.9	42.0	47.8
18	49.0	47.1	47.8	47.5	47.6	47.6	48.3	47.9 *	48.2	48.6
19	42.8	37.4	47.4	47.7	47.7	47.5	48.0	48.2 *	60.0	47.8
20	49.0	49.7	149.5	150.2	150.2	149.9	149.6	150.7	148.4	150.3
21	25.2 *	23.6	29.4	29.7	29.8	29.8	29.4	29.8	77.9	29.7
22	40.0	40.5	28.8	39.8	39.8	37.7	38.5	40.0	49.6	29.1
23	28.0	28.0	27.2	21.4	27.5	26.6	26.6	28.0	26.8	22.1
24	16.6	15.4	20.5	28.2	20.8	21.0	21.0	19.8	21.2	65.3
25	16.1 **	16.0	16.5	19.9	16.7	16.0	16.0	11.9	16.1	17.0
26	16.0 **	15.9	16.6	17.4	16.9	15.8	15.9	15.9	16.0	15.6
27	14.4	14.3	14.4	14.4	14.4	16.1	13.8	14.4	14.6	14.7
28	18.0	17.9	60.3	18.0	18.1	11.7		18.1	19.8	60.5
29	207.0	7.3	110.1	113.5	109.9	109.8	109.6	109.5	111.5	109.8
30	14.5	205.1	18.9	19.3	19.4	19.3	19.3	19.3	19.9	19.1
COOCH_3_	21.3									
COOCH_3_	171.0									
Ref	[144]	[145]	[146]	[147]	[148]	[149]	[145]	[150]	[14]	[151]
**C**	**L11**	**L12**	**L13**	**L14**	**L15**	**L16**	**L17 ^a^**	**L18**	**L20**	**L21**
1	39.6	39.6	125.2	39.8	40.2	39.7	34.1	42.4	39.7	53.5
2	34.1	34.1	159.2	34.0	35.7	34.3	26.6	34.3	34.2	211.4
3	218.2	218.0	205.0	218.0	216.5	218.3	75.1	218.6	217.9	82.5
4	47.3	47.4	44.6	47.3	47.7	47.5	38.1	47.6	47.4	45.6
5	54.9	55.0	53.5	54.8	55.4	55.1	49.2	54.9	55.1	54.6
6	19.6	19.6	19.0	19.0	20.4	19.8	18.5	19.5	19.7	18.5
7	33.5	33.5	33.7	33.5	34.2	33.8	34.5	34.2	33.7	34.0
8	40.9	42.7	41.8	40.9	41.5	41.0	41.1	42.1	40.8	41.3
9	49.7	49.6	44.5	49.7	49.8	50.0	50.8	54.7	49.7	50.4
10	36.8	36.9	39.5	36.8	37.7	37.0	37.7	38.2	36.9	43.9
11	21.4	21.4	21.2	21.3	22.2	21.7	20.8	70.1	21.5	21.0
12	26.8	27.6	25.2	25.2	28.2	26.8	26.1	37.6	27.6	25.1
13	37.3	37.1	37.5	37.4	38.0	38.3	35.2	37.6	37.9	37.2
14	42.7	40.8	43.0	42.8	43.5	43.0	41.4	42.3	42.8	42.8
15	27.0	26.9	27.0	27.0	28.2	27.6	28.2	28.8	27.4	27.1
16	33.8	29.1	29.1	29.1	34.5	35.6	22.9	28.9	35.4	29.3
17	47.8	48.0	47.7	47.7	37.6	43.2	54.0	59.3	43.3	47.8
18	49.3	52.3	48.6	48.6	50.4	49.0	55.5	59.3	47.7	48.7
19	43.5	36.5	47.8	47.3	52.8	43.9	92.2	47.2	47.7	47.8
20	154.4	157.0	150.3	150.0	211.5	154.9	141.4	149.1	157.2	150.2
21	29.1	32.8	29.7	29.7	27.8	31.9	34.4	29.8	32.7	29.7
22	31.7	33.9	33.9	33.9	40.4	40.0	29.3	32.9	40.0	33.8
23	26.7	26.6	21.4	26.6	27.1	26.8	29.2	20.7	26.6	29.1
24	21.1	21.1	27.8	21.0	21.3	21.2	22.8	27.4	21.1	16.4
25	16.0	15.9	19.2	15.8	16.1	16.1	16.5	16.8	15.9	17.0
26	15.8	15.8	16.5	15.9	14.8	16.0	15.7	16.7	15.8	14.8
27	14.7	14.6	14.6	14.7	16.4	14.6	13.6	14.1	14.4	15.6
28	60.2	60.2	60.6	60.5	18.3	17.9	178.6	206.0	17.8	60.5
29	107.2	133.2	109.9	109.7		106.9	112.3	110.7	133.0	109.9
30	65.0	194.9	19.0	19.6	29.3	65.2	19.3	19.0	195.0	19.1
Ref	[152]	[153]	[151]	[149]	[14]	[14]	[153]	[151]	[148]	[73]
**C**	**L22**	**L23 ^b^**	**L24**	**L25**	**L26**	**L28**	**L29**	**L30**	**L31**	**L32**
1	34.0	35.4	33.6	53.4	38.6	38.6	26.3	38.6	53.5	38.7
2	22.9	23.7	25.9 *	211.5	24.0	27.4	23.1	27.3	211.4	26.9
3	78.2	80.3	76.4	82.9	81.0	78.9	76.0	78.8	82.9	78.9
4	36.7	37.7	37.5	45.6	38.3	38.9	36.4	38.8	45.6	38.8
5	50.3	51.6	49.9 **	54.6	55.6	55.3	52.4	55.1	54.6	55.3
6	18.0	19.2	18.4	18.5	18.4	18.3	20.9	18.2	18.5	18.2
7	34.4	35.3	34.4	33.8	34.2	34.3	33.0	34.1	33.8	34.3
8	40.9	42.2	41.0	41.2	41.2	40.6	43.1	40.7	43.9	40.8
9	50.7	51.5	50.5 **	50.4	50.5	50.4	45.7	50.2	50.4	50.4
10	37.2	38.3	37.3	44.0	37.3	37.2	39.8	37.1	41.3	37.1
11	20.8	21.9	20.8	21.1	21.1	21.0	23.6	20.8	21.0	20.8
12	27.2	28.8	25.6 *	24.8	25.4	27.3	25.8	27.2	25.0	27.4
13	39.8	39.2	38.7	37.9	37.3	36.6	38.3	36.2	37.2	38.5
14	44.0	43.9	42.6	42.9	42.9	41.8	40.3	42.5	42.8	42.5
15	28.1	28.5	29.5	29.4	27.3	26.8	27.8	26.9	27.0	29.2
16	32.1	36.4	28.8	35.4	29.3	32.8	35.6	28.8	29.1	31.7
17	54.3	44.3	59.3	42.9	48.0	80.3	43.0	47.8	47.7	59.3
18	143.8	52.0	48.0 ***	48.2	49.0	49.2	48.3	49.6	48.6	48.6
19	139.2	38.3	47.5 ***	47.29	48.0	52.2	47.9	52.0	47.7	43.2
20	207.2	158.3	149.8	150.7	150.7	212.2	150.9	213.5	48.6	154.1
21	34.8	33.4	30.0	29.8	30.0	27.5	29.9	27.6	29.7	28.9
22	35.1	40.9	33.2	29.9	34.4	38.7	40.0	33.9	33.9	32.9
23	27.7	28.3	28.2	29.2	28.2	28.0	29.6	27.9	29.3	28.0
24	21.7	22.2	22.2	16.4	16.9	15.4	24.0	15.4	16.3	15.3
25	16.5	16.5	15.9 ****	17.0	16.4	16.2	101.4	16.0	17.0	15.9
26	16.1	16.7	16.1 ****	15.6	16.2	16.0	16.2	15.9	15.6	16.1
27	15.7	15.1	14.2	14.5	15.0	13.2	14.7	14.6	14.7	14.3
28	66.0	18.3	205.6	18.0	60.8		18.0	60.5	60.5	206.3
29		134.8	110.1	109.5	109.9		109.3		109.9	107.4
30	30.7	197.3	19.0	19.3	19.3	29.9	19.3	29.4	19.1	65.0
OCH_3_	21.4	21.3					54.6			
C=O	170.8	173.4			167.7					
1′					126.7					
2′					130.1					
3′					115.8					
4′					159.3					
5′					130.1					
6′					130.1					
7′					116.3					
8′					144.5					
Ref	[154]	[154]	[155]	[73]	[156]	[145]	[54]	[157]	[158]	[145]
**C**	**L33 ^a^**	**L35 ^c^**	**L36**	**L37**	**L38**	**L39**	**L40**	**L41**	**L42**	**L43**
1	39.3	38.4	78.7	38.4	38.4	38.6	38.6	53.5	38.7	38.7
2	28.3	23.8	4.2	23.9	23.8	27.3	27.4	211.5	27.3	27.4
3	78.0	81.3	77.6	55.4	80.6	78.9	78.9	82.9	78.9	79.0
4	39.3	38.1	NR	38.1	37.9	38.8	38.8	45.6	38.8	38.9
5	53.2	55.4	53.1	55.4	55.4	55.2	55.2	54.6	55.5	55.3
6	30.3	18.2	17.8	18.2	18.2	18.2	18.3	18.5	18.2	18.3
7	74.4	34.1	34.0	34.2	34.3	34.2	34.2	33.8	34.3	34.3
8	47.2	41.9	41.4	40.9	40.9	40.7	40.7	41.2	40.8	40.8
9	51.1	50.3	51.4	50.3	50.4	50.3	50.2	50.4	50.4	50.2
10	37.6	37.1	43.5	37.1	37.1	37.1	37.2	44.0	37.1	37.1
11	21.3	21.8	23.8	21.0	21.0	20.7	20.9	21.1	20.7	20.9
12	26.3	25.1	25.0	25.1	25.1	27.5	27.3	24.9	25.5	27.6 ^A^
13	39.3	37.3	38.1	38.1	38.1	37.2	37.0	37.9	38.7	37.7
14	44.0	42.7	37.6	43.0 *	42.9	42.3	42.7	42.9	42.5	42.7
15	34.0	27.0	27.5	27.4	27.5	29.0	27.4	27.4	29.2 *	27.3
16	32.8	29.2	35.6	35.6	35.6	28.7	35.0	35.5	28.8 *	35.4
17	26.7	48.2	42.9	42.8 *	43.0	59.3	43.0	43.0	59.3	43.3
18	49.7	48.8	48.3	48.3	48.3	48.0	49.7	48.2	48.0 **	51.2 ^B^
19	47.7	47.7	48.0	47.7	48.0	51.1	52.6	47.9	47.5 **	36.7 ^C^
20	151.1	150.4	150.8	151.0	151.0	211.8	212.9	150.8	149.7	157.0 ^B^
21	30.9	29.7	29.8	29.9	29.9	27.6	27.6	29.8	29.8	32.6 ^B^
22	37.2	34.0	40.0	40.0	40.0	32.4	39.8	39.9	33.2	39.9
23	28.5	28.0	27.8	28.0	28.0	28.0	28.0	29.3	27.9	28.0
24	16.4	16.7	16.2	16.7	16.6	15.4	15.4	16.4	15.4	15.4
25	16.3	16.2	12.0	16.2 **	16.2	16.1	16.1	17.0	15.9 ***	16.1
26	10.9	16.0	16.2	16.0 **	16.0	15.8	15.9	15.6	16.1 ***	15.9
27	15.1	14.8	14.4	14.5	14.5	14.2	14.5	14.5	14.2	14.4
28	176.6	60.6	17.9	18.0	18.0	206.1	18.0	18.0	205.6	17.8
29	110.0	109.8	109.4	109.4	109.4	30.2	29.2	109.5	110.1	132.9 ^B^
30	19.5	19.1	19.2	19.3	19.3			19.3	19.0	195.1
1′		127.3	167.6	127.1	173.7					
2′		115.3	115.7	115.1	34.9					
3′		144.2	145.1	144.9	25.2					
4′		146.8	127.4	147.3	29.2					
5′		114.2	114.4	113.9	29.3					
6′		122.3	144.0	122.0	29.4					
7′		144.9	146.6	144.9	29.6					
8′		115.8	115.5	115.7	29.7					
9′–14′		168.0	122.4	167.7	29.7					
15′					29.5					
16′					31.9					
17′					22.7					
18′					14.1					
Ref	[153]	[159]	[160]	[159]	[161]	[145]	[145]	[162]	[155]	[163]
**C**	**L44**	**L45**	**L46**	**L47**	**L48**	**L49**	**L50^a^**	**L51**	**L52**	**L53**
1	42.5	42.5	42.1	42.1	38.4	40.9	42.6	38.4	40.1	39.8
2	34.4	34.4	34.4	34.5	27.7	178.5	174.4	36.2	34.5	34.3
3	216.8	216.8	216.7	216.8	81.0	187.5	182.4	217.2	216.3	218.3
4	48.9	48.9	48.9	49.0	37.8	45.6	46.9	47.0	42.2	47.0
5	56.6	56.6	56.5	56.6	55.4	48.2	48.4	49.6	56.5	55.1
6	69.7	69.7	69.6	69.7	18.2	21.3	21.6	19.9	69.8	19.8
7	42.1	42.1	42.2	42.2	34.2	33.7	33.8	33.5	41.9	37.2
8	40.7	40.7	40.0	40.0	40.9	41.8	41.1	40.8	37.5	40.8
9	50.6	50.6	50.6	50.5	50.4	41.7	42.0	52.4	50.9 *	50.0
10	36.8	36.8	36.7	36.7	37.1	40.7	42.3	36.6	34.5	37.1
11	21.8	21.8	21.3	21.3	21.0	19.2	22.2	21.7	21.3	21.5
12	28.7	28.7	26.7	29.7	25.1	24.9	27.3	26.0	25.2	25.7
13	36.8	36.8	37.1	36.8	38.1	37.9	38.6	38.6	37.5	38.6
14	43.9	43.9	42.9	43.2	42.8	43.2	43.5	42.7	42.9	42.6
15	27.7	27.7	27.4	27.4	27.5	27.5	27.9	31.1	29.9	29.8
16	35.6	35.6	35.3	35.3	35.6	35.5	35.6	32.6	32.2	32.2
17	44.6	44.6	43.1	43.0	43.0	43.2	43.2	56.4	56.9	56.5
18	48.5	48.5	48.9	50.6	48.3	48.4	48.9	47.5	49.5 *	49.3
19	50.1	50.1	43.7	42.1	48.0	48.0	43.8	49.6	47.0	47.5
20	73.4	73.4	154.6	157.2	151.0	150.9	156.5	151.1	150.3	150.5
21	29.1	29.1	31.7	32.7	30.0	29.8	32.2	30.0	30.7	30.7
22	40.2	40.2	39.8	39.8	40.0	39.9	40.0	37.3	37.0	33.8
23	24.9	24.9	25.0	25.0	27.1	29.8	27.8	68.0	25.7	26.8
24	23.7	23.7	23.7	23.7	16.2	21.3	24.8	17.2	21.3	21.2
25	17.5	17.5	17.0	16.9	16.5	20.8	20.4	16.0	17.3 **	16.1
26	17.0	17.0	17.1	17.1	16.0	15.9	16.3	15.9	17.1 **	16.0
27	15.2	15.2	14.8	14.7	14.5	14.6	15.0	14.6	15.0	14.8
28	19.2	19.2	17.7	17.8	18.0	18.0	17.9	178.6	181.9	181.2
29	31.7	31.7	106.9	133.2	109.4	109.4	105.9	109.6	109.8	109.9
30	25.2	25.2	65.0	195.1	19.3	19.2	64.3	19.3	19.5	19.5
C=O					171.0					
OCH_3_					21.3					
Ref	[164]	[164]	[146]	[146]	[165]	[73]	[153]	[166]	[167]	[14]
**C**	**L54**	**L55**	**L56**	**L57 ^a^**	**L58 ^d^**	**L59 ^a^**	**L61**	**L62**	**L63**	**L64**
1	39.6	33.9	34.0	34.0	34.0	38.7	159.9	38.7	75.9	75.9
2	34.1	28.1	26.2	26.7	25.1	28.4	125.1	27.4	36.4	36.4
3	218.2	179.1	75.0	75.3	76.9	78.2	205.6	79.0	76.6	76.9
4	47.3	147.5	38.1	38.2	38.5	39.4	44.6	38.8	37.4	37.5
5	54.9	50.4	46.4	49.3	54.9	56.0	53.4	55.3	47.8	47.9
6	19.6	24.5	26.1	18.7	18.0	18.9	19.0	18.3	18.4	18.5
7	33.6	32.8	76.7	34.7	33.9	34.9	33.7	34.3	34.0	34.1
8	40.7	40.9	46.3	41.2	41.2	41.2	41.7	40.8	42.9	41.7
9	49.6	40.4	51.2	50.5	49.9	51.0	44.4	50.4	51.2	51.3
10	36.8	39.2	37.8	37.7	36.7	37.6	39.5	37.1	37.4	43.7
11	21.5	21.4	20.8	21.2	20.5	21.3	21.2	21.0	23.7	23.9
12	29.7	25.4	26.5	28.2	27.1	26.2	25.1	26.7	25.2	25.2
13	37.8	38.3	39.1	38.2	37.6	39.6	38.2	38.0	36.9	37.7
14	42.8	42.8	44.4	43.1	42.6	42.9	43.0	43.0	41.7	42.9 *
15	27.3	30.6	33.0	27.8	31.7	31.3	27.4	27.4	27.1	27.5
16	35.4	32.1	33.4	35.9	36.4	33.0	35.5	35.5	29.2	35.7
17	43.1	56.5	56.3	43.5	55.5	56.7	43.1	42.8	43.6	43.0 *
18	50.9	49.4	49.6	51.3	46.7	47.9	48.1	48.9	48.8	48.1 **
19	40.7	46.9	48.0	41.4	48.7	49.9	47.3	43.8	47.8	48.4 **
20	146.3	150.4	151.4	149.2	150.4	151.4	150.8	154.8	150.3	150.8
21	32.9	29.7	31.2	33.5	30.1	30.4	29.8	31.8	29.7	29.8
22	39.7	36.9	37.7	40.2	38.3	37.7	40.0	39.8	34.0	40.0
23	26.6	113.4	29.0	29.4	28.1	28.8	27.8	28.0	27.7	27.6
24	21.0	23.2	22.4	22.7	15.8	16.4	21.4	16.1	21.9	22.0
25	15.9	20.1	16.1	16.3	15.9	16.5	19.2	16.0	11.7	11.5
26	15.8	15.9	12.1	16.4	16.0	16.5	16.4	15.4	16.2	16.1
27	14.4	14.6	15.1	14.8	14.4	15.0	14.4	14.5	14.8	14.6
28	17.9	176.6	179.0	18.2	177.3	178.9	18.0	17.7	60.6	18.1
29	124.9	109.7	109.9	122.4	109.6	110.0	109.5	107.6	109.8	109.5
30	171.2	19.3	19.5	170.3	19.0	19.6	19.3	65.0	19.0	19.3
OCH_3_		51.3			51.3					
1′			166.3							
2′			117.6							
3′			156.4							
4′			27.0							
5′			20.2							
Ref	[146]	[118]	[153]	[154]	[168]	[169]	[170]	[145]	[146]	[150]
**C**	**L65**	**L66**	**L67**	**L68**	**L69**	**L70**	**L71**	**L72**	**L73**	**L74**
1	79.0	44.5	39.5	39.6	35.5	35.4	33.5	32.5	39.0	38.9
2	37.5	71.3	34.1	34.2	25.6	25.6	28.2	25.3	27.5	27.4
3	75.7	78.5	217.8	218.1	75.9	75.9	76.0	76.4	78.6	78.9
4	38.9	38.2	47.3	47.3	37.8	37.8	38.8	37.6	39.4	38.8
5	53.1	55.2	54.9	54.9	48.9	48.9	48.5	48.2	55.6	54.9
6	18.0	18.1	19.6	19.7	18.0	18.3	21.1	18.4	18.1	18.5
7	34.1	34.2	33.2	33.6	35.1	35.1	34.2	34.4	35.3	37.8
8	41.3	40.9	40.9	40.8	42.6	42.7	40.9	41.1	41.1	42.5
9	51.4	50.9	49.6	49.8	55.6	55.6	50.4	50.5	55.7	51.0
10	43.5	36.9	36.8	36.9	39.1	39.1	37.3	37.3	37.7	37.4
11	23.8	21.1	21.2	21.5	70.5	70.6	20.8	21.2	70.5	21.0
12	25.0	25.2	25.3	25.2	37.6	37.6	25.2	25.3	27.7	25.2
13	38.0	38.0	37.3	38.2	36.3	37.1	37.1	38.0	37.7	37.6
14	42.8	42.9	42.7	42.9	42.9	42.7	42.7	43.0	42.6	47.9
15	27.4	27.3	26.9	27.4	27.0	27.3	27.1	27.6	27.5	69.7
16	35.5	35.6	34.8	35.5	33.8	35.4	29.2	35.8	35.5	46.5
17	42.9	43.0	37.8	43.0	47.8	43.0	48.1	43.2	43.0	43.0
18	48.3	48.3	47.0	48.3	48.2	47.7	47.8	48.2	47.7	48.1
19	47.9	48.0	59.0	48.0	47.6	47.7	48.0	48.2	47.4	47.4
20	150.8	151.0	143.4	150.6	149.8	150.2	150.4	151.2	150.2	150.4
21	29.7	29.9	217.7	29.9	29.1	29.8	29.8	29.9	29.9	30.1
22	39.9	40.0	55.4	40.0	29.3	39.8	34.0	40.2	39.9	39.7
23	27.8	29.6	26.6	26.7	22.3	22.3	28.1	28.2	28.3	27.9
24	14.9	17.1	21.0	21.0	28.7	28.7	16.0	22.4	15.6	15.4
25	11.9	17.1	15.9	16.0	16.3	16.2	16.1	16.4	16.1	16.1
26	16.2	16.0	15.7	15.8	17.2	17.2	16.1	16.2	17.3	16.6
27	14.4	14.5	14.5	14.5	14.1	14.6	14.8	14.7	14.5	8.0
28	18.0	18.0	18.7	18.0	60.2	18.0	60.1	18.2	18.1	19.2
29	109.4	109.3	115.0	109.4	110.2	109.8	109.6	109.6	109.8	109.7
30	19.2	19.3	20.8	19.3	19.1	19.3	18.9	19.5	19.4	19.4
Ref	[171]	[172]	[40]	[145]	[151]	[147]	[173]	[174]	[175]	[176]
**C**	**L75**	**L76 ^c^**	**L77**	**L78^c^**	**L79**	**L80**	**L81**	**L82**	**L83**	**L84**
1	38.7	38.7	34.2	38.2	38.7	38.9	40.7	38.7	38.4	40.7
2	27.3	27.5	27.2	30.6	27.0	27.5	29.7	27.4	27.0	27.6
3	78.3	78.9	79.0	82.3	79.0	78.8	79.1	78.8	76.8	79.1
4	38.8	38.7	38.9	40.9	38.9	38.8	39.6	38.3	41.9	39.6
5	55.6	55.2	55.4	59.5	55.3	55.4	55.6	55.2	49.9	55.5
6	18.2	18.3	18.2	22.0	18.3	18.3	69.0	18.3	18.4	69.1
7	34.1	34.5	34.1	37.5	34.2	33.4	42.1	34.2	34.0	42.4
8	40.8	41.3	40.8	42.7	40.9	40.9	39.9	40.9	40.8	40.4
9	49.4	50.2	50.4	54.5	50.4	50.4	51.9	50.3	50.4	50.9
10	36.8	37.0	42.6	32.2	37.1	37.2	36.7	37.1	37.0	36.6
11	21.4	21.4	20.8	24.7	20.8	21.0	21.1	20.9	21.4	21.6
12	24.8	27.4	25.3	30.8	25.1	26.7	25.3	25.1	26.6	28.8
13	37.4	37.4	37.9	41.3	37.3	38.0	37.2	38.0	37.9	36.6
14	44.1	44.6	42.9	44.8	42.7	42.8	43.0	42.8	42.8	43.8
15	36.8	27.7	27.2	33.0	27.3	27.5	27.5	27.4	27.4	27.5
16	76.9	35.5	35.3	38.0	29.2	35.5	35.5	35.5	35.4	35.5
17	48.6	43.5	42.8	46.4	47.8	43.0	43.1	42.9	43.0	44.6
18	47.6	48.3	48.2	53.3	48.7	48.9	48.4	48.2	48.8	48.4
19	47.4	49.9	47.8	47.4	47.8	43.8	48.0	47.9	43.8	49.9
20	150.0	73.5	150.4	150.7	150.5	155.2	150.9	150.6	154.7	73.5
21	29.8	29.0	29.7	35.4	29.8	31.8	29.9	29.8	31.7	29.2
22	37.7	40.2	40.0	33.1	33.9	39.9	40.0	39.9	39.8	40.2
23	28.0	28.0	28.0	31.2	27.9	28.1	27.6	28.0	72.1	27.7
24	15.0	15.4	15.6	19.3	15.3	15.4	16.6	15.4	11.2	16.9
25	16.0	16.1	60.7	18.6	16.1	16.0	17.7	16.1	16.5	17.8
26	16.0	16.1	16.2	19.2	15.9	16.1	16.9	15.9	16.0	17.2
27	16.0	14.8	14.8	17.8	14.7	14.6	14.9	14.5	14.6	15.2
28	11.6	19.2	18.1	62.9	60.6	17.7	18.0	18.0	17.7	19.2
29	109.6	24.7	109.9	109.8	109.7	106.4	109.4	109.2	106.8	24.8
30	19.0	31.5	19.2	67.8	19.0	64.6	19.3	19.3	65.0	31.6
Ref	[149]	[177]	[178]	[157]	[179]	[148]	[151]	[180]	[145]	[146]
**C**	**L85**	**L86**								
1	40.7	39.5								
2	27.5	34.0								
3	79.1	217.8								
4	39.6	47.2								
5	55.6	54.7								
6	68.9	19.6								
7	42.0	33.4								
8	40.0	40.8								
9	51.0	49.2								
10	36.7	36.8								
11	21.0	21.2								
12	25.3	26.5								
13	36.4	37.7								
14	42.9	43.0								
15	27.1	27.7								
16	33.6	34.5								
17	47.7	43.8								
18	48.8	52.2								
19	47.7	45.0								
20	150.4	139.1								
21	29.7	82.2								
22	29.1	47.7								
23	27.6	26.7								
24	16.9	21.0								
25	17.7	15.9								
26	16.9	15.7								
27	15.1	14.1								
28	60.4	19.3								
29	109.7	124.7								
30	19.1	171.2								
Ref	[151]	[181]								

Ref: References; ^+ 13^C-NMR data of acetylated compound, *, **, ***, **** Values bearing the same superscript are interchangeable; NR: Not reported; Solvent: CDCl_3_; ^a^ Pyridine-d_5_; ^b^ CD_3_COOD; ^c^ CD_3_OD; ^d^ DMSO-d_6_; ^A^ Slightly broadened peak, measured at 25 °C; ^B^ Broad peak, measured at 35°C; ^C^ Very broad peak, determined from HSQC and HMBC spectra at 35°. ^13^C-NMR data for L19, L27 [159], L34, L60, L87 [182,183], L88 [182,183] and L89 were not found. These compounds were identified on the references cited here by comparison of spectroscopic data with those reported in the literature, but we could not get access to the original papers.

**Table 6:** ^13^C-NMR data of oleanane-type pentacyclic triterpenoids isolated from Celastraceae species (2001–2021).

C	O1	O2	O3	O4	O5	O6	O7	O8	O9 ^a^	O10 ^a^
1	38.7	32.8	41.1	40.3	33.8	39.3	40.3	40.3	39.0	72.3
2	33.8	26.6	32.8	34.4	25.5	27.3	34.4	34.2	34.2	35.5
3	216.2	75.9	217.4	218.0	76.1	78.6	218.2	217.8	215.9	72.3
4	47.5	37.4	47.7	47.7	37.5	39.0	47.8	47.7	47.6	40.1
5	54.7	48.2	54.9	55.5	48.8	55.0	55.4	55.3	54.5	47.8
6	18.7	17.5	19.7	19.8	18.3	18.2	19.8	19.7	19.2	17.8
7	30.5	31.1	32.8	33.9	33.2	33.1	32.7	32.6	30.9	32.8
8	40.6	40.7	41.9	42.9	43.3	43.0	43.2	43.2	41.7	45.2
9	50.1	50.4	55.5	50.4	51.2	51.6	48.7	48.5	52.8	53.8
10	36.2	36.6	37.6	37.7	38.3	38.1	37.6	37.5	36.3	42.1
11	52.5	57.0	67.9	76.3	75.9	75.8	81.7	82.0	132.3	200.9
12	57.0	52.9	125.5	121.6	122.5	121.5	122.3	121.2	131.3	128.6
13	87.3	87.9	149.0	149.3	148.3	149.7	150.8	152.8	84.9	169.7
14	41.2	41.3	43.2	42.0	41.8	41.7	42.0	42.3	44.2	44.0
15	26.7	26.7	26.4	26.2	26.1	26.1	26.3	27.9	25.7	26.7
16	21.2	21.6	26.0	26.8	28.1	26.6	28.1	26.6	26.0	26.5
17	43.8	43.7	32.3	32.4	34.8	32.3	34.9	33.1	41.9	32.5
18	49.6	49.1	46.7	47.2	46.3	46.8	46.5	47.0	51.1	47.6
19	37.8	32.5	46.3	46.4	46.7	46.3	46.9	46.7	32.4	45.2
20	31.5	36.8	31.0	31.1	36.3	31.1	36.5	31.1	36.7	31.0
21	34.3	29.7	34.6	34.7	73.9	34.5	74.0	34.6	30.9	34.5
22	27.0	27.7	36.8	36.9	45.2	36.8	45.2	36.9	30.6	36.7
23	25.9	28.0	26.7	26.7	28.6	28.0	26.7	26.9	26.2	28.8
24	21.1	21.8	21.5	21.5	22.4	15.4	21.6	21.5	21.0	16.2
25	16.4	17.0	16.2	16.4	16.7	18.0	16.4	18.0	17.3	18.0
26	18.7	20.1	17.9	18.1	18.2	16.8	18.2	16.2	19.4	19.1
27	19.8	18.1	26.1	25.2	25.2	25.1	24.7	24.7	19.6	23.6
28	179.2	179.1	28.4	28.5	28.4	28.3	28.5	28.5	77.1	28.8
29	33.2	26.6	33.0	33.2	28.9	33.1	29.1	33.2	28.9	33.0
30	23.6	65.7	23.6	23.6	16.9	23.5	17.0	23.6	65.0	23.5
OCH_3_				53.7	53.7	53.7				
Ref	[184]	[185]	[186]	[104]	[187]	[188]	[187]	[187]	[189]	[190]
**C**	**O11**	**O12**	**O13**	**O14**	**O15**	**O16**	**O17**	**O18 ^b^**	**O19 ^a^**	**O20**
1	39.6	39.4	39.3	40.0	38.1	39.8	48.6	47.6	47.7	46.6
2	33.8	34.2	34.2	34.5	24.0	34.0	69.1	69.6	68.7	69.0
3	218.1	217.7	217.8	217.5	80.3	218.3	83.1	85.9	85.7	84.0
4	47.0	47.5	47.5	48.1	37.5	47.2	39.3	44.3	43.9	39.3
5	54.6	55.4	55.3	55.7	55.0	54.8	55.5	57.3	56.5	55.4
6	19.4	19.7	19.6	19.1	18.8	19.6	17.7	19.8	19.2	18.4
7	33.7	32.2	32.1	32.3	32.2	33.7	34.0	34.3	33.5	33.1
8	40.6	39.9	39.7	45.2	39.6	40.6	43.5	40.9	40.0	39.4
9	50.1	46.9	46.8	61.3	47.2	50.4	56.8	49.2	48.3	47.6
10	36.7	36.7	36.7	37.0	38.0	36.8	40.1	39.2	38.3	38.3
11	21.3	23.8	23.7	199.3	23.3	21.5	72.3	25.1	24.2	23.6
12	25.7	123.6	122.4	128.2	123.1	26.0	211.1	124.6	122.4	124.5
13	37.6	142.5	143.8	168.9	142.6	38.9	82.4	144.4	144.4	140.5
14	43.6	41.9	41.8	43.8	41.8	43.2	45.3	42.5	42.1	42.6
15	27.3	25.6	26.0	26.1	25.2	27.3	22.6	30.3	29.1	24.4
16	36.3	23.6	28.2	30.9	18.0	36.8	30.5	27.9	23.1	25.3
17	36.9	39.6	35.0	37.3	31.7	34.5	33.6	47.0	47.0	35.3
18	142.9	48.0	46.7	43.0	46.1	147.5	49.0	45.0	41.8	43.5
19	127.7	41.4	47.0	45.5	40.0	124.7	38.5	82.3	46.2	33.9
20	40.0	151.6	36.3	31.3	42.6	28.0	31.4	35.9	30.8	39.6
21	73.1	69.1	74.0	34.1	36.6	27.9	34.1	29.4	34.0	39.9
22	43.5	45.9	45.3	21.8	75.2	37.3	39.0	33.1	32.2	83.2
23	26.6	26.6	26.5	21.7	23.8	26.8	28.7	23.8	24.2	28.7
24	20.7	21.6	21.5	26.7	16.5	20.9	16.6	66.1	65.7	16.9
25	16.3	15.3	15.2	16.0	15.3	16.5	17.2	17.5	17.3	17.0
26	15.7	16.8	16.7	18.7	16.5	15.9	20.7	17.7	17.4	17.1
27	13.9	25.8	25.8	23.7	25.9	14.5	18.5	25.1	26.1	24.1
28	26.7	68.8	28.3	69.6	24.9	25.5	31.3	178.6	176.6	25.0
29	28.5		29.1	23.6	177.9	70.5	25.1	28.6	33.2	182.5
30	21.4	103.6	16.9	33.2	20.3	25.6	31.9	25.2	23.8	21.1
OCH_3_					51.7					
1′					173.5			93.9	93.7	
2′					34.6			78.6	78.6	
3′					23.3			78.0	79.0	
4′–11′					28.9–29.4			70.8	70.6	
5′								78.8	79.2	
6′								62.2	62.0	
1″								103.4	104.6	
2″								75.7	76.0	
3″								77.8	78.4	
4′’								72.5	72.7	
5″								77.9	78.4	
6″								63.7	63.8	
12′					31.7					
13′					NR					
14′					13.9					
Ref	[191]	[61]	[96]	[192]	[68]	[191]	[193]	[194]	[194]	[61]
**C**	**O21**	**O22 ^a^**	**O23**	**O24**	**O25**	**O26**	**O27**	**O28 ^a^**	**O29**	**O30**
1	79.6	40.0	39.5	34.7	33.4	34.1	39.1	212.9	40.3	38.7
2	44.5	34.4	34.3	29.4	25.4	22.7	27.3	45.4	23.9	23.8
3	172.4	215.9	218.1	98.0	75.8	78.0	78.7	79.0	80.2	81.2
4	56.0	47.8	47.6	40.0	37.5	36.7	39.2	40.1	38.3	37.1
5	85.0	55.1	55.3	50.2	48.4	49.7	55.0	54.9	55.2	56.0
6	33.2	19.0	19.9	19.4	17.4	17.7	17.5	18.1	17.7	18.2
7	27.6	32.1	32.8	30.8	32.6	32.5	32.9	34.4	34.0	35.1
8	43.2	45.3	40.0	38.5	43.5	43.5	46.3	40.7	44.1	40.3
9	55.2	61.2	46.9	41.6	61.6	61.6	60.4	42.8	54.2	50.5
10	44.5	37.0	36.9	35.0	37.1	37.0	37.4	53.5	39.3	37.1
11	67.3	198.9	23.9	23.6	200.3	200.1	195.3	24.4	75.3	24.8
12	133.5	128.4	124.7	122.3	128.3	128.2	142.8	26.7	202.2	124.2
13	139.8	170.6	140.5	143.4	169.4	169.3	138.3	39.6	83.2	144.1
14	42.6	43.7	42.9	41.6	45.6	45.6	41.5	43.7	44.8	42.1
15	19.8	26.7	25.4	25.8	25.7	25.8	26.5	27.8	22.9	27.2
16	34.1	26.9	24.5	28.0	22.0	21.5	26.7	31.3	39.1	27.5
17	51.4	32.5	35.5	36.0	37.0	37.0	31.3	32.5	33.5	32.0
18	37.8	47.3	43.7	47.0	42.7	42.7	38.7	140.7	49.1	47.4
19	40.5	40.8	40.0	46.5	44.9	45.0	41.3	132.6	38.3	46.9
20	47.7	36.0	39.7	36.0	31.0	31.0	30.9	32.5	31.5	31.1
21	215.9	29.9	39.7	73.8	33.9	33.8	34.7	33.4	34.5	34.8
22	40.4	36.4	83.3	45.0	30.6	30.6	36.9	31.1	30.0	37.2
23	21.0	21.5	27.0	27.0	28.4	28.0	28.0	28.6	28.2	28.0
24	20.3	26.5	21.7	18.0	22.3	21.9	15.5	16.6	16.4	16.6
25	13.8	15.9	15.2	67.7	16.3	16.3	16.5	16.1	16.4	15.5
26	19.1	18.5	16.7	17.0	18.6	18.6	18.7	17.0	20.9	16.5
27	22.6	23.4	25.3	25.1	23.6	23.5	23.2	15.1	18.9	25.7
28	26.8	28.7	24.2	28.1	69.7	69.7	29.2	63.6	31.4	28.7
29	28.4	28.2	182.7	16.6	32.9	32.9	33.1	31.4	32.3	33.2
30	21.3	65.3	21.2	28.8	23.4	23.4	23.4	29.7	24.5	23.7
OCH_3_						21.7			21.4	
C=O						170.7			171.2	
1′									129.7	127.4
2′									130.1	115.4
3′									128.6	144.1
4′									133.2	146.6
5′									128.6	114.3
6′									130.1	122.2
7′									165.6	144.8
8′										116.0
9′										167.8
Ref	[117]	[190]	[195]	[196]	[154]	[154]	[54]	[190]	[197]	[198]
**C**	**O31**	**O32**	**O33**	**O34**	**O36**	**O37**	**O38**	**O39**	**O40**	**O41**
1	38.1	38.6	38.4	53.5	39.2	38.6	38.7	38.5	42.2	42.0
2	24.4	23.7	23.7	211.1	26.4	27.2	27.3	22.5	34.4	34.1
3	80.7	80.6	80.6	83.1	78.3	78.9	79.0	80.8	216.7	216.5
4	37.6	80.6	37.8	45.9	39.3	38.7	38.8	37.8	49.0	48.7
5	51.3	55.6	55.4	54.8	55.0	55.2	55.3	55.5	56.4	56.2
6	18.2	18.2	18.3	18.8	17.5	18.3	18.5	18.5	69.7	69.4
7	32.1	34.9	32.7	33.0	32.8	33.1	32.8	32.8	42.2	42.0
8	38.0	40.8	39.9	40.1	43.4	39.3	38.8	39.7	39.9	39.6
9	153.9	51.1	47.7	47.7	61.8	47.5	47.7	47.8	51.3	51.1
10	40.7	37.2	36.9	43.8	37.1	37.0	37.6	37.1	36.8	36.6
11	115.9	21.1	23.6	23.7	200.2	23.5	23.6	23.9	21.4	21.2
12	120.7	26.2	121.8	124.1	128.3	124.6	121.8	121.9	26.1	26.0
13	147.2	38.4	145.2	140.7	170.4	140.2	145.1	145.4	38.1	37.4
14	42.8	43.3	41.8	42.9	45.4	39.5	41.8	41.9	43.5	43.3
15	25.6	27.5	26.2	24.5	26.4	25.2	26.2	26.4	27.5	27.4
16	27.2	37.7	27.0	25.4	27.3	24.3	27.0	26.0	36.9	37.3
17	32.0	34.3	32.5	35.5	32.3	35.2	32.5	32.7	34.5	34.2
18	45.6	142.7	47.4	43.6	47.6	43.4	47.4	47.4	147.6	142.4
19	46.8	129.8	46.9	40.0	45.2	39.8	46.9	47.0	124.9	129.8
20	31.1	32.3	31.1	39.7	31.0	42.5	31.1	31.3	37.4	32.1
21	34.6	33.4	34.8	34.0	34.4	33.8	34.8	23.9	28.0	33.0
22	37.1	37.4	37.2	83.2	36.5	83.1	37.2	121.9	37.4	37.1
23	28.7	28.0	28.1	29.6	28.7	28.1	28.2	145.4	25.1	23.4
24	16.9	16.6	16.8	16.8	16.4	15.6	15.5	41.9	23.6	24.8
25	20.0	16.7	15.6	16.7	15.7	15.6	15.6	26.4	17.3	16.9
26	21.0	16.1	16.9	16.9	18.7	17.0	16.9	26.0	17.7	17.6
27	25.3	14.5	26.0	24.3	23.4	25.0	26.0	32.7	14.9	14.5
28	28.2	25.3	28.4	25.2	28.2	24.0	28.4	47.4	25.5	25.0
29	33.2	31.3	33.3	182.5	33.0	182.4	33.3	47.0	70.5	31.2
30	23.7	29.2	23.7	21.2	23.5	21.0	23.7	31.3	25.7	29.0
1′	127.8	173.7	173.5					173.9		
2′	115.5	34.5	34.9					35.0		
3′	143.8	25.2	25.2					25.4		
4′	146.2	29.2	29.2					29.4–29.9		
5′	114.3	29.3	29.3					29.4–29.9		
6′	122.4	29.3	29.4					29.4–29.9		
7′	144.4	29.7	29.6					29.4–29.9		
8′	116.4	29.7	29.7					29.4–29.9		
9′–13′	167.4	29.7	29.7					29.4–29.9		
14′		29.7	29.7					31.2		
15′		29.7	29.5					23.0		
16′		31.9	31.9					14.4		
17′		22.7	22.7							
18′		14.1	14.1							
Ref	[198]	[161]	[199]	[166]	[200]	[111]	[201]	[202]	[191]	[191]
**C**	**O42**	**O43 ^c^**	**O44**	**O45**	**O47 ^d^**	**O50**	**O51 ^a^**	**O52 ^c^**	**O53**	**O54**
1	38.9	42.5	40.6	128.4	40.1	39.3	47.8	34.6	41.5	40.1
2	34.0	35.3	34.8	143.9	35.0	34.2	68.6	42.2	175.2	37.4
3	216.9	217.0	218.6	201.2	219.0	217.9	83.8	76.6	180.3	213.6
4	47.1	48.6	48.4	44.0	48.0	47.5	39.8	37.2	46.0	44.8
5	52.4	56.8	55.9	53.9	56.0	55.3	55.9	48.5	48.9	53.6
6	30.3	20.9	20.1	18.8	20.3	19.7	18.9	37.9	20.7	23.9
7	72.9	32.2	31.5	32.7	26.7	32.1	33.2	18.1	32.1	31.6
8	43.3	44.3	44.4	40.5	40.5	39.8	48.2	32.8	39.3	39.1
9	46.9	55.4	50.7	43.1	47.5	46.8	48.2	43.2	39.4	45.3
10	36.7	38.9	38.1	38.4	37.8	36.7	38.6	55.6	41.6	36.7
11	23.5	68.3	76.6	23.6	24.0	23.7	23.5	67.1	23.9	22.0
12	122.5	128.7	122.8	123.0	123.4	123.3	122.5	126.1	124.6	122.5
13	143.8	147.2	148.9	143.4	143.5	143.2	144.9	147.7	140.1	143.8
14	45.3	43.0	42.2	41.9	43.4	41.9	42.2	41.4	43.3	41.8
15	29.2	27.3	26.5	25.7	26.3	25.4	28.3	25.1	24.4	27.6
16	22.3	28.0	27.2	28.1	29.0	22.2	23.7	26.5	25.3	23.0
17	36.7	33.1	32.3	37.4	39.5	36.9	46.7	31.5	35.4	46.5
18	43.0	49.0	48.1	43.9	49.8	41.1	41.7	47.0	43.6	41.3
19	46.4	43.3	42.6	39.9	44.2	39.9	42.0	30.9	39.8	45.8
20	31.0	44.8	43.3	42.5	46.0	42.4	31.0	43.5	39.6	30.7
21	34.1	34.2	33.3	36.2	40.6	28.5	34.3	30.9	33.9	33.8
22	30.8	39.5	38.6	75.8	78.7	29.8	33.3	38.0	83.2	32.4
23	26.4	27.4	27.0	27.3	26.6	21.6	29.4	28.4	28.5	116.0
24	21.5	22.2	21.9	21.9	21.7	26.6	16.9	22.1	23.4	
25	15.0	17.0	16.7	20.0	15.5	15.3	17.5	16.4	19.2	13.1
26	9.7	18.9	18.4	17.5	17.2	16.7	17.7	17.8	17.2	17.0
27	25.9	26.3	25.5	25.4	26.2	25.4	26.2	26.5	23.7	25.8
28	69.6	29.3	29.0	19.9	25.8	69.0	180.2	28.4	25.2	180.4
29	33.1	29.3	29.3	184.0	34.3	184.5	33.3	28.0	182.4	33.0
30	23.8	178.9	183.3	23.8	181.0	19.2	23.8	179.2	20.7	23.5
OCH_3_			54.1						52.2	
Ref	[154]	[203]	[203]	[61]	[204]	[61]	[157]	[205]	[61]	[166]
**C**	**O55**	**O56**	**O58 ^a^**	**O60 ^a^**	**O61**	**O62 ^a^**	**O63 ^b^**	**O65 ^b^**	**O67 ^a^**	**O68**
1	36.8	39.6	33.6	38.9	33.6	37.9	38.2	38.9	38.9	38.5
2	32.4	34.4	26.2	28.1	26.2	25.1	26.6	28.2	27.6	26.7
3	217.7	217.8	75.2	78.0	70.8	81.9	79.7	79.6	73.7	78.7
4	39.1	47.7	37.8	39.7 *	47.4	55.4	38.0	40.1	42.9	38.6
5	55.3	55.6	49.1	55.8	49.1	46.9	48.0	56.5	48.8	55.1
6	19.5	20.0	18.6	18.8	19.7	20.3	19.5	19.0	18.7	18.2
7	33.8	32.5	32.9	33.2	32.7	32.6	33.7	33.4	33.6	32.5
8	39.3	40.1	40.3	37.3 *	39.8	39.9	39.6	41.2	39.8	39.7
9	47.4	47.1	47.7	48.1	46.7	47.1	49.6	48.9	48.2	47.5
10	46.9	37.0	37.3	37.9	37.6	37.9	39.9	37.8	37.3	36.8
11	23.6	24.0	23.8	23.8	23.5	23.5	20.2	24.4	23.8	23.4
12	122.4	123.0	123.2	122.7	121.7	120.9	124.3	123.5	122.7	123.1
13	143.6	144.3	144.4	144.7	145.1	143.0	144.7	144.5	145.0	143.2
14	41.7	42.1	42.0	42.2	41.9	42.0	43.2	43.5	42.2	41.5
15	32.2	27.3	25.9	28.3	26.0	35.9	24.7	26.5	28.4	25.2
16	21.4	26.3	23.0	24.0	26.9	73.1	27.9	29.0	23.8	21.8
17	46.6	32.7	37.6	46.6	32.5	48.6	41.2	39.5	46.7	36.7
18	41.0	46.4	41.7	41.6	47.3	41.2	56.6	49.9	42.0	41.1
19	45.8	40.5	41.4	42.0	46.7	47.5	40.0	44.3	46.5	40.1
20	30.7	42.8	42.8	35.9	31.1	30.9	43.9	46.2	31.0	42.1
21	32.4	29.2	29.5	29.6	34.7	35.8	76.0	40.7	34.3	28.4
22	26.4	36.1	30.8	32.9	37.1	32.8	41.5	78.8	33.3	29.7
23	27.7	26.8	29.2	28.4	183.2	206.4	28.7	28.3	68.2	27.9
24	15.0	21.7	22.7	16.5	24.2	10.2	17.4	16.1	13.1	15.4
25	15.0	15.5	15.7	15.3	13.1	15.4	16.1	15.7	16.0	15.3
26	17.0	17.0	17.0	17.4	16.7	17.2	16.3	17.3	17.5	16.5
27	25.8	26.1	26.0	26.2	25.9	27.1	26.7	26.3	26.2	25.7
28	183.9	28.4	68.3	180.2	28.4	174.8	21.0	25.8	180.4	68.6
29	33.0	185.1	181.5	28.2	33.3	33.2	182.3	34.1	33.3	181.7
30	23.5	19.4	20.1	65.5	23.7	24.5	25.2	181.1	23.8	19.1
Ref	[166]	[102]	[61]	[206]	[207]	[208]	[209]	[204]	[157]	[61]
**C**	**O69 ^b^**	**O70**	**O71 ^a^**	**O72 ^a^**	**O73**	**O75 ^a^**	**O76**	**O77**	**O81**	**O82**
1	42.0	38.1	213.0	38.5	38.5	49.6	38.3	78.7	34.9	32.5
2	28.0	23.4	45.4	28.0	27.4	170.6	23.5	172.2	25.2	26.0
3	80.0	81.0	78.4	78.1	78.7	182.0	80.8	110.9	76.0	76.1
4	39.7	37.7	40.0	39.5	38.7	45.4	37.9	36.4	37.4	37.3
5	57.1	55.4	54.7	55.3	55.2	55.2	55.2	48.8	48.7	47.3
6	68.7	18.2	18.2	18.8	18.3	20.7	18.3	17.6	18.3	18.3
7	41.6	32.6	33.2	32.9	32.6	33.4	32.6	34.5	33.0	32.5
8	40.7	39.4	39.7	41.1	39.3	41.1	39.8	41.1	43.6	37.0
9	49.4	47.6	39.7	54.8	47.6	45.9	47.5	41.0	56.4	48.9
10	37.6	37.0	52.7	37.1	37.0	38.5	36.8	40.1	38.2	33.0
11	24.5	23.4	25.7	126.0	23.1	74.5	23.7	22.1	67.6	23.4
12	124.0	122.6	123.2	127.0	122.1	121.4	121.6	24.2	126.1	122.6
13	144.4	143.6	144.1	136.6	143.4	149.8	145.2	133.5	148.0	143.7
14	43.3	41.6	42.5	42.5	41.6	43.1	41.7	45.2	41.8	41.7
15	28.8	27.7	28.3	33.3	27.7	25.8	26.1	26.4	26.0	26.0
16	24.1	22.9	23.7	25.6	23.4	27.5	26.9	39.3	28.0	32.5
17	47.9	46.6	46.8	48.7	46.6	32.9	32.5	34.6	34.7	25.2
18	41.9	41.0	42.3	133.8	41.3	47.2	47.2	133.9	45.8	35.0
19	41.4	45.7	46.2	41.0	45.8	41.6	46.7	38.7	46.7	46.6
20	36.8	30.7	31.0	32.8	30.6	36.1	31.1	33.3	36.3	50.0
21	29.3	33.6	34.3	37.5	33.8	30.0	34.7	35.4	73.9	74.0
22	33.1	32.5	33.2	36.3	32.3	36.6	37.1	36.4	45.1	36.3
23	28.4	28.1	29.0	28.5	28.1	28.2	28.1	19.3	28.3	45.4
24	17.6	17.2	16.8	16.0	15.6	23.3	16.8	24.3	22.3	28.2
25	17.3	15.2	15.0	18.4	15.3	17.7	15.6	15.0	16.7	22.3
26	18.8	16.7	18.1	17.1	16.8	17.1	16.9	18.1	18.1	16.8
27	26.5	25.9	26.0	20.1	26.0	25.5	26.0	21.3	26.3	15.2
28	181.8	184.0	180.1	178.9	181.0	28.4	28.4	23.8	28.5	26.0
29	74.4	33.1	33.3	32.4	33.1	28.2	33.3	32.3	28.9	28.3
30	19.5	23.6	23.8	24.4	23.6	65.6	23.7	24.0	16.9	29.0
1′							167.1			
2′							116.2			
3′							144.6			
4′							127.1			
5′							109.2			
6′							146.8			
7′							147.8			
8′							114.6			
9′							123.0			
OCH_3_		21.3					55.9			
C=O		171.1								
Ref	[210]	[211]	[157]	[212]	[27]	[190]	[213]	[214]	[187]	[187]
**C**	**O84**	**O85**	**O86**	**O88**	**O89**	**O90**	**O91**	**O92**	**O93**	**O94**
1	39.5	39.2	38.6	38.6	38.2	39.6	39.9	41.3	41.4	38.6
2	27.4	28.2	27.2	19.0	23.7	33.8	34.0	27.6	27.7	27.1
3	78.7	78.1	79.0	79.0	80.9	218.0	217.8	78.5	78.5	78.7
4	39.0	39.4	38.8	38.8	37.8	47.0	47.2	39.5	39.1	38.6
5	55.1	55.9	55.2	55.2	55.2	54.6	55.0	55.7	55.9	55.2
6	18.4	18.9	18.4	18.4	18.3	19.4	19.7	17.9	18.0	18.0
7	32.9	33.3	32.6	32.6	32.6	33.4	34.0	35.5	35.6	34.5
8	43.3	40.1	39.8	39.8	39.8	40.3	40.7	42.8	42.9	40.7
9	49.7	48.1	47.6	47.6	47.7	50.2	50.6	56.3	56.5	50.8
10	37.9	37.3	36.9	36.2	36.9	33.7	37.0	39.4	39.5	36.9
11	81.7	23.9	23.6	23.5	23.5	21.3	21.7	70.9	71.1	20.7
12	121.2	122.5	122.3	122.2	121.7	25.9	26.3	38.3	38.6	25.7
13	153.2	144.9	144.2	144.8	145.2	38.7	38.6	37.7	37.3	37.5
14	41.8	42.5	41.7	41.7	41.7	42.5	43.4	42.8	43.0	43.5
15	26.4	26.5	25.6	26.1	28.3	27.2	27.6	27.3	27.5	27.1
16	27.4	28.7	22.0	27.2	26.2	33.9	37.7	31.5	37.6	36.4
17	32.3	38.0	36.9	32.9	32.5	40.6	34.4	39.0	34.3	37.0
18	46.9	45.4	42.3	46.3	47.3	143.4	142.6	137.5	141.6	143.1
19	46.9	46.9	46.5	29.0	46.8	127.8	130.0	134.5	129.9	127.5
20	31.2	30.9	31.0	41.0	31.2	45.1	32.4	32.2	32.4	37.0
21	34.7	42.3	34.1	36.2	34.7	215.2	33.4	33.2	33.4	73.1
22	37.0	75.6	31.0	36.9	37.1	52.3	37.4	31.1	37.3	43.7
23	28.2	28.8	15.5	15.6	28.0	24.5	26.9	28.2	28.2	27.7
24	15.5	15.9	28.1	28.1	16.7	20.7	21.0	15.5	15.6	15.8
25	18.3	16.6	15.5	15.5	15.6	13.3	16.0	16.8	17.4	16.4
26	16.8	17.3	16.7	16.8	16.8	15.6	16.0	17.4	16.9	15.8
27	24.7	25.8	25.9	26.0	25.9	14.7	14.5	14.4	14.3	13.9
28	28.5	28.8	69.7	16.8	26.9	25.6	25.3	65.4	25.3	26.7
29	33.3	33.3	33.2	26.9	33.3	28.8	31.3	29.7	31.3	28.5
30	23.7	21.2	23.6	74.8	23.7	26.3	29.2	30.5	29.2	21.4
OCH_3_					21.3					
C=O					171.0					
Ref	[215]	[216]	[217]	[218]	[219]	[191]	[220]	[191]	[220]	[191]
**C**	**O95**	**O96**	**O97**	**O98**	**O99**	**O100**	**O101**	**O102**		
1	38.9	40.8	38.5	38.8	31.6	38.8	34.8	38.3		
2	27.4	27.5	27.4	27.9	25.9	27.9	33.0	22.7		
3	78.9	79.1	79.0	78.6	75.7	78.6	217.0	80.9		
4	38.9	39.7	39.0	38.9	37.7	38.9	47.2	37.9		
5	55.4	55.6	55.7	51.2	44.9	51.2	48.1	55.3		
6	18.2	69.0	18.3	18.4	18.2	18.4	19.7	18.3		
7	34.6	42.2	34.7	32.2	31.9	32.2	30.4	32.5		
8	40.7	39.7	40.8	37.0	40.7	37.0	46.6	39.8		
9	51.1	51.8	51.3	154.3	154.9	154.3	53.7	47.5		
10	37.2	36.8	37.3	40.7	38.8	40.7	37.8	36.9		
11	21.0	21.2	21.2	115.8	115.3	115.8	72.1	23.5		
12	26.1	26.2	26.2	120.8	121.4	120.8	120.5	121.6		
13	38.8	37.6	39.0	147.1	145.2	147.1	152.8	145.2		
14	43.2	43.5	43.4	42.8	42.8	42.8	41.7	41.7		
15	27.4	27.6	27.6	25.7	25.5	25.7	26.7	26.1		
16	36.9	37.3	37.7	27.3	28.5	27.3	26.6	26.9		
17	34.5	34.3	34.4	32.2	34.5	32.2	32.8	32.6		
18	147.8	142.8	142.8	45.6	44.9	45.6	48.0	47.2		
19	124.4	129.9	129.8	46.9	47.1	46.9	46.1	46.8		
20	27.1	32.3	32.3	31.1	36.3	31.1	31.1	31.1		
21	28.0	33.3	33.4	34.7	73.9	34.7	34.6	34.7		
22	37.5	40.8	37.4	37.2	45.3	37.2	36.9	37.1		
23	27.9	27.6	28.0	28.0	28.3	28.8	27.9	28.1		
24	15.4	16.8	15.4	16.6	22.4	15.1	19.4	16.8		
25	16.7	18.1	16.1	15.5	25.1	20.1	19.1	15.5		
26	16.1	17.5	16.7	16.5	20.9	21.0	99.1	16.8		
27	14.7	14.8	14.6	25.7	20.1	25.3	23.6	25.9		
28	25.5	25.2	25.3	28.7	28.6	28.3	28.7	28.4		
29	70.6	31.3	31.3	33.2	28.9	23.7	33.3	33.3		
30	25.7	29.1	29.2	23.7	16.9	33.2	23.7	23.7		
1′								127.3		
2′								109.3		
3′								147.0		
4′								146.6		
5′								114.8		
6′								120.8		
7′								76.4		
8′								76.0		
9′								62.9		
OCH_3_								56.0		
1′								128.5		
2′								116.6		
3′								143.8		
4′								144.8		
5′								117.5		
6′								122.2		
7′								143.7		
8′								117.3		
9′								167.0		
CH_3_								20.7		
C=O								170.4		
Ref	[191]	[191]	[220]	[198]	[187]	[68]	[221]	[126]		

Ref: References; * Values bearing the same superscript are interchangeable; NR: Not reported; Solvent: CDCl_3_; ^a^ C_5_D_5_N; ^b^ CD_3_OD; ^c^ CD_3_COCD_3_; ^d^ CD_3_OD; ^13^C-NMR data of some compounds were not found. In these cases, the reported identification was performed by comparison of other physical data: O59 [222], O74 (m.p., IR, MS, ^13^C-NMR of acetylated compound) [102], O78 (m.p., MS, ^1^H-NMR, UV, [α]_D_) [223], O79 (m.p., MS, IR, UV, [α]_D_) [224], O80, O83 and 087. ^13^C-NMR data of O35 [225], O46 [226], O48 [226], O49 [166], O57 [226], O64 [227], O66 [226] were reported, but the chemical shifts were not attributed to each carbon atom.

**Table 7:** ^13^C-NMR data of ursane-type pentacyclic triterpenoids isolated from Celastraceae species (2001–2021).

C	U1	U2	U3 ^a^	U4	U5	U6	U7	U8	U9	U10
1	41.4	41.1	42.5	40.8	40.8	41.4	39.2	39.0	39.0	47.2
2	34.2	34.5	35.1	27.4	27.7	34.3	34.5	28.3	28.3	69.1
3	217.6	220.9	217.2	78.4	78.6	217.6	220.2	76.9	77.0	85.0
4	47.6	51.1	48.3	39.0	39.1	47.7	51.3	52.9	52.9	43.4
5	55.0	55.5	56.1	54.8	54.9	55.6	55.9	56.3	56.4	56.1
6	19.9	19.3	20.5	18.4	18.4	19.7	19.4	18.5	18.5	18.3
7	36.4	36.6	34.4	37.2	37.8	33.3	36.8	37.5	37.6	34.4
8	43.7	43.6	43.5	43.7	43.8	37.6	43.8	43.9	43.9	42.9
9	52.8	52.4	53.8	51.6	50.5	54.4	44.9	45.3	45.1	46.4
10	37.7	37.3	38.5	38.4	38.3	43.1	37.6	38.5	38.5	39.2
11	69.5	70.0	70.2	68.0	75.1	68.7	76.5	76.5	76.6	76.7
12	146.3	145.5	148.4	146.2	145.0	129.0	142.9	143.7	143.9	141.9
13	115.1	116.3	113.2	116.5	123.1	142.8	119.1	118.0	119.1	118.3
14	46.8	46.9	44.2	46.6	47.2	42.5	46.6	46.5	46.5	40.6
15	68.0	68.1	37.8	68.2	68.1	28.0	68.0	67.9	68.0	27.1
16	38.1	38.9	65.9	38.7	38.8	26.6	39.0	38.2	38.9	27.5
17	33.7	34.2	39.5	34.1	34.1	33.8	34.2	33.7	34.2	33.3
18	42.0	47.5	49.9	46.1	46.7	58.5	47.5	42.2	47.5	47.7
19	41.2	40.5	41.9	40.3	38.9	39.4	40.5	41.3	40.5	40.8
20	71.6	39.3	40.7	39.6	39.5	39.5	39.3	71.4	39.3	39.5
21	35.6	31.0	31.8	31.0	31.0	31.1	31.1	35.5	31.0	31.2
22	35.7	41.0	36.8	41.1	41.1	41.3	41.1	35.9	41.2	41.6
23	26.8	22.4	27.5	28.3	28.4	26.9	22.5	19.4	19.4	23.2
24	21.4	65.9	22.1	15.8	15.8	21.2	65.9	207.8	207.7	65.6
25	16.3	17.3	16.9	16.7	16.4	16.2	16.6	15.5	15.4	18.1
26	18.5	18.3	18.9	18.7	19.2	17.5	18.3	18.7	18.7	18.1
27	17.6	17.6	26.0	18.0	17.6	23.0	17.3	17.5	17.5	23.9
28	29.0	29.3	23.6	29.3	29.2	28.7	29.1	28.8	29.0	28.5
29	11.5	16.8	17.8	17.6	18.3	18.0	17.0	12.0	17.0	17.0
30	29.8	21.0	22.0	21.0	21.1	21.5	21.1	29.9	21.1	21.2
Ref	[193]	[193]	[190]	[54]	[54]	[228]	[193]	[193]	[193]	[193]
**C**	**U11**	**U12**	**U13**	**U14**	**U15**	**U16**	**U17**	**U18 ^a^**	**U19**	**U20**
1	40.8	39.0	38.7	39.0	38.7	39.0	39.9	40.6	39.9	40.3
2	34.4	27.5	27.8	27.5	27.9	27.5	27.5	28.5	26.9	34.2
3	218.3	78.6	80.4	78.6	80.5	78.7	78.7	77.9	78.9	217.9
4	47.6	39.1	43.1	39.1	43.1	39.2	30.9	38.5	39.1	47.6
5	55.3	55.1	55.7	55.1	56.1	55.5	54.9	55.8	55.3	55.2
6	19.7	18.4	18.5	18.4	18.3	18.2	18.6	18.8	18.4	19.6
7	33.2	37.3	37.6	37.4	34.4	34.2	36.5	33.6	33.5	33.0
8	42.7	44.2	44.1	44.2	40.6	40.6	44.2	43.2	42.9	43.2
9	50.9	46.9	46.3	46.3	46.5	46.4	52.7	53.1	52.6	47.2
10	37.6	38.4	38.1	38.5	38.1	38.4	38.1	39.7	38.2	37.3
11	77.0	76.4	76.6	76.4	76.8	76.7	76.5	76.7	76.8	81.7
12	124.2	144.0	143.0	143.1	142.1	142.1	125.2	124.9	124.1	125.4
13	143.3	117.9	119.1	119.2	118.2	118.2	144.1	143.1	143.6	144.2
14	42.4	46.4	46.4	46.4	42.9	42.9	47.7	42.2	41.5	44.2
15	26.6	68.0	68.1	68.1	27.1	27.2	68.2	26.7	27.6	35.8
16	27.9	38.0	38.8	38.7	27.6	27.6	38.8	23.7	28.1	66.7
17	33.8	33.6	34.1	34.1	33.3	33.3	33.9	38.6	33.7	38.5
18	58.8	42.2	47.5	47.5	47.7	47.7	58.7	53.8	58.6	60.3
19	39.3	41.3	40.5	40.5	40.8	40.8	39.3	39.8	39.4	39.4
20	39.5	71.5	39.3	39.3	39.5	39.5	39.1	39.4	39.6	39.2
21	31.1	35.5	31.0	31.0	31.2	31.3	30.9	31.1	31.2	30.4
22	41.3	35.9	41.2	41.2	41.6	41.6	41.0	36.1	42.1	35.0
23	26.9	28.3	22.7	28.4	22.7	28.4	28.2	28.8	28.3	26.0
24	21.4	15.8	64.4	15.8	64.5	15.8	15.6	16.6	15.7	21.4
25	16.6	16.3	16.8	16.2	16.8	16.2	17.1	17.4	17.1	18.0
26	18.2	18.7	18.6	18.7	18.0	18.1	18.9	18.3	18.3	16.2
27	22.5	17.7	17.8	17.8	23.8	23.9	16.6	22.7	22.6	23.2
28	28.7	28.7	29.0	29.0	28.5	28.5	29.3	69.1	28.8	21.9
29	17.5	11.9	17.0	17.0	17.0	17.0	17.3	17.6	17.5	17.7
30	21.3	29.9	21.2	21.2	21.2	21.2	21.3	21.6	21.5	21.2
OCH_3_	54.4	52.0	51.6	51.5	51.6	51.4	54.7		53.9	
Ref	[104]	[193]	[193]	[54]	[193]	[54]	[54]	[189]	[74]	[229]
**C**	**U21**	**U22**	**U23**	**U24**	**U25**	**U26 ^b^**	**U27**	**U28**	**U29 ^a^**	**U31**
1	40.1	39.3	39.9	39.4	39.4	40.2	38.3	39.5	38.6	39.2
2	34.2	34.3	34.1	34.3	34.4	26.6	27.1	27.7	27.6	33.9
3	218.0	219.3	216.7	219.1	219.4	83.1	78.9	79.6	73.1	217.1
4	47.6	51.44	47.7	51.5	51.5	44.3	38.8	40.0	43.1	47.5
5	55.3	55.6	55.1	55.7	55.7	48.4	54.5	56.2	48.2	54.4
6	19.7	18.7	19.0	18.8	18.7	18.8	17.9	18.8	18.0	19.1
7	32.4	35.6	35.5	35.7	35.6	34.6	35.1	32.7	31.6	34.4
8	43.0	46.2	46.6	46.3	46.3	44.2	43.0	43.1	42.1	42.9
9	47.8	58.7	59.1	58.7	58.7	49.6	52.7	53.7	53.5	52.1
10	37.4	36.5	36.9	36.6	36.5	39.1	36.5	37.5	36.6	36.2
11	81.8	194.2	194.4	194.2	194.2	78.4	129.4	132.9	133.8	129.0
12	124.8	145.1	145.1	145.1	145.0	145.8	132.5	131.5	129.3	132.8
13	146.1	132.9	133.9	133.9	134.7	116.9	85.8	85.8	84.9	85.6
14	42.0	47.3	47.4	47.4	47.4	44.3	48.9	46.9	44.6	49.1
15	27.9	68.1	68.3	68.3	68.3	36.4	68.4	36.0	25.8	68.3
16	26.3	38.4	38.0	38.6	38.6	78.3	37.8	66.4	27.3	37.2
17	33.8	33.9	33.7	34.1	34.2	39.7	42.7	48.4	42.5	42.3
18	58.7	47.5	43.6	48.6	48.7	50.9	61.0	63.3	61.6	55.3
19	39.4	35.5	41.4	34.8	40.6	42.1	37.6	39.3	37.9	38.8
20	39.3	51.4	70.9	46.4	39.1	41.0	40.6	42.1	41.0	71.7
21	31.1	25.3	35.4	24.9	30.9	32.5	31.2	30.5	31.7	35.9
22	41.3	39.7	35.6	40.4	40.7	36.7	34.0	31.8	35.2	29.0
23	26.5	22.1	26.4	22.1	22.0	67.4	27.7	28.4	67.4	26.0
24	21.4	65.6	21.4	65.6	65.6	13.6	14.9	15.7	12.5	20.8
25	18.1	16.7	15.8	16.6	16.7	17.6	17.7	18.4	18.5	17.1
26	16.2	18.5	18.9	18.6	18.6	18.9	19.9	20.1	19.8	19.6
27	22.0	15.4	15.1	15.1	15.1	25.2	12.8	19.0	17.4	12.5
28	28.5	29.1	29.2	29.3	29.5	23.7	76.8	73.1	76.8	76.5
29	17.5	17.4	11.6	16.2	16.6	17.6	18.1	18.9	18.4	12.9
30	21.3	176.4	29.7	65.7	20.9	21.7	19.4	19.8	19.5	28.7
OCH_3_						53.5				
1′						105.8				
2′						75.7				
3′						78.4				
4′						71.8				
5′						77.6				
6′						62.8				
1″						106.1				
2″						75.7				
3″						78.3				
4″						71.6				
5″						77.8				
6″						62.9				
Ref	[229]	[193]	[54]	[193]	[54]	[230]	[54]	[189]	[189]	[193]
**C**	**U32**	**U33**	**U34 ^a^**	**U35**	**U36 ^a^**	**U37 ^a^**	**U38**	**U39**	**U40**	**U41**
1	40.9	40.9	39.5	47.1	48.0	47.9	38.6	39.8	39.8	33.5
2	28.2	27.4	34.2	68.6	68.7	68.8	23.8	25.9	34.2	25.3
3	76.9	78.7	217.8	84.9	85.7	85.8	81.1	NR	217.1	75.8
4	52.8	39.0	47.4	43.4	43.9	43.9	38.1	38.7	47.8	37.5
5	55.8	54.7	55.2	55.5	56.5	56.6	55.4	56.7	55.4	48.3
6	18.6	18.5	19.6	17.6	19.1	19.3	18.3	18.8	18.8	17.4
7	35.6	35.0	32.3	33.1	33.8	33.9	32.9	34.3	32.2	32.7
8	43.8	44.7	40.0	45.5	40.2	40.7	40.2	41.3	43.7	45.3
9	52.7	55.7	46.8	59.5	48.2	48.0	47.7	49.4	60.8	61.4
10	38.3	38.2	36.6	37.4	38.2	38.3	36.9	38.0	36.6	37.0
11	70.0	67.8	23.3	194.8	24.0	24.4	23.5	24.2	199.0	200.0
12	146.1	130.2	125.3	144.4	125.7	128.1	125.1	125.1	130.6	130.7
13	116.1	142.7	138.4	134.8	138.7	139.5	138.9	140.8	164.8	164.3
14	45.9	47.0	42.2	41.7	42.4	42.1	42.2	43.3	45.5	43.7
15	72.2	72.1	25.9	27.5	29.3	29.9	26.1	26.7	27.2	27.1
16	34.2	34.5	23.3	27.3	24.4	25.9	23.7	24.1	27.4	27.5
17	34.0	33.9	37.9	33.4	48.3	48.6	38.1	37.7	33.8	33.8
18	47.4	58.3	54.0	48.9	53.3	54.5	54.2	55.3	58.8	58.8
19	40.6	39.1	33.7	40.8	39.4	72.7	39.5	41.4	33.5	33.5
20	39.3	39.2	46.7	39.3	39.2	42.2	39.5	40.7	46.5	46.5
21	30.9	30.8	24.5	31.1	30.8	26.8	30.7	32.2	24.8	24.6
22	40.7	40.7	34.7	41.1	36.4	37.5	35.3	36.5	40.5	40.5
23	18.7	28.2	26.4	23.0	24.2	24.2	28.2	28.3	28.8	28.4
24	207.6	15.6	21.5	65.3	65.7	65.7	17.0	17.5	22.1	22.3
25	15.7	16.8	15.5	18.3	17.5	17.4	15.8	16.3	16.0	16.4
26	19.2	18.6	16.7	18.5	17.4	17.2	16.8	17.3	18.4	18.5
27	19.5	18.7	23.3	20.9	23.9	24.6	23.4	23.9	20.5	20.7
28	28.9	28.9	69.6	28.8	176.3	177.1	69.9	70.5	28.7	28.7
29	16.8	17.5	16.9	16.6	17.5	27.1	17.5	17.8	17.0	17.0
30	21.0	21.2	66.3	21.0	21.4	16.7	21.4	21.6	65.9	65.9
OCOPh	165.5	165.6								
*iso*	131.0	131.0								
*orto*	129.5	129.5								
*meta*	128.8	128.4								
*para*	132.9	132.8								
1′					93.7	93.8	127.2	127.3		
2′					79.2	79.1	114.0	129.5		
3′					78.9	79.1	144.8	115.8		
4′					70.7	70.8	147.2	157.4		
5′					79.2	79.2	115.3	115.8		
6′					62.1	62.2	122.1	129.5		
7′							150.0	143.8		
8′							115.7	116.4		
9′							167.9	167.2		
1″					104.8	104.7				
2″					76.0	75.9				
3″					78.4	78.4				
4″					72.7	72.9				
5″					78.3	78.3				
6″					63.7	63.9				
Ref	[54]	[54]	[185]	[193]	[194]	[194]	[231]	[204]	[185]	[185]
**C**	**U42**	**U43**	**U44**	**U45**	**U46**	**U47**	**U48**	**U49**	**U50**	**U51 ^a^**
1	40.5	40.7	41.7	38.7	38.5	38.7	39.0	39.3	38.9	39.8
2	28.2	28.2	28.3	27.0	27.8	27.0	27.6	27.2	27.6	28.1
3	76.9	76.9	77.9	78.6	76.9	78.6	80.5	78.7	80.6	77.9
4	52.8	52.8	49.3	38.8	52.7	38.8	43.0	39.0	43.1	39.8
5	56.2	56.2	56.3	55.1	56.2	55.1	55.2	54.6	55.5	55.3
6	18.6	18.5	20.2	18.6	18.7	18.6	17.8	17.7	17.6	18.0
7	37.1	37.2	37.3	39.1	39.0	39.2	36.4	36.2	33.2	33.2
8	43.6	43.7	43.8	38.2	38.3	38.2	46.7	46.7	45.5	45.7
9	52.6	52.9	53.3	58.0	56.7	57.9	59.7	59.8	59.7	61.3
10	38.2	38.3	38.4	38.1	38.3	38.1	37.0	37.3	36.9	37.4
11	69.7	70.1	70.2	72.8	73.1	73.0	194.9	195.1	195.1	199.3
12	146.5	145.7	145.7	207.1	206.7	207.1	144.9	144.9	144.5	130.7
13	115.1	116.7	116.6	42.7	42.9	43.0	134.2	134.0	134.4	163.6
14	46.6	46.6	46.7	49.9	49.3	49.5	47.2	47.2	41.7	45.8
15	68.0	68.1	68.1	66.0	66.1	66.2	68.3	68.4	27.5	37.1
16	38.1	38.9	38.8	37.2	38.0	37.8	38.6	38.5	27.3	64.7
17	33.6	34.2	34.1	32.6	33.2	33.1	34.2	34.2	33.4	39.2
18	42.0	47.4	47.3	35.3	39.6	39.5	48.7	48.7	48.9	60.7
19	41.3	40.5	40.5	41.4	40.5	40.5	40.4	40.4	40.8	39.1
20	71.6	39.3	39.3	71.3	37.9	38.0	39.1	39.1	39.3	39.4
21	35.6	31.0	31.0	35.2	30.8	30.8	30.9	30.9	31.2	30.8
22	35.7	41.0	41.0	36.0	41.5	41.5	40.7	40.7	41.1	35.4
23	19.3	19.3	23.8	27.8	19.2	27.9	22.4	28.0	22.4	28.7
24	207.8	207.9	178.4	15.3	207.1	15.2	64.3	15.6	64.3	16.6
25	15.8	15.8	14.4	17.3	16.2	17.3	17.1	16.6	17.1	17.0
26	18.7	18.6	18.5	21.2	20.9	21.1	19.0	19.1	18.4	18.7
27	17.7	18.0	17.8	18.1	17.7	17.9	15.2	15.3	20.9	21.9
28	29.0	29.3	29.3	27.9	28.2	28.2	29.4	29.4	28.8	23.0
29	11.4	16.8	16.7	11.7	16.6	16.6	16.6	16.5	16.5	17.6
30	29.9	21.1	21.0	29.1	20.5	20.5	20.9	20.9	21.0	21.2
OCH_3_			51.3							
Ref	[193]	[54]	[54]	[193]	[193]	[193]	[193]	[54]	[54]	[190]
**C**	**U52 ^a^**	**U54**	**U55**	**U56**	**U57 ^c^**	**U58**	**U59**	**U60**	**U62**	**U63 ^a^**
1	38.5	39.2	38.4	38.1	37.9	38.6	38.5	38.1	38.5	40.5
2	27.6	27.3	27.5	23.4	28.7	23.8	23.7	26.8	23.6	36.1
3	72.8	78.8	78.0	80.7	80.0	81.2	80.6	78.5	80.6	216.0
4	43.1	39.1	42.3	37.9	42.8	37.9	37.8	38.7	37.7	54.7
5	48.1	54.8	54.6	55.0	52.5	55.5	55.3	54.6	55.3	49.2
6	17.9	17.5	19.3	17.7	18.5	18.4	18.3	17.5	18.2	67.8
7	33.0	32.8	33.3	31.4	33.3	33.1	32.9	31.0	32.9	41.6
8	42.1	45.1	40.9	41.8	39.9	40.2	40.1	41.5	40.0	42.6
9	48.9	61.6	51.1	53.0	48.7	47.8	47.7	52.8	47.6	46.6
10	37.0	36.9	31.0	36.4	37.1	37.0	36.8	36.1	36.8	37.8
11	37.4	199.8	73.3	133.4	24.4	23.6	23.4	128.6	23.4	77.3
12	209.8	130.7	126.0	129.0	128.0	124.5	124.4	133.2	124.3	145.0
13	89.4	164.3	140.4	89.7	140.2	139.8	139.7	89.5	139.6	116.5
14	45.9	43.6	42.5	42.0	41.7	42.4	42.1	41.7	42.1	41.4
15	26.3	27.1	28.4	25.6	30.6	28.3	26.6	25.3	26.6	27.6
16	26.4	27.5	26.0	30.9	27.6	26.8	28.1	22.6	28.1	28.0
17	42.7	33.8	47.8	45.2	47.0	33.9	33.8	44.9	33.7	33.7
18	55.0	58.8	53.5	40.4	54.6	59.3	59.1	60.4	59.1	47.5
19	38.0	33.5	72.1	38.2	72.4	39.8	39.6	37.9	39.6	41.3
20	40.7	46.5	42.5	60.7	39.7	39.8	39.7	40.1	39.7	40.0
21	31.6	24.9	27.4	22.9	26.8	31.5	31.3	30.6	31.2	31.6
22	34.9	40.5	38.3	31.3	38.5	41.7	41.6	31.1	41.5	42.2
23	67.2	28.0	180.6	27.8	68.4	28.3	28.1	27.6	28.1	66.8
24	12.9	22.1	13.7	16.1	12.8	16.9	16.8	14.7	16.8	20.5
25	16.1	16.5	16.7	19.2	17.0	16.0	15.7	17.7	15.7	17.6
26	18.7	18.5	16.9	19.0	16.8	17.7	16.9	18.7	16.9	20.3
27	17.7	20.6	23.9	16.2	25.0	23.4	23.3	15.9	23.2	24.0
28	77.2	28.7	68.7	18.1	64.5	29.1	28.8	179.6	28.7	28.9
29	19.1	17.0	17.4	17.9	27.1	17.0	17.5	17.6	17.5	17.4
30	19.8	65.9	28.6	179.9	16.2	21.6	21.4	18.9	21.4	21.5
OCH_3_			55.4							51.4
OCH_3_			21.7	21.4	21.2	21.5				
C=O			170.4	171.1	169.9	171.5	173.5		173.7	
2′							34.9		34.9	
3′							25.2		25.2	
4′–13′							29.2–29.7		29.2–29.5	
14′							29.7		31.9	
15′							29.5		22.7	
16′							31.9		14.1	
17′							22.7			
18′							14.1			
Ref	[189]	[185]	[232]	[233]	[232]	[234]	[235]	[236]	[6]	[190]
**C**	**U64 ^c^**	**U65**	**U66 ^a^**	**U67**	**U68**	**U69 ^a^**	**U71**	**U72**	**U73^c^**	**U74**
1	38.9	43.0	39.4	39.5	39.5	48.0	39.7	33.6	34.1	38.4
2	29.3	175.5	34.8	34.2	34.2	68.6	34.1	25.8	25.7	26.8
3	208.1	207.7	216.2	217.9	217.8	83.7	217.3	74.2	76.3	77.4
4	47.2	50.7	47.4	47.5	47.5	39.9	47.7	37.4	42.6	38.4
5	55.6	47.7	55.2	55.3	55.3	55.9	55.2	48.1	54.1	54.8
6	19.8	20.4	19.8	19.7	19.7	18.9	18.6	17.1	19.0	18.2
7	34.0	32.1	32.5	32.3	32.4	33.5	32.4	32.9	33.2	32.3
8	40.2	40.0	40.3	40.2	40.1	40.1	44.5	44.6	41.3	39.6
9	46.6	40.4	47.1	46.9	46.9	47.0	60.7	61.2	48.1	47.2
10	37.5	42.3	36.7	36.7	36.7	38.5	36.1	37.2	37.4	38.4
11	24.5	23.8	23.8	23.7	23.7	23.8	199.5	199.3	73.1	23.0
12	127.9	125.1	125.2	125.5	125.8	125.5	130.7	130.3	127.3	124.8
13	138.8	139.1	139.3	138.0	137.9	139.3	163.5	163.3	139.4	138.0
14	42.0	42.7	43.1	42.6	42.2	42.6	43.8	43.6	43.1	42.2
15	28.7	26.5	26.5	26.3	25.9	28.7	28.5	28.3	27.7	25.5
16	26.6	29.2	21.4	27.5	23.3	24.9	23.7	23.9	25.9	20.3
17	48.4	35.2	39.7	38.0	37.9	48.1	47.7	47.0	48.9	36.4
18	54.3	58.6	58.3	52.8	53.0	53.6	52.8	52.8	54.5	57.2
19	73.2	38.4	34.8	34.0	34.1	39.4	38.6	38.1	71.9	33.6
20	41.8	47.1	51.2	45.1	51.8	39.5	38.6	38.4	44.0	49.7
21	27.0	71.8	34.4	32.4	25.1	31.0	30.3	30.1	27.2	33.1
22	39.1	50.3	77.6	74.8	34.3	37.5	36.7	36.0	38.6	76.7
23	23.5	23.9	26.7	26.7	26.7	29.4	26.6	28.8	177.4	28.0
24	181.1	19.5 *	21.6	21.6	21.6	17.7	21.3	22.1	13.1	15.3
25	15.0	19.4 *	15.4	15.5	15.5	17.0	15.5	16.0	16.4	15.7
26	17.0	16.9	16.9	16.8	16.8	17.5	19.0	18.7	17.7	16.4
27	25.3	22.9	23.8	23.8	23.5	23.9	20.9	20.4	25.2	23.1
28	65.4	28.4	25.2	21.7	69.3	179.9	180.0	180.0	179.7	24.3
29	26.9	17.4	19.0	18.5	18.0	17.5	17.0	16.5	27.7	17.9
30	16.8	15.8	178.2	182.1	181.6	21.4	21.0	20.4	17.9	177.0
Ref	[232]	[54]	[129]	[61]	[61]	[157]	[185]	[185]	[232]	[237]
**C**	**U75**	**U76**	**U77 ^d^**	**U78 ^a^**	**U79**	**U80**	**U81**	**U82**	**U83 ^a^**	**U84**
1	42.7	38.1	39.0	48.0	40.0	40.9	40.4	40.8	33.9	38.8
2	34.5	23.8	28.3	68.6	34.4	27.3	27.8	27.4	28.4	27.3
3	74.6	80.8	79.1	83.9	NR	78.5	80.6	78.7	78.8	79.0
4	45.4	37.5	39.2	39.8	47.9	39.0	43.0	39.0	38.9	38.8
5	49.9	55.1	55.5	56.1	55.6	54.8	55.8	54.9	55.4	55.4
6	19.3	18.0	18.6	19.0	19.0	18.5	18.4	18.5	18.1	18.4
7	66.9	32.7	33.3	33.6	32.4	37.0	33.9	36.7	33.2	32.9
8	40.9	39.3	38.9	40.7	43.9	44.1	42.7	44.6	40.3	39.4
9	48.9	47.3	47.8	47.9	60.9	54.4	54.4	55.7	48.6	47.8
10	39.1	36.9	37.1	38.5	36.8	38.2	37.8	38.2	41.4	37.2
11	24.6	23.4	23.6	24.2	199.2	69.9	70.1	68.1	25.2	23.4
12	126.7	125.5	125.8	128.2	130.5	145.7	144.7	129.8	125.7	125.0
13	139.7	137.8	138.5	139.5	165.5	117.0	115.8	143.5	138.9	138.0
14	43.3	41.6	42.3	42.1	45.1	46.5	40.9	47.7	42.3	42.8
15	29.2	27.9	27.0	29.8	29.8	68.2	26.9	68.0	26.7	29.2
16	25.3	23.9	24.5	26.8	27.4	38.7	27.6	38.8	28.2	22.6
17	48.6	47.8	48.1	48.6	34.3	34.1	33.2	34.0	33.7	36.8
18	54.4	52.3	53.1	54.5	59.2	47.3	47.7	58.3	59.0	54.1
19	40.4	38.8	39.4	72.7	39.4	40.5	40.8	39.1	34.1	38.9
20	40.4	38.7	39.7	42.2	39.4	39.3	39.5	39.2	47.3	39.4
21	31.8	30.4	30.9	25.9	31.0	31.0	31.2	30.9	25.3	30.7
22	38.1	36.5	37.2	37.5	41.0	41.1	41.5	40.9	41.2	30.6
23	23.1	27.9	28.3	29.4	26.5	28.2	22.6	28.2	29.0	28.1
24	65.9	16.9	17.1	17.6	21.6	15.6	64.4	15.6	16.1	15.4
25	17.4	15.5	15.7	17.4	15.9	16.7	17.3	16.8	61.0	15.6
26	21.6	16.5	15.9	16.7	18.5	18.6	17.9	18.6	17.4	16.9
27	24.1	23.9	23.8	24.7	20.6	18.2	24.1	17.2	23.7	23.4
28	181.6	184.0	181.1	177.0	29.0	29.3	28.7	29.3	28.7	69.7
29	17.6	16.8	17.3	27.0	17.6	16.8	16.7	17.5	17.1	16.2
30	17.7	21.0	21.5	17.0	21.3	21.1	21.1	21.3	65.9	21.3
1′				93.7						
2′				79.3						
3′				79.0						
4′				70.9						
5′				79.1						
6′				62.4						
1″				104.8						
2″				75.9						
3″				78.3						
4″				72.9						
5″				78.1						
6″				63.9						
Ref	[238]	[239]	[240]	[241]	[242]	[54]	[193]	[54]	[243]	[244]
**C**	**U85**	**U86**	**U88**							
1	38.8	37.7	36.8							
2	27.4	27.1	25.2							
3	79.0	79.1	78.2							
4	38.8	38.7	38.4							
5	55.3	55.4	50.7							
6	18.4	18.8	17.9							
7	34.6	41.8	31.6							
8	41.0	38.9	40.2							
9	50.5	49.2	154.0							
10	37.2	37.9	38.2							
11	22.2	17.4	114.9							
12	24.7	32.2	122.5							
13	135.4	40.0	140.7							
14	45.0	159.2	42.6							
15	26.8	116.3	27.8							
16	38.9	40.6	25.7							
17	34.0	33.9	33.2							
18	136.4	60.4	56.8							
19	36.7	35.4	38.5							
20	35.0	36.5	38.9							
21	23.7	28.5	30.7							
22	36.3	38.5	40.9							
23	28.1	28.0	28.2							
24	15.5	15.5	16.9							
25	16.3	15.2	17.1							
26	17.8	26.3	21.6							
27	21.9	19.4	24.9							
28	28.3	37.0	29.2							
29	23.1	27.5	17.1							
30	20.5	22.5	21.0							
Ref	[245]	[245]	[246]							

Ref: References; * Values bearing the same superscript are interchangeable; NR: Not reported; Solvent CDCl_3_; ^a^ Pyridine-d_5_; ^b^ CD_3_OD; ^c^ CDCl_3_ + DMSO-d_6_; ^d^ CDCl_3_+CD_3_OD; ^13^C-NMR data of some compounds were not found. In these cases, the reported identification were performed by comparison of other physical data: U30, U53 [247], U70 (^1^H -NMR, IR, MS) [248] e U87 (m.p., [α]_D_, IR, ^1^H-NMR) [249].

**Table 8:** ^13^C-NMR data of pentacyclic triterpenoids classified as others isolated from Celastraceae species (2001–2021).

C	OT3	OT4	OT5	OT6	OT7	OT8	OT9 ^a^	OT10 ^a^	OT11 ^a^	OT12
1	39.6	38.9	104.6	18.2	37.8	37.2	104.5	106.2	121.3	36.6
2	34.2	27.5	144.7	27.8	27.1	194.7	147.5	146.9	182.0	34.8
3	218.2	78.4	144.2	76.3	79.0	200.1	144.2	144.4	182.2	217.1
4	47.4	39.0	118.0	40.8	38.7	123.8	118.1	128.7	131.4	47.6
5	54.9	55.3	129.8	141.6	55.5	154.8	129.8	117.6	142.1	53.2
6	19.7	18.5	122.5	122.0	18.8	75.0	122.6	121.6	121.3	26.3
7	33.7	33.4	127.9	23.5	41.3	122.9	127.9	130.0	153.2	22.6
8	41.6	41.7	131.3	47.8	38.9	154.0	131.3	141.7	47.6	41.0
9	49.6	50.4	135.6	34.8	49.2	51.6	135.3	132.2	144.8	147.4
10	36.8	37.2	129.5	49.6	38.0	71.6	129.5	131.8	158.2	39.3
11	21.6	21.1	25.5	34.6	17.5	28.2	29.3	129.0	128.3	115.6
12	23.9	24.0	32.8	30.5	37.7	29.5	35.9	138.1	38.9	36.1
13	49.6	49.5	102.5	37.7	36.0	38.2	54.5	44.1	41.6	36.7
14	42.1	42.1	43.6	39.5	158.0	40.3	156.8	49.0	43.2	38.2
15	32.6	33.7	38.8	32.5	116.9	28.5	34.1	31.3	24.8	29.6
16	21.6	21.7	40.2	35.7	33.6	35.6	41.4	38.8	38.6	35.8
17	54.9	54.9	42.6	30.5	37.6	30.7	41.6	32.4	33.2	42.8
18	44.7	44.8	46.0	41.9	48.0	43.4	50.4	43.2	48.2	52.0
19	41.9	41.9	32.8	39.3	31.1	30.6	36.8	32.7	32.6	20.1
20	27.3	27.4	40.2	33.1	33.8	41.7	41.9	41.9	41.7	28.2
21	46.4	46.5	36.8	29.5	28.1	29.9	30.8	31.0	31.1	59.6
22	148.6	148.6	36.9	27.9	35.3	35.9	35.5	36.2	34.5	30.7
23	26.6	28.2	11.6	28.9	28.0	9.5	11.6	11.6	11.2	22.0
24	21.1	15.7		25.4	15.5					25.5
25	15.7	16.7	20.5	16.1	15.4	27.0	20.7	19.5	27.7	21.6
26	16.4	15.9	23.2	18.1	25.9	21.5	28.0	20.1	20.5	16.9
27	16.6	16.7	23.2	20.4	21.1	28.7	108.3	23.4	20.5	15.3
28	16.1	16.1	25.1	32.0	29.9	31.7	31.3	31.7	31.8	13.9
29	110.1	110.2	180.5	74.4	73.9	178.8	184.7	183.6	183.2	22.1
30	25.0	25.0	26.3	26.0	24.6	32.5	25.9	32.5	34.0	23.0
Ref	[94]	[94]	[139]	[213]	[213]	[250]	[139]	[139]	[139]	[251]
**C**	**OT13**	**OT14**	**OT15**	**OT17**	**OT18**	**OT19**	**OT20**	**OT21**		
1	110.7	110.6	98.9	38.5	18.2	23.6	36.1	38.1		
2	163.7	163.7	143.2	27.4	27.8	18.1	27.8	27.3		
3			178.1	79.0	76.3	76.2	79.0	79.2		
4	24.7	28.2	40.1	39.0	40.8	39.2	39.6	39.1		
5	103.7	105.6	43.3	55.7	141.7	141.6	52.3	55.7		
6	126.4	125.8	28.2	18.3	122.0	121.9	21.4	19.0		
7	115.9	115.9	17.3	34.7	23.8	27.7	26.7	35.3		
8	161.1	160.4	47.7	40.8	45.7	43.0	41.0	38.9		
9	39.6	39.5	38.8	51.3	34.8	34.8	148.9	48.9		
10	165.2	166.1	47.8	37.3	49.7	46.6	39.1	37.9		
11	33.4	33.3	33.9	21.2	29.9	34.6	114.3	17.7		
12	29.7	29.7	28.7	26.2	29.7	30.3	36.0	35.9		
13	40.5	40.3	38.9	39.0	38.4	37.7	36.8	37.9		
14	44.1	44.1	37.6	43.4	38.6	40.7	38.2	158.1		
15	28.4	28.4	28.8	27.6	34.3	32.0	29.7	117.0		
16	35.5	35.5	35.9	37.7	36.3	35.9	35.9	36.9		
17	38.2	38.2	30.1	34.4	32.9	30.0	43.0	38.1		
18	43.4	43.4	44.2	142.8	44.7	47.4	52.1	49.4		
19	32.0	32.1	30.2	129.8	35.8	35.1	20.2	41.4		
20	41.9	41.9	40.3	32.3	34.5	28.2	28.2	29.0		
21	213.8	213.7	29.9	33.4	74.7	33.0	59.6	33.9		
22	52.5	52.5	36.2	37.4	46.3	38.9	30.8	33.2		
23			7.9	28.0	25.4	28.9	28.2	28.1		
24			99.4	15.4	28.9	25.4	15.6	15.6		
25	36.8	36.5	17.5	16.1	16.6	16.2	22.1	15.6		
26	22.5	22.5	15.9	16.7	17.0	18.4	17.0	30.1		
27	19.7	19.8	17.2	14.6	19.1	19.6	15.3	26.0		
28	32.5	32.6	31.6	25.3	33.1	32.4	14.0	30.1		
29			179.0	31.3	32.3	34.6	23.0	33.5		
30	15.1	15.1	31.8	29.2	24.6	32.0	22.2	21.5		
OCH_3_	50.5	51.0	51.3							
Ref	[252]	[252]	[196]	[220]	[196]	[157]	[253]	[254]		

Ref: References; NR: Not reported; Solvent CDCl_3_; ^a^ CD_3_OD; ^13^C-NMR data of some compounds were not found. In these cases, the reported identification was performed by comparison of other physical data: OT1 (m.p., [α]_D_, IR, ^1^H-NMR) [255], OT2 e OT16 (m.p., [α]_D_, IR, ^1^H-NMR, MS) [256].

## Data Availability

The information about the PCTTs was obtained from SciFinder, Scopus, and Web of Science, using as key search terms: “Celastraceae and triterpenes”, “Celastraceae and com-pounds”, “Celastraceae and phytochemistry” and “Celastraceae and metabolites”.

## Supplementary Materials

The following are available online, Table S1: Pentacyclic triterpenoids isolated from Celastraceae species (2001–2021) [257,258,259,260,261,262,263,264,265,266,267,268,269,270,271,272,273,274,275,276,277,278,279,280,281,282,283,284,285,286,287,288,289,290,291,292,293,294,295,296,297,298,299,300,301,302,303,304,305,306,307,308,309,310,311,312,313,314,315,316,317,318,319,320].
