# Supplementary material for: Pentacyclic Triterpenoids Isolated from Celastraceae: A Focus in the 13C-NMR Data
Source: Molecules. 2022 Jan 31;27(3):959. doi: 10.3390/molecules27030959 (PMC8838773; doi:10.3390/molecules27030959)
Supplement: Supplementary file 1 [file molecules-27-00959-s001.zip › molecules-1547384-supplementary.pdf]

Tabela S1: Pentacyclic triterpenoids isolated from Celastraceae species (2001-2021).

	Name	Species (plant part) Reference
		**FRIEDELANES**
**F1**	11*β*-Hydroxy-friedelan-3-one	*Celastrus monospermus* (roots)[35]
		Maytenus robusta (leaves)[87]
**F2**	12*α*,29-Dihydroxy-friedelan-3-one	*Maytenus gonoclada* (branches)[36,37]
		Maytenus robusta (branches)[45]
**F3**	12*α*-Hydroxy-friedelan-3,16-dione	*Maytenus gonoclada* (branches, leaves, stems, roots)[37,257]
		Maytenus robusta (branches)[46]
**F4**	12*α*-Hydroxy-friedelan-3-one	*Celastrus monospermus* (roots)[35]
		Maytenus distichophylla (leaves)[72]
		Maytenus gonoclada (leaves, branches, stems, roots)[36–38,257]
**F5**	12*β*-Hydroxy-friedelan-3-one	*Salacia chinensis* (leaves)[258]
**F6**	15*α*,26-Dihydroxy-friedelan-3-one (Salasone D)	*Salacia chinensis* (stems)[39]
**F7**	15*α*-Hydroxy-friedelan-3-one	*Salacia amplifolia* (roots)[259]
		Salacia chinensis (stems)[39]
**F8**	16*β*-Acetyloxy-26-hydroxy-friedelan-1,3-dione (Kotalagenin 16-acetate)	*Salacia reticulata* (roots)[260]
**F9**	16*α*,28-Dihydroxy-friedelan-3-one (Celasdin B)	*Salacia elliptica* (leaves, branches)[43,44]
**F10**	16*α*-Hydroxy-friedelan-1,3-dione	*Salacia elliptica* (branches)[43]
**F11**	16*α*-Hydroxy-friedelan-3-one	*Salacia elliptica* (branches)[44]
**F12**	16*β*,28,29-Trihydroxy-friedelan-3-one	*Maytenus robusta* (branches)[45]
**F13**	16*β*,29-Dihydroxy-friedelan-3-one	*Maytenus robusta* (branches)[46]
**F14**	16*β*-Hydroxy-friedelan-3-one	*Maytenus guianensis* (stem barks, bark)[261,262]
**F15**	1*α*,29-Dihydroxy-friedelan-3-one	*Maytenus robusta* (branches)[45]
**F16**	1*β*,25-Dihydroxy-friedelan-3-one	*Crossopetalum lobatum* (leaves)[47]
**F17**	1*β*,30-Dihydroxy-friedelan-3-one	*Celastrus vulcanicola* (root barks)[48]
**F18**	1*β*-Hydroxy-friedelan-3-one	*Celastrus vulcanicola* (root barks)[48]
**F19**	21*α*-Hydroxy-friedelan-1,3-dione	*Salacia verrucosa* (stems)[49]
**F20**	21*α*-Hydroxy-friedelan-3-one	*Cheiloclinium cognatum* (leaves)[69]
		Hippocratea excelsa (root barks)[96]
		Salacia nitida (leaves)[263]
		Salacia verrucosa (stems)[49]
		Tontelea micrantha (branches)[52]
**F21**	21*α*-Hydroxy-friedelan-3,15-dione	*Maytenus robusta* (aerial parts)[51,264]
**F22**	21*β*-Hydroxy-friedelan-3-one	*Siphonodon celastrineus* (stems)[54]
		Tontelea micrantha (branches)[52]
**F23**	25,26-Oxido-friedelan-1,3-dione	*Salacia chinensis* (roots)[53]
**F24**	25-Hydroxy-friedelan-3-one	*Maytenus jelskii* (root barks)[48]
**F25**	25-Hydroxy-friedelan-3,21-dione	*Siphonodon celastrineus* (stems)[54]
**F26**	26-Hydroxy-friedelan-1,3-dione	*Salacia reticulata* (roots)[260]
		Salacia verrucosa (stems)[49]
**F27**	27-Hydroxy-friedelan-3-one	*Salacia verrucosa* (stems)[49]

Tabela S1: Pentacyclic triterpenoids isolated from Celastraceae species (2001-2021).

	Name	Species (plant part) Reference
**F28**	28,29-Dihydroxy-friedelan-3-one	*Maytenus jelskii* (root barks)[48]
		Salacia crassifolia (leaves)[6,265]
**F29**	28,30-Dihydroxy-friedelan-3-one	*Celastrus vulcanicola* (root barks)[48]
	(Maytenfoliol)	*Maytenus jelskii* (root barks)[48]
**F30**	28-Hydroxy-friedelan-1,3-dione	*Crossopetalum lobatum* (leaves)[47]
		Cheiloclinium hippocratioides (root barks)[138]
**F31**	28-Hydroxy-friedelan-3-one	*Austroplenckia populnea* (leaves)[266]
	(Canophyllol)	*Celastrus monospermus* (roots)[35]
		Celastrus vulcanicola (stems)[267]
		Cheiloclinium cognatum (leaves, branches)[69,268]
		Elaeodendron buchananii (stem barks)[232]
		Maytenus jelskii (root barks)[48]
		Salacia crassifolia (leaves)[6]
		Salacia elliptica (leaves, branches)[43,44]
		Tontelea micrantha (leaves)[52]
**F32**	29-Hydroxy-3-oxo-friedelan-28-al	*Maytenus jelskii* (root barks)[48]
	(Tripterfrielanon A)	*Tripterygium wilfordii* (roots)[92]
**F33**	29-Hydroxy-friedelan-1,3-dione	*Crossopetalum lobatum* (leaves)[47]
**F34**	29-Hydroxy-friedelan-3,16-dione	*Maytenus robusta* (branches)[46]
**F35**	29-Hydroxy-friedelan-3-one	*Celastrus vulcanicola* (root barks, stems)[48,267]
		Celastrus hypoleucus (aerial parts)[198]
		Cheiloclinium cognatum (leaves, branches)[69,268]
		Cheiloclinium hippocratioides (root barks)[138]
		Euonymus alatus (root barks)[269]
		Hippocratea excelsa (root barks)[213]
		Maytenus cuzcoina (root barks)[68]
		Maytenus distichophylla (leaves)[72]
		Maytenus guianensis (bark, stems, branches)[261,262]
		Maytenus imbricata (roots)[123]
		Maytenus jelskii (root barks)[48]
		Maytenus robusta (leaves)[46,87]
		Maytenus salicifolia (root barks)[105]
		Microtropis triflora (caules)[270]
		Mortonia greggii (roots, leaves)[271]
		Salacia chinensis (leaves, stems)[39,258]
		Salacia crassifolia (roots)[75]
		Salacia nitida (leaves)[263]
		Salacia petenensis (bark)[64]
		Tripterygium wilfordii (roots)[92,272]
**F36**	2α-Acetyloxy-3β,24-oxido-3α-hydroxy-friedelan-29-oic acid methyl ester	*Celastrus vulcanicola* (root barks)[48]

Tabela S1: Pentacyclic triterpenoids isolated from Celastraceae species (2001-2021).

	Name	Species (plant part) Reference
**F37**	2*α*-Hydroxy-friedelan-3-one	*Maytenus jelskii* (root barks)[48]
**F38**	2*β*,22*β*-Dihydroxy-3,21-dioxo-29-*nor*-friedelan 24-oic acid methyl ester	*Tripterygium wilfordii* (roots)[61]
**F39**	2*β*-Hydroxy-24,29-*dinor*-friedelan-4-en-3,21-dione (Triptocalline B)	*Microtropis triflora* (stalks)[62,270]
**F40**	25-Benzoyloxyfriedelan-3,21-dione	*Siphonodon celastrineus* (stems)[54]
**F41**	30-Hydroxy-friedelan-1,3-dione	*Salacia verrucosa* (stems)[49]
**F42**	30-Hydroxy-friedelan-3-one (Octandronol)	*Euonymus alatus* (root barks)[269]<br>*Maytenus distichophylla* (leaves)[72]<br>*Maytenus erythroxylon* (aerial parts)[273]<br>*Maytenus salicifolia* (roots)[9]<br>*Salacia chinensis* (leaves)[258]<br>*Salacia elliptica* (leaves, branches)[43,44]<br>*Salacia hainanensis* (roots)[73]<br>*Salacia nitida* (leaves)[263]<br>*Tontelea micrantha* (branches)[52]
**F43**	24-Formyl-3-hydroxy-2-oxo-friedelan-3-en-29-oic acid methyl ester (Cangoronine methyl ester)	*Elaeodendron schlechteranum* (root barks)[63]<br>*Cassine xylocarpa* (root barks)[196]<br>*Celastrus vulcanicola* (root barks)[48]
**F44**	3-Methoxy-friedel-2-en-1-one	*Salacia petenensis* (bark)[64]
**F45**	3-Oxo-friedelan-28,30-olide	*Maytenus jelskii* (root barks)[48]
**F46**	3-Oxo-friedelan-29-al	*Maytenus jelskii* (root barks)[48]
**F47**	3-Oxo-friedelan-28-al (Canophyllal)	*Tripterygium wilfordii* (roots)[92]<br>*Tontelea micrantha* (branches)[52]
**F48**	3-Oxo-friedelan-30-al	*Celastrus vulcanicola* (root barks)[48]<br>*Maytenus jelskii* (root barks)[48]<br>*Mortonia greggii* (leaves)[271]
**F49**	3-Oxo-friedelan-29-oic acid methyl ester (Methyl populnoate)	*Austroplenckia populnea* (wood barks)[274]<br>*Mortonia greggii* (roots, leaves)[271]
**F50**	3*α*,25-Dihydroxy-friedelan-2-one	*Crossopetalum lobatum* (leaves)[47]
**F51**	3*α*-Hydroxy-friedelan-2-one	*Maytenus cuzcoina* (root barks)[68]<br>*Maytenus jelskii* (root barks)[48]
**F52**	3*β*,24-Oxido-2*α*,3*α*,6*β*-trihydroxy-fridelano-29-oic methyl ester	*Maytenus cuzcoina* (root barks)[68]<br>*Maytenus jelskii* (root barks)[48]
**F53**	3*β*,24-Oxido-2*α*,3*α*-dihydroxy-fridelano-29-oic methyl ester	*Celastrus vulcanicola* (root barks)[48]
**F54**	3*β*,29-Dihydroxy-friedelan-1-one	*Cheiloclinium cognatum* (leaves)[69]
**F55**	4*α*-methyl-friedelan-3-one (4-*epi*-friedelin)	*Salacia chinensis* (leaves)[258]

Tabela S1: Pentacyclic triterpenoids isolated from Celastraceae species (2001-2021).

	Name	Species (plant part) Reference
F56	2*β*,6*α*,22*β*-Trihydroxy-24,29-*dinor*-friedelan-4-en-3,21-dione (6*α*-Hidroxi-triptocalina A)	*Tripterygium wilfordii* (roots)[71]
F57	6*β*,12*α*-Dihydroxy-friedel-1-en-3,16,21-trione (Maitensifolone)	*Maytenus distichophylla* (leaves)[72]
F58	7*α*-Hydroxi-friedelan-1,3-dione	*Salacia nitida* (lianas)[263]
F59	7*α*,26-Dihydroxy-friedelan-3-one (Salasone E)	*Salacia chinensis* (stems)[39]
F60	7*α*,30-Dihydroxy-friedelan-3-one	*Salacia hainanensis* (roots)[73]
F61	7*β*,29-Dihydroxy-friedelan-3-one	*Celastrus vulcanicola* (root barks)[48]
F62	7*β*-Hydroxy-3-oxo-friedelan-28-oic acid methyl ester	*Celastrus vulcanicola* (root barks)[48]
F63	1*β*-Hydroxy-3-oxo-friedelan-30-oic acid	*Celastrus vulcanicola* (root barks)[48]
F64	2*α*,3*α*-Dihydroxy-3,24-oxido-friedelan 29-oic acid (Orthosphenic acid)	*Maytenus jelskii* (root barks)[48]<br>*Maytenus macrocarpa* (branches)[204]<br>*Maytenus royleanus* (roots)[209]<br>*Microtropis triflora* (stalks)[270]<br>*Salacia chinensis* (stems)[39]<br>*Salacia nitida* (leaves)[263]
F65	2*α*,3*α*,22*β*-Trihydroxy-21-oxo-29-*nor*-friedelan-24-oic acid	*Salacia crassifolia* (roots)[75]
F66	2*α*-Hydroxy-3-oxo-friedelan-29-oic acid (Wilforic acid C)	*Salacia chinensis* (stems)[39]<br>*Salacia nitida* (lianas)[263]<br>*Tripterygium doianumi* (branches)[233]
F67	2*α*-Hydroxy-3-oxo-friedelan-30-oic acid	*Crossopetalum lobatum* (leaves)[47]
F68	2*β*-Hydroxy-3-oxo-friedelan-30-oic acid	*Salacia longipes* (roots)[221]
F69	24-Formyl-3-hydroxy-2-oxo-friedelan-3-en-29-oic acid (Cangoronine)	*Cassine xylocarpa* (root barks)[196]<br>*Celastrus vulcanicola* (root barks)[196]<br>*Maytenus jelskii* (root barks)[48]<br>*Salacia nitida* (lianas)[263]<br>*Tripterygium doianumi* (branches)[233]<br>*Tripterygium wilfordii* (roots)[272]
F70	3-Hydroxy-2-oxo-friedelan-3-en-29-oic acid (Populninic acid)	*Austroplenckia pulpunea* (stems, roots)[275]<br>*Celastrus vulcanicola* (root barks)[48]<br>*Maytenus jelskii* (root barks)[48]<br>*Salacia amplifolia* (roots)[259]
F71	3-Oxo-friedelan-30-oic acid	*Maytenus jelskii* (root barks)[48]
F72	3-Oxo-friedelan-28-oic acid (Canophylic acid)	*Tripterygium wilfordii* (roots)[92]<br>*Microtropis triflora* (stalks)[270]<br>*Mortonia greggii* (roots, leaves)[271]
F73	3-oxo-friedelan-29-oic acid	*Austroplenckia populnea* (wood barks, roots)[264,276]

Tabela S1: Pentacyclic triterpenoids isolated from Celastraceae species (2001-2021).

	Name	Species (plant part) Reference
	(Populnonic acid)	*Celastrus vulcanicola* (stems, root barks)[48,267]
		Maytenus cuzcoina (root barks)[68]
		Maytenus jelskii (root barks)[48]
		Maytenus senegalensis (root barks)[277]
		Salacia chinensis (stems)[39]
		Salacia chinensis (stems)[39]
		Tripterygium doianumi (branches)[233]
**F74**	3α-Hydroxy-2-oxo-friedelan-29-oic acid	*Maytenus heterophylla* (leaves)[82]
**F75**	3α-Hydroxy-3β,24-oxido-friedelan-29-oic acid (salaspermic acid)	*Maytenus jelskii* (root barks)[48]
		Maytenus royleanus (roots)[209]
		Microtropis triflora (stalks)[270]
		Salacia chinensis (stems)[39]
		Salacia nitida (lianas)[263]
		Salacia staudtiana (root barks)[278]
		Tripterygium wilfordii (roots)[272]
**F76**	3α-Hydroxy-friedelan-28-oic acid	*Maytenus jelskii* (root barks)[48]
**F77**	3β,24-Oxido-2α,24-dihydroxy- friedelan-29-oic acid	*Microtropis triflora* (stalks)[84]
**F78**	3β-Hydroxy-2-oxo-friedelan-29-oic acid (Populnilic acid)	*Austroplenckia populnea* (wood barks)[275]
		Tripterygium doianumi (branches)[233]
**F79**	7β-Hydroxy-3-oxo-friedelan-28-oic acid	*Celastrus vulcanicola* (root barks)[48]
**F80**	Fridelan-3β,29-diol	*Cheiloclinium cognatum* (branches)[69,268]
**F81**	Friedelan-1-en-3,16-dione	*Maytenus robusta* (branches)[45]
**F82**	Friedelan-1,3,21-trione	*Tontelea micrantha* (branches)[52]
**F83**	Friedelan-1,3-dione	*Maytenus salicifolia* (root barks)[105]
		Salacia verrucosa (stems)[49]
**F84**	Friedelan-3,11-dione	*Maytenus gonoclada* (leaves, branches)[36–38]
**F85**	Friedelan-3,12-dione	*Celastrus monospermus* (roots)[35]
		Maytenus gonoclada (leaves, branches, stems, roots)[36,257]
		Maytenus distichophylla (leaves)[72]
**F86**	Friedelan-3,15-dione	*Maytenus robusta* (aerial parts, leaves) [51,264]
**F87**	Friedelan-3,16-dione (Maytensifolin B)	*Maytenus gonoclada* (branches, leaves)[36–38]
		Maytenus robusta (leaves, branches)[46,94,264,279]
		Maytenus salicifolia (roots)[9]
		Salacia elliptica (branches)[43]
**F88**	Friedelan-3,7-dione	*Maytenus distichophylla* (roots)[280]
		Maytenus gonoclada (leaves, stems, roots)[257]
		Maytenus imbricata (roots)[123]
**F89**	Friedelan-3-one (friedelin)	*Austroplenckia populnea* (leaves)[266]
		Cassine xylocarpa (root barks)[196]

Tabela S1: Pentacyclic triterpenoids isolated from Celastraceae species (2001-2021).

	Name	Species (plant part) Reference
		Celastrus hypoleucus (aerial parts)[198]
		Celastrus monospermus (roots)[35]
		Celastrus vulcanicola (stems)[267]
		Cheiloclinium cognatum (leaves, branches)[69,268]
		Crossopetalum lobatum (leaves)[47]
		Elaeodendron buchananii (stem barks)[232]
		Euonymus alatus (root barks)[269]
		Maytenus apurimacensis (roots)[160]
		Maytenus distichophylla (roots, leaves)[72,265,280]
		Maytenus forsskaoliana[281]
		Maytenus gonoclada (branches, leaves, stems, roots) [36–38,257]
		Maytenus guianensis (leaves, stems, root barks)[261,262]
		Maytenus jelskii (root barks)[48]
		Maytenus rígida (stem barks)[282]
		Maytenus robusta (leaves, branches, aerial parts)[46,51,87,94]
		Maytenus salicifolia (fruits, root barks, roots, leaves)[283,105,9, 235]
		Maytenus undata (leaves)[284]
		Microtropis triflora (stalks)[270]
		Salacia chinensis (leaves)[258]
		Salacia crassifolia (leaves)[6,265]
		Salacia elliptica (leaves, branches)[43,44]
		Salacia impressifolia (stems, branches)[285]
		Salacia nitida (lianas)[263]
		Salacia verrucosa (stems)[49]
		Tontelea micrantha (leaves, branches)[52]
		Tripterygium wilfordii (roots)[92,272]
**F90**	Friedelan-3β,11β-diol	*Maytenus robusta* (leaves)[87,94]
**F91**	Friedelan-3β,24-diol	*Maytenus distichophylla* (leaves)[265]
		Salacia crassifolia (leaves)[265]
**F92**	Friedelan-3β,28,29-triol	*Cheiloclinium cognatum* (leaves)[69]
**F93**	Friedelan-3β-ol	*Austroplenckia populnea* (leaves)[266]
		Celastrus vulcanicola (stems, root barks)[48,267]
		Cheiloclinium cognatum (leaves, branches)[69,268]
		Elaeodendron buchananii (stem barks)[232]
		Maytenus distichophylla (leaves)[72,265]
		Maytenus erythroxylon (aerial parts)[273]
		Maytenus gonoclada (branches, leaves)[36–38]
		Maytenus guianensis (stems, root barks)[261,262]
		Maytenus jelskii (root barks)[48]

Tabela S1: Pentacyclic triterpenoids isolated from Celastraceae species (2001-2021).

	Name	Species (plant part) Reference
		Maytenus rígida (stem barks)[282]
		Maytenus robusta (leaves, branches, aerial parts)[46,51,87,94]
		Maytenus salicifolia (leaves)[235]
		Maytenus undata (leaves)[284]
		Salacia crassifolia (leaves)[6,265]
		Salacia elliptica (leaves, branches)[43,44]
		Salacia nitida (leaves)[263]
		Tontelea micrantha (leaves)[52]
**F94**	Friedelan-3,21-dione	*Siphonodon celastrineus* (stems)[54]
		Tontelea micrantha (branches)[52]
**F95**	25-Hydroxy-2,3-*seco*-friedelane-dicarboxylic anhydride (Lobatanhydride)	*Crossopetalum lobatum* (leaves)[47]
**F96**	3-Hydroxy-2-oxo-3(4),20(29)-dien-30-*nor*-friedelan-24-al (Salacenonal)	*Salacia amplifolia* (roots)[259]
**F97**	21-Oxo-2α,3α,22β-trihydroxy-29-*nor*- friedelan-2β,24 olide (Triflora lactone)	*Microtropis triflora* (stalks)[62,270]
**F98**	29-(Formyloxy)-friedelan-3-one (Tripterfrielanon B)	*Tripterygium wilfordii* (roots)[92]
**F99**	2β,22β-Dihydroxy-24,29-*dinor*-friedelan-4-en-3,21-dione (Triptocalline A)	*Salacia chinensis* (stems)[39]
		Tripterygium doianum (branches)[233]
**F100**	3,4-*Seco*-friedelan-3,11β-olide	*Maytenus robusta* (leaves)[94]
**F101**	3,4-*Seco*-28-hydroxy-friedelan--3-oic acid	*Austroplenckia populnea* (leaves)[95]
		Maytenus jelskii (root barks)[48]
**F102**	3,4-*Seco*-friedelan-3-oic acid	*Austroplenckia populnea* (leaves)[95]
		Cheiloclinium cognatum (leaves)[69]
		Maytenus erythroxylon (aerial parts)[273]
		Maytenus imbricata (leaves)[286]
		Maytenus robusta (leaves)[94]
		Salacia crassifolia (leaves)[6]
		Salacia elliptica (leaves)[43]
		Tontelea micrantha (leaves)[52]
**F103**	2,4-*Seco*-4-oxo-23,24-*dinor*-friedelan-1(10),5,7-trien-2,29 dioic dimethyl ester (Dzununcanone)	*Hippocratea excelsa* (root barks)[96]
	**QUINONEMETHIDES**	
**Q1**	11β-Hydroxypristimerin	*Salacia crassifolia* (root barks)[103]
**Q2**	15-Dehydropristimerin	*Maytenus vitis-idaea* (roots)[104]
**Q3**	15α-Hydroxytingenone	*Maytenus vitis-idaea* (roots)[104]
**Q4**	16β-Hydroxypristimerin	*Maytenus salicifolia* (root barks)[105]
**Q5**	20-*epi*-Isoiguesterinol	*Salacia madagascariensis* (roots)[106]

Tabela S1: Pentacyclic triterpenoids isolated from Celastraceae species (2001-2021).

	Name	Species (plant part) Reference
**Q6**	20-Hydroxy-20-*epi*-tingenone	*Cheiloclinium cognatum* (root barks)[115]
		Elaeodendron croceum (leaves)[287]
		Maytenus acanthophylla (roots)[288]
		Peritassa campestris (roots)[252]
		Salacia crassifolia (roots)[243]
**Q7**	20*α*-Hydroxytingenone	*Austroplenckia populnea* (roots)[275]
		Cheiloclinium cognatum (root barks)[109]
**Q8**	20*α*-Hydroxyscutione	*Maytenus vitis-idaea* (roots)[104]
**Q9**	22*β*-Hydroxypristimerin	*Cheiloclinium cognatum* (root barks)[109]
		Maytenus guianensis (steam barks)[261]
**Q10**	22*β*-Hydroxytingenone	*Cheiloclinium cognatum* (root barks)[109,115]
		Elaeodendron croceum (leaves)[287]
		Elaeodendron schlechteranum (root barks)[63]
		Glyptopetalum sclerocarpum (pericarp)[120]
		Maytenus guianensis (stem barks, barks)[261,262,289]
		Salacia chinensis (stems)[39]
		Salacia impressifolia (root barks, trunk and twings)[4,285]
		Peritassa campestris (roots)[252]
		Peritassa laevigata (roots)[290]
**Q11**	30-Hydroxypristimerin	*Salacia impressifolia* (trunk and twings)[285]
**Q12**	Celastrol	*Celastrus monospermus* (roots)[35]
		Celastrus orbiculatus (root barks)[139]
		Cheiloclinium cognatum (root barks)[115]
		Crossopetalum uragoga (stems)[126]
		Maytenus magellanica (root barks)[126]
		Maytenus vitis-idaea (roots)[104]
		Peritassa campestris (roots)[252]
		Tripterygium wilfordii (roots)[272]
**Q13**	Dispermoquinone	*Cheiloclinium hippocratioides* (root barks)[138]
		Crossopetalum uragoga (stems)[126]
		Maytenus apurimacensis (roots)[160]
		Maytenus magellanica (root barks)[126]
		Salacia crassifolia (roots)[243]
**Q14**	Scutione	*Maytenus spinosa* (roots)[104]
		Maytenus vitis-idaea (roots)[104]
**Q15**	Isoiguesterin	*Salacia amplifolia* (roots)[259]
		Salacia madagascariensi (roots)[106]
		Salacia impressifolia (trunk and twings)[285]
**Q16**	Isoiguesterinol (Isoiguesterol)	*Salacia madagascariensis* (roots)[106]
**Q17**	Netzahualcoyene	*Cheiloclinium cognatum* (root barks)[109,115]

Tabela S1: Pentacyclic triterpenoids isolated from Celastraceae species (2001-2021).

	Name	Species (plant part) Reference
		Maytenus ilicifolia (roots)[128]
		Maytenus vitis-idaea (roots)[104]
		Peritassa campestris (roots)[252]
		Salacia amplifolia (roots)[259]
		Salacia crassifolia (root barks)[103]
		Salacia impressifolia (trunk and twings)[285]
		Salacia macrophylla (roots)[291]
**Q18**	Netzahualcoyondiol	*Cheiloclinium cognatum* (root barks)[109,115]
**Q19**	Netzahualcoyonol	*Cheiloclinium cognatum* (root barks)[109]
		Salacia crassifolia (roots)[75,243]
		Salacia macrophylla (roots)[291]
		Salacia petenensis (barks)[64]
**Q20**	Pristimerin	*Celastrus monospermus* (roots)[35]
		Cheiloclinium cognatum (root barks)[109,115]
		Cheiloclinium hippocratioides (root barks)[138]
		Crossopetalum uragoga (stems)[126]
		Gymnosporia heterophylla (aerial parts)[117]
		Hippocratea excelsa (root barks, stem barks)[96,213,292]
		Maytenus acanthophylla (roots)[288]
		Maytenus apurimacensis (roots)[160]
		Maytenus chiapensis (root barks)[293]
		Maytenus chubutensis (root barks)[118]
		Maytenus distichophylla (roots)[280]
		Maytenus ilicifolia (root barks; roots)[128,294]
		Maytenus magellanica (root barks)[118,126]
		Maytenus salicifolia (root barks)[105]
		Maytenus vitis-idaea (roots)[104]
		Mortonia greggii (roots)[295]
		Peritassa campestris (roots)[252]
		Salacia amplifolia (roots)[259]
		Salacia crassifolia (root barks; roots)[75,103,243,296]
		Salacia impressifolia (root barks, trunk and twings)[285]
		Salacia leptoclada (root barks)[297]
		Salacia macrophylla (roots)[291]
		Salacia nitida (lianas)[263]
		Siphonodon celastrineus (root barks)[197]
**Q21**	Tingenol	*Cheiloclinium cognatum* (root barks)[115]
		Peritassa campestris (roots)[298]
**Q22**	Tingenone (Maytein)	*Cheiloclinium cognatum* (root barks)[109,115]
		Crossopetalum uragoga (stems)[126]

Tabela S1: Pentacyclic triterpenoids isolated from Celastraceae species (2001-2021).

	Name	Species (plant part) Reference
		Elaeodendron croceum (leaves)[287]
		Hippocratea excelsa (root barks)[96,213]
		Maytenus acanthophylla (roots)[288]
		Maytenus apurimacensis (roots)[160]
		Maytenus chiapensis (root barks)[293]
		Maytenus gonoclada (root barks, roots)[257,299]
		Maytenus guianensis (stem barks, bark)[261,262,289,300]
		Maytenus ilicifolia (roots, root barks)[128,294]
		Maytenus imbricata (roots)[123]
		Maytenus magellanica (root barks)[126]
		Maytenus salicifolia (root barks)[105]
		Maytenus spinosa (roots)[104]
		Maytenus vitis-idaea (roots)[104]
		Peritassa campestris (roots)[252]
		Salacia chinensis (stems)[39]
		Salacia crassifolia (roots)[75]
		Salacia impressifolia (trunk and twings, root barks)[4,285]
		Salacia nitida (lianas)[263]
		Salacia petenensis (barks)[64]
	**AROMATICS**	
**A1**	3-Methoxy-4-deoxy-7,8-dihydrozeylasterone	*Gymnosporia heterophylla* (aerial parts)[117]
**A2**	22*β*-Hydroxyisotingenone III	*Glyptopetalum sclerocarpum* (pericarp)[120]
**A3**	3-Methoxy-6-oxopristimerol	*Maytenus chubutensis* (root barks)[118]
**A4**	3-Methoxy-6-oxotingenol	*Maytenus canariensis* (root barks)[301]
**A5**	6-Deoxoblepharodol	*Maytenus blepharodes* (root barks)[119,301]
**A6**	6-Oxo-20-hydroxy-20-*epi*-tingenol	*Glyptopetalum sclerocarpu* (pericarp)[120]
**A7**	6-Oxoisoiguesterin	*Salacia madagascariensis* (roots)[106]
**A8**	6-Oxoiguesterol	*Maytenus canariensis* (root barks)[301]
**A9**	6-Oxopristimerol	*Cheiloclinium hippocratioides* (root barks)[138]
		Maytenus apurimacensis (roots)[160]
		Maytenus blepharodes (root barks)[119]
		Maytenus canariensis (root barks)[301]
		Maytenus chubutensis (root barks)[118]
		Maytenus ilicifolia (roots)[128]
		Maytenus magellanica (root barks)[118]
		Salacia crassifolia (root barks, roots)[103,243]
**A10**	6-Oxotingenol	*Maytenus canariensis* (root barks)[301]
		Maytenus imbricata (roots)[123]
**A11**	7*α*-Hydroxyblepharodol	*Cheiloclinium hippocratioides* (root barks)[138]
		Maytenus blepharodes (root barks)[301]

Tabela S1: Pentacyclic triterpenoids isolated from Celastraceae species (2001-2021).

	Name	Species (plant part) Reference
**A12**	7-Hydroxy-6-oxopristimerol	*Maytenus blepharodes* (root barks)[119,301]
		Salacia amplifolia (roots)[259]
		Salacia macrophylla (roots)[291]
**A13**	7-Oxoblepharodol	*Cheiloclinium hippocratioides* (root barks)[138]
		Maytenus blepharodes (root barks)[119,301]
**A14**	Blepharodol	*Cheiloclinium hippocratioides* (root barks)[138]
		Maytenus apurimacensis (roots)[160]
		Maytenus blepharodes (root barks)[301]
		Maytenus magellanica (root barks)[126]
**A15**	Blepharodin	*Maytenus magellanica* (root barks)[126]
**A16**	Blepharotriol	*Maytenus blepharodes* (root barks)[119,301]
**A17**	Canarol (7,8-dihydro-6-oxoiguesterol)	*Maytenus canariensis* (root barks)[301]
**A18**	Cognatine	*Cheiloclinium cognatum* (root barks)[109]
**A19**	Demethylzeylasteral	*Maytenus blepharodes* (root barks, roots)[119,301,302]
		Tripterygium wilfordii (roots)[303]
**A20**	Demethylzeylasterone	*Maytenus blepharodes* (root barks)[119,301]
**A21**	Isoblepharodol	*Cheiloclinium hippocratioides* (root barks)[138]
		Maytenus blepharodes (root barks)[119]
**A22**	Milicifoline A	*Maytenus ilicifolia* (roots)[128]
**A23**	Regeol A	*Salacia amplifolia* (roots)[259]
		Salacia chinensis (stems)[39]
		Salacia impressifolia (trunk and twings)[286]
**A24**	Salaquinone B	*Salacia chinensis* (trunk)[39]
**A25**	Triptotin F	*Tripterygium wilfordii* (roots)[130]
**A26**	Triptotin G	*Tripterygium wilfordii* (roots)[130]
**A27**	Wilforol B	*Celastrus orbiculatus* (root barks)[139]
**A28**	Zeylasteral	*Maytenus apurimacensis* (roots)[160]
		Maytenus blepharodes (root barks, roots)[301,302]
**A29**	Zeylasterone	*Maytenus apurimacensis* (roots)[160]
		Maytenus blepharodes (root barks)[301]
	**DIMERS**	
**D1**	6',7'-Dihydroscutionin αB	*Maytenus blepharodes* (root barks)[133]
		Maytenus magellanica (roots)[133]
**D2**	6',7'-Dihydroisocangorosin A	*Maytenus apurimacensis* (roots)[160]
**D3**	6'β-Methoxy-6',7'-dihydroxoscutionin αB	*Maytenus blepharodes* (root barks)[133]
		Maytenus magellanica (roots)[133]
**D4**	7,8-Dihydroscutidin Bα	*Maytenus ilicifolia* (roots)[128]
**D5**	7,8-Dihydroscutionin Aα	*Cheiloclinium hippocratioides* (root barks)[138]
**D6**	7,8-Dihydroisoxuxuarine Eα	*Maytenus ilicifolia* (roots)[128]
		Maytenus spinosa (roots)[104]

Tabela S1: Pentacyclic triterpenoids isolated from Celastraceae species (2001-2021).

	Name	Species (plant part) Reference
**D7**	7,8-Dihydroisoxuxuarine Fα	*Maytenus chuchuhuasca* (root barks)[136]
**D8**	7,8-Dihydroisoxuxuarine Gα	*Maytenus chuchuhuasca* (root barks)[136]
**D9**	7,8-Dihydroisoxuxuarine Hα	*Maytenus chuchuhuasca* (root barks)[137]
**D10**	7,8-Dihydroisoxuxuarine Iα	*Maytenus chuchuhuasca* (root barks)[137]
**D11**	7',8'-Dihydroxuxuarine Aα	*Maytenus chuchuhuasca* (root barks)[136]
**D12**	7',8'-Dihydroxuxuarine Dβ	*Maytenus chuchuhuasca* (root barks)[136]
**D13**	7α-Hydroisoxuxuarine Eα	*Maytenus chuchuhuasca* (root barks)[136]
**D14**	Cangorosin A	*Cheiloclinium hippocratioides* (root barks)[138]
**D15**	Caryopristimerin	*Salacia crassifolia* (roots)[75,243]
**D16**	Cheilocline A	*Cheiloclinium hippocratioides* (root barks)[138]
**D17**	Cheilocline B	*Cheiloclinium hippocratioides* (root barks)[138]
**D18**	Cheilocline C	*Cheiloclinium hippocratioides* (root barks)[138]
**D19**	Cheilocline D	*Cheiloclinium hippocratioides* (root barks)[138]<br>*Maytenus ilicifolia* (roots)[128]
**D20**	Cheilocline E	*Cheiloclinium hippocratioides* (root barks)[138]
**D21**	Cheilocline F	*Cheiloclinium hippocratioides* (root barks)[138]
**D22**	Cheilocline G	*Cheiloclinium hippocratioides* (root barks)[138]
**D23**	Cheilocline H	*Cheiloclinium hippocratioides* (root barks)[138]
**D24**	Cheilocline I	*Cheiloclinium hippocratioides* (root barks)[138]
**D25**	Celastroline Aα	*Celastrus orbiculatus* (root barks)[139]
**D26**	Celastroline Aβ	*Celastrus orbiculatus* (root barks)[139]
**D27**	Celastroline Bα	*Celastrus orbiculatus* (root barks)[139]
**D28**	Celastroline Bβ	*Celastrus orbiculatus* (root barks)[139]
**D29**	Scutionin Aα	*Cheiloclinium hippocratioides* (root barks)[138]
**D30**	Scutionin Bα	*Maytenus blepharodes* (root barks)[133]<br>*Maytenus magellanica* (roots)[133]
**D31**	Isocangorosin A	*Cheiloclinium hippocratioides* (root barks)[138]
**D32**	Isocelastroline Aα	*Celastrus orbiculatus* (root barks)[139]
**D33**	Isoxuxuarine Eβ	*Maytenus chuchuhuasca* (root barks)[136]
**D34**	Isoxuxuarine Fα (Cangorosin B)	*Maytenus chuchuhuasca* (root barks)[136]
**D35**	Isoxuxuarine Gβ	*Maytenus chuchuhuasca* (root barks)[136]
**D36**	Isoxuxuarine Hα	*Maytenus chuchuhuasca* (root barks)[137]
**D37**	Milicifoline B	*Maytenus ilicifolia* (roots)[128]
**D38**	Milicifoline C	*Maytenus ilicifolia* (roots)[128]
**D39**	Milicifoline D	*Maytenus ilicifolia* (roots)[128]
**D40**	Xuxuarine Eβ	*Hippocratea excelsa* (root barks)[96]
**D41**	Xuxuarine Fα	*Maytenus chuchuhuasca* (root barks)[136]
**D42**	Xuxuarine Hα	*Maytenus chuchuhuasca* (root barks)[137]
**D43**	Xuxuarine Hβ	*Maytenus chuchuhuasca* (root barks)[137]
**D44**	Xuxuarine Iα	*Maytenus chuchuhuasca* (root barks)[137]

Tabela S1: Pentacyclic triterpenoids isolated from Celastraceae species (2001-2021).

	Name	Species (plant part) Reference
**D45**	Xuxuarine I*β*	*Maytenus chuchuhuasca* (root barks)[137]
**D46**	Xuxuarine J*α*	*Maytenus chuchuhuasca* (root barks)[137]
**D47**	Xuxuarine K*α*	*Maytenus chuchuhuasca* (root barks)[137]
**D48**	Xuxuasine A	*Maytenus chuchuhuasca* (root barks)[141]
**D49**	Xuxuasine B	*Maytenus chuchuhuasca* (root barks)[141]
**D50**	Truncatin	*Maytenus truncata* (roots)[142]
**LUPANES**		
**L1**	(20*R*)-3*β*-Hydroxy-lupan-29-al (29-oxo-lupanol)	*Euonymus alatus* (branches)[145]
**L2**	(20*S*)-3*β*-Hydroxy-lupan-30-al (30-oxo-lupanol)	*Euonymus alatus* (branches)[145]
**L3**	11*α*,28-Dihydroxy-lup-20(29)-en-3-one	*Maytenus cuzcoina* (root barks)[146]
**L4**	11*α*-Hydroxy-lup-1,20(29)-dien-3-one<br>(11*α*-Hydroxy-glochidone)	*Maytenus cuzcoina* (root barks)[146]<br>*Maytenus salicifolia* (root barks)[105]
**L5**	11*α*-Hydroxy-lup-20(29)-en-3-one<br>(Rigidenol)	*Cassine xylocarpa* (caules)[146]<br>*Maytenus acanthophylla* (stem barks)[288]<br>*Maytenus cuzcoina* (root barks)[151]<br>*Maytenus distichophylla* (roots)[280]<br>*Maytenus imbricata* (branches, roots)[123,148]<br>*Maytenus salicifolia* (root barks)[105]
**L6**	16*β*-Hydroxy-lup-20(29)-en-3-one	*Maytenus elaeodendroides* (branches)[149]<br>*Maytenus chiapensis* (leaves)[151]<br>*Salacia chinensis* (leaves)[258]
**L7**	17*β*-Hydroxy-28-*nor*-lup-20(29)-en-3-one	*Euonymus alatus* (branches)[145]
**L8**	1*α*-Hydroxy-lup-20(29)-en-3-one<br>(Glochidonol)	*Maytenus cuzcoina* (root barks)[151]<br>*Maytenus chiapensis* (leaves)[151]<br>*Maytenus salicifolia* (root barks)[105]
**L9**	21*β*-Hydroxy-lup-20(29)-en-3-one<br>(Salacianol)	*Salacia chinensis* (stems)[14]
**L10**	24,28-Dihydroxy-lup-20(29)-en-3-one	*Maytenus chiapensis* (leaves)[151]
**L11**	28,30-Dihydroxy-lup-20(29)-en-3-one	*Euonymus alatus* (branches)[157]<br>*Maytenus chiapensis* (leaves)[151]<br>*Microtropis fokienensis* (stems)[153]
**L12**	28-Hydroxy-3-oxo-lup-20(29)-en-30-al	*Microtropis fokienensis* (stems)[153]
**L13**	28-Hydroxy-lup-1,20(29)-dien-3-one (28-Hydroxy-glochidone)	*Maytenus cuzcoina* (root barks)[151]
**L14**	28-Hydroxy-lup-20(29)-3-one<br>(Betulone)	*Euonymus alatus* (branches)[157]<br>*Maytenus chiapensis* (leaves)[151]<br>*Maytenus cuzcoina* (root barks)[146,151]<br>*Cassine transvaalensis*[304]<br>*Maytenus elaeodendroides* (branches)[149]

Tabela S1: Pentacyclic triterpenoids isolated from Celastraceae species (2001-2021).

	Name	Species (plant part) Reference
		Salacia chinensis (leaves)[258]
**L15**	29-*nor*-Lupan-3,20-dione	*Maytenus erythroxylon* (aerial parts)[273]<br>*Salacia chinensis* (leaves, stems)[14,258]
**L16**	30-Hydroxy-lup-20(29)-en-3-one	*Cassine xylocarpa* (stems)[146]<br>*Maytenus distichophylla* (roots)[280]<br>*Maytenus erythroxylon* (aerial parts)[273]<br>*Maytenus imbricata* (branches)[148]<br>*Maytenus salicifolia* (root barks)[105]<br>*Microtropis fokienensis* (stems)[153]<br>*Mortonia greggii* (leaves)[271]<br>*Salacia chinensis* (leaves, stems)[14,258]
**L17**	3α-Hydroxy-lup-20(29)-en-28,19-olide (3-*epi*-Thurberogenin)	*Microtropis fokienensis* (stems)[153]
**L18**	3-Oxo-11α-hydroxy-lup-20(29)-en-28-al	*Maytenus cuzcoina* (root barks)[151]
**L19**	3-Oxo-lup-1,20(29)-dien-28-al	*Maytenus cuzcoina* (root barks)[151]
**L20**	3-Oxo-lup-20(29)-en-30-al	*Cassine xylocarpa* (stems)[146]<br>*Maytenus imbricata* (branches)[148]<br>*Maytenus spinosa* (roots)[104]
**L21**	3α,28-Dihydroxy-lup-20(29)-en-2-one	*Salacia hainanensis* (roots)[73]
**L22**	3α-Acetyloxy-28-hydroxy-29-norlup-18(19)-en-20-one	*Euonymus carnosus* (stems)[154]
**L23**	3α-Acetyloxy-lup-20(29)-en-30-al	*Euonymus carnosus* (stems)[154]
**L24**	3α-Hydroxy-lup-20(29)-en-28-al	*Maytenus cuzcoina* (root barks)[151]<br>*Microtropis fokienensis* (stems)[153]
**L25**	3α-Hydroxy-lup-20(29)-en-2-one	*Salacia hainanensis* (roots)[73]
**L26**	3β-(*E*)-*p*-Coumaroyloxy-lup-20(29)-en-28-ol [3β-(*E*)-*p*-coumaroyloxybetulin]	*Maytenus macrocarpa* (branches)[204]
**L27**	3β-(*Z*)-*p*-Coumaroyloxy-lup-20(29)-en-28-ol [3β-(*Z*)-*p*-coumaroyloxybetulin]	*Maytenus macrocarpa* (branches)[204]
**L28**	3β,17β-Dihydroxy-28,29-bisnorlupan-20-one	*Euonymus alatus* (branches)[145]
**L29**	3β,25-Oxido-25-methoxy-lup-20(29)-ene	*Siphonodon celastrineus* (stems)[54]
**L30**	3β,28-Dihydroxy-29-*nor*-lupan-20-one (Messagenin)	*Euonymus alatus* (branches)[157]<br>*Maytenus chiapensis* (leaves)[151]
**L31**	3β,28-Dihydroxy-lup-20(29)-en-2-one	*Salaca impressifolia* (stems, branches)[285]
**L32**	3β,30-Dihydroxy-lup-20(29)-en-28-al	*Euonymus alatus* (branches)[145]
**L33**	3β,7β-Dihydroxy-lup-20(29)-en-28-oic acid methyl ester (7β-Hydroxymethyl betulinate)	*Microtropis fokienensis* (stems)[153]
**L34**	3β-Acetyloxy-lup-20(29)-en-1β-ol	*Gymnosporia heterophylla* (aerial parts)[117]
**L35**	3β-caffeoyloxy-lup-20(29)-en-28-ol (3β-caffeoyloxybetulin)	*Maytenus chiapensis* (leaves)[151]<br>*Maytenus cuzcoina* (root barks)[146,151]

Tabela S1: Pentacyclic triterpenoids isolated from Celastraceae species (2001-2021).

	Name	Species (plant part) Reference
		Maytenus macrocarpa (branches)[204]
**L36**	3*β*-caffeoyloxy-1*β*-hydroxy-lup-20(29)-ene	*Maytenus apurimacensis* (roots)[160]
**L37**	3*β*-caffeoyloxy-lup-20(29)-ene	*Maytenus chiapensis* (leaves)[151]<br>*Maytenus cuzcoina* (root barks)[146,151]
**L38**	3*β*-lup-20(29)-en-3-yl stearate	*Maytenus salicifolia* (leaves)[161]
**L39**	3*β*-Hydroxy-20-oxo-29-*nor*-lupan-28-al	*Euonymus alatus* (branches)[145]
**L40**	3*β*-Hydroxy-29-*nor*-lupan-20-one	*Euonymus alatus* (branches)[145]<br>*Salacia chinensis* (leaves, stems)[14,258]
**L41**	3*β*-Hydroxy-lup-20(29)-en-2-one	*Salacia impressifolia* (stems, branches)[285]
**L42**	3*β*-Hydroxy-lup-20(29)-en-28-al	*Maytenus cuzcoina* (root barks)[151]<br>*Maytenus chiapensis* (leaves)[151]
**L43**	3*β*-Hydroxy-lup-20(29)-en-30-al (30-oxo-lupeol)	*Euonymus alatus* (branches)[145]
**L44**	6*β*,20-Dihydroxy-lupan-3-one	*Cassine xylocarpa* (branches)[146]
**L45**	6*β*,28-Dihydroxy-lup-20(29)-en-3-one	*Maytenus chiapensis* (leaves)[151]
**L46**	6*β*,30-Dihydroxy-lup-20(29)-en-3-one	*Cassine xylocarpa* (stems)[146]
**L47**	6*β*-Hydroxy-3-oxo-lup-20(29)-en-30-al	*Cassine xylocarpa* (stems)[146]
**L48**	3*β*-Acetyloxy-lup-20(29)-ene	*Maytenus acanthophylla* (leaves)[165]
**L49**	2,3-*Seco*-lup-20(29)-en-2,3-dioic acid	*Salacia hainanensis* (roots)[73]
**L50**	30-Hydroxy-2,3-*seco*-lup-20(29)-en-2,3-dioc acid	*Microtropis fokienensis* (stems)[153]
**L51**	3-Oxo-23-hydroxy-lup-20(29)-en-28-oic acid	*Celastrus orbiculatus* (stems)[166]
**L52**	3-Oxo-6*β*-hydroxy-lup-20(29)-en-28-oic acid	*Maytenus chiapensis* (leaves)[151]
**L53**	3-Oxo-lup-20(29)-en-28-oic acid<br>(Betulonic acid)	*Maytenus elaeodendroides* (barks)[149]<br>*Salacia chinensis* (stems)[14]
**L54**	3-Oxo-lup-20(29)-en-30-oic acid	*Cassine xylocarpa* (stems)[146]
**L55**	3,4-*Seco*-28-methyloxycarbonyl-lup-4(23):20(29)-diene-3-oic acid	*Maytenus magellanica* (root barks)[118]
**L56**	3*α*-Hydroxy-7*β*-senecioyloxy-lup-20(29)-en-28-oic acid (7*β*-senecioyl-3-*epi*-betulinic acid)	*Microtropis fokienensis* (stems)[153]
**L57**	3*α*-Hydroxy-lup-20(29)-en-30-oic acid	*Euonymus carnosus* (stems)[154]
**L58**	3*α*-Hydroxy-lup-20(29)-en-28-oic acid<br>(Betulínic acid 3-*epi*)	*Maytenus cuzcoina* (root barks)[151]<br>*Microtropis fokienensis* (stems)[153]
**L59**	3*β*-Hydroxy-lup-20(29)-en-28-oic acid<br>(Betulinic acid)	*Euonymus alatus* (branches, stems)[169,305]<br>*Euonymus grandiflorus* (leaves, branches)[306]<br>*Maytenus cuzcoina* (root barks)[151]<br>*Microtropis fokienensis* (stems)[153]<br>*Salacia chinensis* (leaves)[258]
**L60**	3*β*-Methoxy-lup-20(29)-en-28-oic acid	*Microtropis fokienensis* (stems)[153]
**L61**	Lup-1,20(29)-dien-3-one	*Cassine xylocarpa* (stems)[146]

Tabela S1: Pentacyclic triterpenoids isolated from Celastraceae species (2001-2021).

	Name	Species (plant part) Reference
	(Glochidone)	*Maytenus chiapensis* (leaves)[151]
		Maytenus cuzcoina (root barks)[146,151]
		Maytenus salicifolia (root barks)[105]
**L62**	Lup-20(29)-en-3β,30-diol	*Cassine xylocarpa* (branches)[146]
	(30-Hydroxylupeol)	*Euonymus alatus* (branches)[145]
		Maytenus imbricata (stems, branches)[148,307]
		Maytenus rigida (branche barks)[282]
		Maytenus salicifolia (roots)[9]
		Microtropis fokienensis (stems)[153]
		Microtropis triflora (stalks)[84,270]
		Mortonia greggii (roots, leaves)[271]
		Salacia chinensis (leaves, stems)[14,258]
**L63**	Lup-20(29)-en-1β,3α,28-triol	*Maytenus cuzcoina* (root barks)[146]
**L64**	Lup-20(29)-en-1β,3α-diol	*Maytenus apurimacensis* (roots)[160]
	(glochidiol)	*Maytenus chiapensis* (leaves) [151]
		Maytenus cuzcoina (root barks)[146,151]
**L65**	Lup-20(29)-en-1β,3β-diol	*Cassine xylocarpa* (branches)[146]
	(3-*epi*-glochidiol)	*Gymnosporia heterophylla* (aerial parts)[117]
		Maytenus apurimacensis (roots)[160]
		Maytenus chiapensis (leaves)[151]
		Maytenus cuzcoina (root barks)[151]
		Maytenus heterophylla (leaves)[82]
		Maytenus salicifolia (root barks)[105]
		Microtropis triflora (stalks)[84]
**L66**	Lup-20(29)-en-2β,3β-diol	*Salacia impressifolia* (stems, branches)[285]
**L67**	Lup-20(29)-en-3,21-dione	*Salacia hainanensis* (roots)[73]
**L68**	Lup-20(29)-en-3-one (Lupenone)	*Euonymus alatus* (stems, branches)[145,157,305]
		Glyptopetalum calocarpum (leaves)[308]
		Maytenus chiapensis (leaves)[151]
		Maytenus cuzcoina (root barks)[151]
		Maytenus salicifolia (root barks)[105]
		Microtropis triflora (stems)[270]
**L69**	Lup-20(29)-en-3α,11α,28-triol	*Maytenus cuzcoina* (root barks)[151]
**L70**	Lup-20(29)-en-3α,11α-diol (3-*epi*-nepeticin)	*Maytenus cuzcoina* (root barks)[146]
**L71**	Lup-20(29)-en-3α,28-diol	*Maytenus cuzcoina* (root barks)[146,151]
	(3-*epi*-betulin)	*Maytenus chiapensis* (leaves)[151]
		Microtropis fokienensis (stems)[153]
**L72**	Lup-20(29)-en-3α-ol (3-*epi*-lupeol)	*Euonymus alatus* (stems)[305]
**L73**	Lup-20(29)-en-3β,11α-diol	*Maytenus imbricata* (roots)[123]
	(Nepeticin)	*Maytenus cuzcoina* (root barks)[146,151]

Tabela S1: Pentacyclic triterpenoids isolated from Celastraceae species (2001-2021).

	Name	Species (plant part) Reference
		Maytenus macrocarpa (branches)[204]
		Maytenus salicifolia (root barks)[105]
**L74**	Lup-20(29)-en-3β,15α-diol	*Salacia chinensis* (leaves)[258]
**L75**	Lup-20(29)-en-3β,16β-diol	*Maytenus elaeodendroides* (branches)[149]
**L76**	Lupan-3β,20-diol	*Salacia chinensis* (leaves)[258]
**L77**	Lup-20(29)-en-3β,25-diol (25-Hydroxylupeol)	*Maytenus cuzcoina* (roots)[146]
**L78**	Lup-20(29)-en-3β,28,30-triol	*Euonymus alatus* (branches)[145,157]
**L79**	Lup-20(29)-en-3β,28-diol	*Cassine transvaalensis*[304]
	(Betulin)	*Euonymus alatus* (branches)[145,157,169]
		Gymnosporia montana (leaves)[309]
		Maytenus boaria (leaves)[310]
		Maytenus chiapensis (leaves)[151]
		Maytenus cuzcoina (root barks)[146,151]
		Maytenus rígida (stem barks)[282]
		Maytenus salicifolia (roots)[9]
		Salacia chinensis (leaves)[258]
		Salacia staudtiana (root barks)[278]
**L80**	Lup-20(29)-en-3β,30-diol	*Cassine xylocarpa* (root barks)[146]
		Maytenus imbricata (branches, roots)[148,307]
		Microtropis fokienensis (leaves)[153,189]
		Microtropis triflora (stems)[270]
**L81**	Lup-20(29)-en-3β,6β-diol	*Maytenus chiapensis* (leaves)[151]
**L82**	Lup-20(29)-en-3β-ol (Lupeol)	*Euonymus alatus* (branches, stems)[145,157,169,305]
		Glyptopetalum calocarpum (leaves)[308]
		Gymnosporia heterophylla (aerial parts)[117]
		Maytenus acanthophylla (leaves)[165]
		Maytenus apurimacensis (roots)[160]
		Maytenus boaria (leaves)[310]
		Maytenus chiapensis (leaves)[151]
		Maytenus cuzcoina (root barks)[146,151]
		Maytenus gonoclada (branches)[37]
		Maytenus phyllanthoides (leaves)[311]
		Maytenus salicifolia (fruits, root barks, roots, leaves)[9,105,235,283]
		Microtropis fokienensis (stems)[153]
		Microtropis triflora (caules, stalks)[84,270]
		Mortonia greggii (roots, leaves)[271]
		Salacia impressifolia (stems, branches)[285]
**L83**	Lup-20(29)-en-3β,23,30-triol	*Euonymus alatus* (branches)[145]
**L84**	Lupan-3β,6β,20-triol	*Cassine* xylocarpa (stems)[146]

Tabela S1: Pentacyclic triterpenoids isolated from Celastraceae species (2001-2021).

	Name	Species (plant part) Reference
**L85**	Lup-20(29)-en-3β,6β,28-triol	*Maytenus chiapensis* (leaves)[151]
**L86**	3-Oxo-lup-20(29)-en-30,21-olide (Ochraceolideo A)	*Cassine xylocarpa* (branches)[146]<br>*Elaeodendron trichotomum* (stem barks)[312]
**L87**	3β-Hydroxy-lup-20(29)-en-28,19-olide (Thurberogenina)	*Microtropis fokienensis* (stems)[153]
**L88**	3-Oxo-lup-20(29)-en-28,19-olide (Thurberogenona)	*Microtropis fokienensis* (stems)[153]
**L89**	Lup-20(29)-eno (α-Lupene)	*Glyptopetalum calocarpum* (leaves)[308]
	**OLEANANES**	
**O1**	11α,12α-Epoxy-3-oxo-oleanan-13β,28-olideo (Liquidambaric lactone)	*Euonymus grandiflorus* (leaves, branches)[306]
**O2**	11α,12α-Epoxy-3α,30-dihydroxy-oleanan-13β,28-olide	*Maytenus diversifolia* (aereal parts)[185]
**O3**	11α-Hydroxy-olean-12-en-3-one	*Maytenus cuzcoina* (root barks)[68]
**O4**	11α-Methoxy-olean-12-en-3-one	*Euonymus alatus* (branches)[157]<br>*Maytenus spinosa* (roots)[104]
**O5**	11α-Methoxy-olean-12-en-3α,21β-diol	*Hippocratea excelsa* (stem barks)[187]
**O6**	11α-Methoxy-olean-12-en-3β-ol	*Siphonodon celastrineus* (stems)[54]
**O7**	11β,21β-Dihydroxy-olean-12-en-3-one	*Hippocratea excelsa* (stem barks)[187]
**O8**	11β-Hydroxy-olean-12-en-3-one	*Hippocratea excelsa* (stem barks)[187]
**O9**	13β,28-Oxido-30-hydroxy-olean-11-en-3-one (Microfokienoxane D)	*Microtropis fokienensis* (leaves)[189]
**O10**	1α,3β-Dihydroxy-olean-12-en-11-one	*Microtropis fokienensis* (stems)[190]
**O11**	21α-Hydroxy-olean-18-en-3-one	*Cassine xylocarpa* (stems)[191]
**O12**	21β,28-Dihydroxy-29-*nor*-olean -12,20(30)-dien-3-one	*Tripterygium wilfordii* (roots)[61]
**O13**	21β-Hydroxy-olean-12-en-3-one	*Hippocratea excelsa* (root barks, stem barks)[96,187]<br>*Tripterygium hypoglaucum* (aereal parts)[313]
**O14**	28-Hydroxy-olean-12-en-3,11-dione (Krukovines A)	*Euonymus carnosus* (stems)[154]
**O15**	22α-Hydroxy-3β-tetradecanoyloxy-olean-12-en-29-oic acid methyl ester	*Maytenus cuzcoina* (root barks)[68]
**O16**	29-Hydroxy-olean-18-en-3-one	*Cassine xylocarpa* (stems)[191]
**O17**	2α,3β,11α,13β-Tetrahydroxy-olean-12-one	*Siphonodon celastrineus* (stems)[193]
**O18**	2α,3β,19α,24-Tetrahydroxy-olean-12-en-28-oic acid 28-O-β-D-glucopyranosyl-(1→2)-β-D-glucopyranoside	*Salacia cochinchinensis* (leaves, branches)[194]

Tabela S1: Pentacyclic triterpenoids isolated from Celastraceae species (2001-2021).

	Name	Species (plant part) Reference
**O19**	2α,3β,24-Trihydroxy-olean-12-en-28-oic acid 28-O-β-D-glucopyranosyl-(1→2)-β-D-glucopyranoside	*Salacia cochinchinensis* (leaves, branches)[194]
**O20**	2α,3β-Dihydroxy-olean-12-en-22α,29-olide	*Tripterygium wilfordii* (roots)[61]
**O21**	3,4-*Seco*-1-hidroxi-21-oxo-olean-12-en-3,11-olide	*Gymnosporia heterophylla* (aereal parts)[117]
**O22**	30-Hydroxy-olean-12-en-3,11-dione	*Microtropis fokienensis* (stems)[190]
**O23**	3-Oxo-olean-12-en-22α,29-olide (Wilforlide B)	*Celastrus orbiculatus* (stems)[314]<br>*Euonymus grandiflorus* (leaves, branches)[306]<br>*Microtropis triflora* (stalks)[84]<br>*Salacia amplifolia* (roots)[259]<br>*Tripterygium hypoglaucum* (aereal parts)[313]<br>*Tripterygium wilfordii* (roots)[92]
**O24**	3α,21α-Dihydroxy-3β,25-oxido-olean-12-ene (Xyloketal)	*Cassine xylocarpa* (root barks)[196]<br>*Celastrus vulcanicola* (root barks)[196]
**O25**	3α,28-Dihydroxy-olean-12-en-11-one	*Euonymus carnosus* (stems)[154]
**O26**	3α-Acetyloxy-28-hydroxy-olean-12-en-11-one	*Euonymus carnosus* (stems)[154]
**O27**	3β,12-Dihydroxy-olean-12-en-11-one	*Siphonodon celastrineus* (stems)[54]
**O28**	3β,28-Dihydroxy-olean-18-en-1-one	*Microtropis fokienensis* (stems)[190]
**O29**	3β-Acetyloxy-11α-benzoyloxy-13β-hydroxy-oleanan-12-one	*Siphonodon celastrineus* (root barks)[197]
**O30**	3β-Caffeoyloxy-olean-12-ene	*Celastrus hypoleucus* (aereal parts)[198]<br>*Crossopetalum uragoga* (stems)[126]<br>*Maytenus magellanica* (root barks)[126]
**O31**	3β-Caffeoyloxy-olean-9(11),12-diene	*Celastrus hypoleucus* (aereal parts)[198]<br>*Crossopetalum uragoga* (stems)[126]<br>*Maytenus magellanica* (root barks)[126]
**O32**	3β-Olean-18-en-3-yl stearate	*Maytenus salicifolia* (leaves)[161]
**O33**	3β-Olean-12-en-3-yl stearate	*Austroplenckia populnea* (leaves)[199]<br>*Salacia crassifolia* (leaves)[43]<br>*Salacia elliptica* (leaves)[265]
**O34**	3β-Hydroxy-2-oxo-olean-12-en-22α,29-olide	*Celastrus orbiculatus* (stems)[166]
**O35**	13β,28-Oxido-olean-11(12)-en-3β-ol	*Microtropis fokienensis* (leaves, stems)[153,189]
**O36**	3β-Hydroxy-olean-12-en-11-one	*Maytenus cuzcoina* (root barks)[68]
**O37**	3β-Hydroxy-olean-12-en-22α,29-olide (Abruslactona A; Regelide; Wilforlide A)	*Austroplenckia populnea* (roots)[276]<br>*Celastrus orbiculatus* (stems)[314]<br>*Euonymus alatus* (root barks)[269]

Tabela S1: Pentacyclic triterpenoids isolated from Celastraceae species (2001-2021).

	Name	Species (plant part) Reference
		Euonymus grandiflorus (leaves, branches)[306]<br>*Maytenus salicifolia* (root barks)[105]<br>*Microtropis triflora* (stalks)[84]<br>*Salacia amplifolia* (roots)[259]<br>*Salacia crassifolia* (roots)[243]<br>*Salacia nitida* (lianas)[263]<br>*Tripterygium hypoglaucum* (aereal parts)[313]<br>*Tripterygium wilfordii* (roots)[92,272]
**O38**	3*β*-Hydroxy-olean-12-ene (*β*-amirin)	*Celastrus hypoleucus* (aereal parts)[198]<br>*Cheiloclinium cognatum* (branches)[268]<br>*Crossopetalum uragoga* (stems)[126]<br>*Euonymus alatus* (root barks)[269]<br>*Euonymus carnosus* (stems)[154]<br>*Euonymus grandiflorus* (leaves, branches)[306]<br>*Glyptopetalum calocarpum* (leaves)[308]<br>*Hippocratea excelsa* (stem barks)[187]<br>*Maytenus boaria* (leaves)[310]<br>*Maytenus distichophylla* (leaves)[265]<br>*Maytenus forsskaoliana*[281]<br>*Maytenus magellanica* (root barks)[126]<br>*Maytenus phyllanthoides* (leaves)[311]<br>*Maytenus salicifolia* (fruits, leaves)[235,283]<br>*Microtropis fokienensis* (stems)[153]<br>*Microtropis triflora* (caules, stalks)[84,270]<br>*Salacia amplifolia* (roots)[259]<br>*Salacia chinensis* (stems)[39]<br>*Salacia crassifolia* (leaves)[265]<br>*Salacia hainanensis* (roots)[73]<br>*Salacia impressifolia* (stems, branches)[285]
**O39**	3*β*-olean-12-en-3-yl palmitate (*β*-Amirin palmitate)	*Microtropis triflora* (stalks)[84]
**O40**	6*β*,29-Dihydroxy-olean-18-en-3-one	*Cassine xylocarpa* (stems)[191]
**O41**	6*β*-Hydroxy-olean-18-en-3-one	*Cassine xylocarpa* (stems)[191]
**O42**	7*β*,28-Dihydroxy-olean-12-en-3-one	*Euonymus carnosus* (stems)[154]
**O43**	11*α*-Hydroxy-3-oxo-olean-12-en-29-oic acid	*Maytenus undata* (leaves)[284]
**O44**	11*α*-Methoxi-3-oxo-olean-12-en-30-oic acid	*Maytenus undata* (leaves)[284]
**O45**	2,22*β*-Dihydroxy-3-oxo-olean-1,12-dien-29-oic acid	*Tripterygium wilfordii* (roots)[61]
**O46**	22*α*-Hydroxy-3-oxo-olean-12-	*Maytenus macrocarpa* (bark)[204]

Tabela S1: Pentacyclic triterpenoids isolated from Celastraceae species (2001-2021).

	Name	Species (plant part) Reference
	en-29-oic acid	*Salacia chinensis* (stems)[39]
**O47**	22α-Hydroxy-3-oxo-olean-12-en-30-oic acid (Macrocarpoic acid B)	*Maytenus macrocarpa* (bark)[204]
**O48**	22β-Hydroxy-3-oxo-olean-12-en-29-oic acid	*Maytenus macrocarpa* (bark)[204]
**O49**	23-Hydroxy-3-oxo-olean-12-en-28-oic acid	*Celastrus orbiculatus* (stems)[166]<br>*Tripterygium hypoglaucum* (aereal parts)[313]
**O50**	28-Hydroxy-3-oxo-olean-12-en-29-oic acid	*Tripterygium wilfordii* (roots)[61]
**O51**	2α,3β-Dihydroxy-olean-12-en-28-oic acid	*Euonymus alatus* (branches)[157]<br>*Euonymus grandiflorus* (leaves, branches)[306]<br>*Microtropis fokienensis* (stems)[190]
**O52**	3,11α-Dihydroxy-olean-12-en-30 oic acid	*Maytenus undata* (leaves)[284]
**O53**	2,3-*Seco*-22α,29-lactone-3-methyloxicarbonyl-olean-12-ene-2-oic acid	*Tripterygium wilfordii* (roots)[61]
**O54**	3-Oxo-24-*nor*-olean-12-en-28-oic acid	*Celastrus orbiculatus* (stems)[166]
**O55**	3-Oxo-olean-12-en-28-oic acid	*Celastrus orbiculatus* (stems)[166]
**O56**	3-Oxo-olean-12-en-30-oic acid (Catononic acid)	*Austroplenckia populnea* (stems)[315]
**O57**	3α,22β-Dihydroxy-olean-12-en-29-oic acid	*Euonymus grandiflorus* (leaves, branches)[306]<br>*Maytenus royleanus* (roots)[209]<br>*Salacia nitida* (leaves)[263]
**O58**	3α,28-Dihydroxy-olean-12-en-29-oic acid	*Tripterygium hypoglaucum* (aereal parts)[313]<br>*Tripterygium wilfordii* (roots)[61]
**O59**	3α,29-Dihydroxy-olean-12-en-28-oic acid	*Tripterygium hypoglaucum* (aereal parts)[313]
**O60**	3α,30-Dihydroxy-olean-12-en-28-oic acid	*Maytenus diversifolia* (aereal parts)[185]
**O61**	3α-Hydroxy-olean-12-en-23-oic acid (α-boswellic acid)	*Tripterygium hypoglaucum* (aereal parts)[313]
**O62**	3β,16α-Dihydroxy-23-formyl-olean-12-en-28-oic acid	*Euonymus tingen* (stem barks)[208]
**O63**	3β,21β-Dihydroxy-olean-12-en-29-oic acid (Ficusonic acid)	*Maytenus royleanus* (roots)[209]
**O64**	3β,22α-Dihydroxy-olean-12-en-29-oic acid (Maytenfolic acid)	*Austroplenckia populnea* (roots)[276]<br>*Maytenus heterophylla* (stem barks)[82]<br>*Maytenus macrocarpa* (barks)[204]<br>*Salacia chinensis* (stems)[39]
**O65**	3β,22α-Dihydroxy-olean-12-en-30-oic acid (Macrocarpoic acid A)	*Maytenus macrocarpa* (barks)[204]
**O66**	3β,22β-Dihydroxy-olean-12-en-	*Salacia chinensis* (stems)[39]

Tabela S1: Pentacyclic triterpenoids isolated from Celastraceae species (2001-2021).

	Name	Species (plant part) Reference
	29-oic acid (Maytenfolic acid-22*β*-*epi*)	*Salacia nitida* (lianas)[263]
**O67**	3*β*,23-Dihydroxy-olean-12-en-28-oic acid (Hederagenin)	*Celastrus orbiculatus* (stems)[314]<br>*Euonymus alatus* (branches)[157,169]<br>*Tripterygium hypoglaucum* (aereal parts)[313]
**O68**	3*β*,28-Dihydroxy-olean-12-en-29-oic acid	*Tripterygium wilfordii* (roots)[61]
**O69**	3*β*,6*β*,29-Trihydroxy-olean-12-en-28-oic acid (Gymnosporin B)	*Gymnosporia royleana* (aereal parts)[316]
**O70**	3*β*-Acetyloxy-olean-12-en-28-oic acid	*Celastrus orbiculatus* (stems)[314]<br>*Siphonodon celastrineus* (fruits)[317]
**O71**	3*β*-Hydroxy-1-oxo-olean-12-en-28-oic acid	*Euonymus alatus* (branches)[157]
**O72**	3*β*-Hydroxy-olean-11,13(18)-dien-28-oic acid	*Euonymus grandiflorus* (leaves, branches)[306]
**O73**	3*β*-Hydroxy-olean-12-en-28-oic acid (Oleanolic acid)	*Celastrus orbiculatus* (stems)[166]<br>*Crossopetalum uragoga* (stems)[126]<br>*Euonymus alatus* (root barks)[269]<br>*Maytenus boaria* (leaves)[310]<br>*Maytenus magellanica* (root barks)[126]<br>*Maytenus phyllanthoides* (leaves)[311]<br>*Microtropis triflora* (stalks)[84,270]<br>*Salacia chinensis* (leaves)[258]<br>*Tripterygium hypoglaucum* (aereal parts)[313]
**O74**	3*β*-Hydroxy-olean-12-en-29-oic acid (Catonic acid 3-*epi*)	*Austroplenckia populnea* (stems, roots)[275]<br>*Tripterygium doianumi* (branches)[233]
**O75**	11*α*,30-Dihydroxy-2,3-seco-olean-12-en-2,3-dicarboxylic anhydride	*Microtropis fokienensis* (stems)[190]
**O76**	3*β*-olean-12-en-3-yl ferulate (*β*-Amirin ferulate)	*Hippocratea excelsa* (root barks)[213]
**O77**	2,3-*Seco*-1*α*,3*α*-Oxido-olean-13(18)-en-1,3-olide (Gymnosporin A)	*Gymnosporia royleana* (aereal parts)[316]
**O78**	Olean-11,13(18)-dien-3*β*,30-diol	*Microtropis fokienensis* (stems)[190]
**O79**	Olean-12-en-3,11-dione	*Crossopetalum uragog*a (stems)[126]<br>*Maytenus magellanica* (root barks)[126]<br>*Maytenus cuzcoina* (root barks)[68]
**O80**	Olean-12-en-3-one (*β*-Amirone)	*Crossopetalum uragoga* (stems)[126]<br>*Maytenus magellanica* (root barks)[126]<br>*Salacia chinensis* (stems)[39]
**O81**	Olean-12-en-3*α*,11*α*,21*β*-triol	*Hippocratea excelsa* (stem bark)[187]
**O82**	Olean-12-en-3*α*,21*β*-diol	*Hippocratea excelsa* (stem bark)[187]
**O83**	Olean-12-en-3*α*,23,28-triol	*Maytenus diversifolia* (aereal parts)[185]

Tabela S1: Pentacyclic triterpenoids isolated from Celastraceae species (2001-2021).

	Name	Species (plant part) Reference
**O84**	Olean-12-en-3β,11α-diol	*Elaeodendron croceum* (leaves)[287]
**O85**	Olean-12-en-3β,22β-diol (Sophoridiol)	*Euonymus grandiflorus* (leaves, branches)[306]
**O86**	Olean-12-en-3β,28-diol (Erythrodiol)	*Salacia chinensis* (leaves)[258]
**O87**	Olean-12-en-3β,29-diol (Paniculatadiol)	*Maytenus apurimacensis* (roots)[160]
**O88**	Olean-12-en-3β,30-diol	*Microtropis fokienensis* (stems)[190]
**O89**	3β-olean-12-en-3-yl acetate (β-amirin acetate)	*Elaeodendron buchananii* (stem barks)[232]<br>*Glyptopetalum calocarpum* (leaves)[308]
**O90**	Olean-18-en-3,21-dione	*Cassine xylocarpa* (stems)[191]
**O91**	Olean-18-en-3-one	*Cassine xylocarpa* (stems) [191]
**O92**	Olean-18-en-3β,11α,28-triol	*Maytenus jelskii* (leaves)[191]
**O93**	Olean-18-en-3β,11α-diol (Nivadiol)	*Maytenus jelskii* (leaves)[191]
**O94**	Olean-18-en-3β,21α-diol	*Cassine xylocarpa* (stems)[191]
**O95**	Olean-18-en-3β,29-diol	*Cassine xylocarpa* (stems)[191]
**O96**	Olean-18-en-3β,6β-diol	*Cassine xylocarpa* (stems)[191]
**O97**	Olean-18-en-3β-ol	*Cassine xylocarpa* (stems)[191]
**O98**	Olean-9(11),12-dien-3-one	*Celastrus hypoleucus* (aereal parts)[198]<br>*Crossopetalum uragoga* (stems)[126]<br>*Maytenus cuzcoina* (root barks)[68]<br>*Maytenus magellanica* (root barks)[126]<br>*Maytenus distichophylla* (roots)[280]
**O99**	Olean-9(11),12-dien-3α,21β-diol	*Hippocratea excelsa* (stem barks)[187]
**O100**	Olean-9(11),12-dien-3β-ol	*Celastrus angulatus* (root barks)[318]<br>*Celastrus hypoleucus* (aereal parts)[198]<br>*Euonymus alatus* (root barks)[269]<br>*Maytenus cuzcoina* (root barks)[68]<br>*Salacia crassifoli*a (leaves)[6]
**O101**	11β,26-oxido-26-hydroxy-olean-12-en-3-one (Salacetal)	*Salacia longipes* (roots)[221]
**O102**	Olean-12-en-3β-ol, 3-[(2*E*)-3-[(2*S*,3*S*)-2-[(acetyloxy)methyl]-2,3-dihydro-3-(4-hydroxy-3-methoxyphenyl)-1,4-benzodioxin-6-yl]-2-propenoate] (Uragogin)	*Crossopetalum uragoga* (stems)[126]
	**URSANES**	
**U1**	11α,12,15α,20β-Tetrahydroxy-urs-12-en-3-one	*Siphonodon celastrineus* (stems)[193]
**U2**	11α,12,15α,24-Tetrahydroxy-urs-12-en-3-one	*Siphonodon celastrineus* (stems)[193]
**U3**	11α,12,16β-Trihydroxy-urs-12-en-3-one	*Microtropis fokienensis* (stems)[190]

Tabela S1: Pentacyclic triterpenoids isolated from Celastraceae species (2001-2021).

	Name	Species (plant part) Reference
**U4**	11*α*,12-[2-(Hydroxymethyl)-3-(4-hydroxy-3-methoxyphenyl)ethane-1,2-dioxy]-urs-12-ene-3*β*,15*α*-diol	*Siphonodon celastrineus* (stems)[54]
**U5**	11*α*,12-[3-(Hydroxymethyl)-2-(4-hydroxy-3-methoxyphenyl)ethane-1,2-dioxy]-urs-12-ene-3*β*,15*α* -diol	*Siphonodon celastrineus* (stems)[54]
**U6**	11*α*-Hydroxy-urs-12-en-3-one	*Maytenus cuzcoina* (root barks)[68]
**U7**	11*α*-Methoxy-12,15*α*,24-trihydroxy-urs-12-en-3-one	*Siphonodon celastrineus* (stems)[193]
**U8**	11*α*-Methoxy-3*β*,12,15*α*,20*β*-tetrahydroxy-urs-12-en-24-al	*Siphonodon celastrineus* (stems)[193]
**U9**	11*α*-Methoxy-3*β*,12,15*α*-trihydroxy-urs-12-en-24-al	*Siphonodon celastrineus* (stems)[193]
**U10**	11*α*-Methoxy-urs-12-en-2*α*,3*β*,12,24-tetrol	*Siphonodon celastrineus* (stems)[193]
**U11**	11*α*-Methoxy-urs-12-en-3-one	*Maytenus spinosa* (roots)[104]
**U12**	11*α*-Methoxy-urs-12-en-3*β*,12,15*α*,20*β*-tetrol	*Siphonodon celastrineus* (stems)[193]
**U13**	11*α*-Methoxy-urs-12-en-3*β*,12,15*α*,24-tetrol	*Siphonodon celastrineus* (stems)[193]
**U14**	11*α*-Methoxy-urs-12-en-3*β*,12,15*α*-triol	*Siphonodon celastrineus* (stems)[54]
**U15**	11*α*-Methoxy-urs-12-en-3*β*,12,24-triol	*Siphonodon celastrineus* (stems)[193]
**U16**	11*α*-Methoxy-urs-12-en-3*β*,12-diol	*Siphonodon celastrineus* (stems)[54]
**U17**	11*α*-Methoxy-urs-12-en-3*β*,15*α*-diol	*Siphonodon celastrineus* (stems)[54]
**U18**	11*α*-Methoxy-urs-12-en-3*β*,28-diol	*Microtropis fokienensis* (leaves)[189]
**U19**	11*α*-Methoxy-urs-12-en-3*β*-ol	*Microtropis fokienensis* (leaves)[189]
**U20**	11*β*,16*β*-Dihydroxy-urs-12-en-3-one	*Hippocratea excelsa* (stem barks)[187]
**U21**	11*β*-Hydroxy-urs-12-en-3-one	*Hippocratea excelsa* (stem barks)[187]
**U22**	12,15*α*,24-Trihydroxy-3,11-dioxo-urs-12-en-30-oic acid methyl ester	*Siphonodon celastrineus* (stems)[193]
**U23**	12,15*α*,20*β*-Trihydroxy-urs-12-en-3,11-dione	*Siphonodon celastrineus* (stems)[54]
**U24**	12,15*α*,24,30-Tetrahydroxy-urs-12-en-3,11-dione	*Siphonodon celastrineus* (stems)[193]
**U25**	12,15*α*,24-Trihydroxy-urs-12-en-3,11-dione	*Siphonodon celastrineus* (stems)[54]
**U26**	3*β*,12,16*β*,23-Tetrahydroxy-11*α*-methoxyurs-12-ene 3,16-Di-O-*β*-D-glucopyranoside	*Microtropis japonica* (leaves)[230]
**U27**	13*β*,28-Oxido-urs-11-en-3*β*,15*α*-diol	*Siphonodon celastrineus* (stems)[54]

Tabela S1: Pentacyclic triterpenoids isolated from Celastraceae species (2001-2021).

	Name	Species (plant part) Reference
**U28**	13*β*,28-Oxido-urs-11-en-3*β*,16*β*-diol	*Microtropis fokienensis* (leaves, stems)[189,190]
**U29**	13*β*,28-Oxido-urs-11-en-3*β*,23-diol	*Microtropis fokienensis* (leaves)[189]
**U30**	13*β*,28-Oxido-urs-11-en-3*β*-ol	*Microtropis fokienensis* (leaves, stems)[153,189]
**U31**	15*α*,20*β*-Dihydroxy-13*β*,28-oxido-urs-11-en-3-one	*Siphonodon celastrineus* (stems)[193]
**U32**	15*α*-Benzoyloxy-3*β*,11*α*,12-trihydroxy-urs-12-en-24-al	*Siphonodon celastrineus* (stems)[54]
**U33**	15*α*-Benzoyloxy-urs-12-en-3*β*,11*α*-diol	*Siphonodon celastrineus* (stems)[54]
**U34**	28,30-Dihydroxy-urs-12-en-3-one	*Maytenus diversifolia* (aereal parts)[185]
**U35**	2*α*,3*β*,12,24-Tetrahydroxy-urs-12-en-11-one	*Siphonodon celastrineus* (stems)[193]
**U36**	2*α*,3*β*,24-trihydroxyurs-12-en-28-oic acid 28-O-*β*-D-glucopyranosyl-(1→2)-*β*-D-glucopyranoside	*Salacia cochinchinensis* (leaves, branches)[194]
**U37**	2*α*,3*β*,19*α*,24-Tetrahydroxy-urs-12-en-28-oic acid 28-O-*β*-D-glucopyranosyl-(1→2)-*β*-D-glucopyranoside	*Salacia cochinchinensis* (leaves, branches)[194]
**U38**	3*β*-(*E*)-Caffeoyloxy-urs-12-en-28-ol (3-(*E*)-caffeoyluvaol)	*Maytenus macrocarpa* (branches)[204]
**U39**	3*β*-(*E*)-Coumaroyloxy-urs-12-en-28-ol [3-(*E*)-coumaroyluvaol; Macrocarpol A]	*Maytenus macrocarpa* (branches)[204]
**U40**	30-Hydroxy-urs-12-en-3,11-dione	*Maytenus diversifolia* (aereal parts)[185]
**U41**	3*α*,30-Dihydroxy-urs-12-en-11-one	*Maytenus diversifolia* (aereal parts)[185]
**U42**	3*β*,11*α*,12,15*α*,20*β*-Pentrahydroxy-urs-12-en-24-al	*Siphonodon celastrineus* (stems)[193]
**U43**	3*β*,11*α*,12,15*α*-Tetrahydroxy-urs-12-en-24-al	*Siphonodon celastrineus* (stems)[54]
**U44**	3*β*,11*α*,12,15*α*-Tetrahydroxy-urs-12-en-24-oic acid methyl ester	*Siphonodon celastrineus* (stems)[54]
**U45**	3*β*,11*α*,15*α*,20*β*-Tetrahydroxy-13*α*,27-cyclours-12-one	*Siphonodon celastrineus* (stems)[193]
**U46**	3*β*,11*α*,15*α*-Trihydroxy-12-oxo-13*α*,27-cyclours-24-al	*Siphonodon celastrineus* (stems)[193]
**U47**	3*β*,11*α*,15*α*-trihydroxyi-13*α*,27-cyclours-12-one	*Siphonodon celastrineus* (stems)[193]
**U48**	3*β*,12,15*α*,24-Tetrahydroxy-urs-12-en-11-one	*Siphonodon celastrineus* (stems)[193]
**U49**	3*β*,12,15*α*-Trihydroxy-urs-12-en-11-one	*Siphonodon celastrineus* (stems)[54]
**U50**	3*β*,12,24-Trihydroxy-urs-12-en-11-one	*Siphonodon celastrineus* (stems)[54]
**U51**	3*β*,16*β*-Dihydroxy-urs-12-en-11-one	*Microtropis fokienensis* (stems)[190]

Tabela S1: Pentacyclic triterpenoids isolated from Celastraceae species (2001-2021).

	Name	Species (plant part) Reference
U52	3β,23-Dihydroxy-13β,28-oxido-urs-12-one (Microfokienoxane C)	*Microtropis fokienensis* (leaves)[189]
U53	3β,25-Oxido-urs-12-en-1α,3α,11α,12,23,25-hexaol	*Microtropis triflora* (stalks)[84]
U54	3β,30-Dihydroxy-urs-12-en-11-one	*Maytenus diversifolia* (aereal parts)[185]
U55	3β-Acetyloxy-11α,19α,28-trihydroxy-urs-12-en-23-oic acid methyl ester	*Elaeodendron buchananii* (stem barks)[232]
U56	3β-Acetyloxy-urs-11-en-13α,30-olide	*Tripterygium doianumi* (branches)[233]
U57	3β-Acetyloxy-urs-12-en-19α,23,28-triol	*Elaeodendron buchananii* (stem barks)[232]
U58	3β-Acetyloxy-urs-12-ene	*Salacia nitida* (leaves)[263]
U59	3β-Stearyloxy-urs-12-ene<br>(α-Amyrin stearate)	*Cheiloclinium cognatum* (leaves)[69]<br>*Maytenus distichophylla* (leaves)[265]<br>*Maytenus salicifolia* (leaves)[161,235]<br>*Salacia crassifolia* (leaves)[265]<br>*Salacia elliptica* (leaves)[43]
U60	3β-Hydroxy-urs-11-en-13β,28-olide	*Maytenus phyllanthoides* (leaves)[311]
U61	3β-Hydroxy-urs-12-en-11-one	*Maytenus cuzcoina* (root barks)[68]
U62	3β-Palmitoyloxy-urs-12-ene<br>(α-Amyrin palmitate)	*Cheiloclinium cognatum* (leaves)[69]<br>*Maytenus distichophylla* (leaves)[265]<br>*Salacia crassifolia* (leaves)[6]
U63	6β,12,23-Trihydroxy-11α-methoxy-urs-12-en-3-one	*Microtropis fokienensis* (stems)[190]
U64	19α,28-Dihydroxy-3-oxo-urs-12-en-24-oic acid	*Elaeodendron buchananii* (stem barks)[232]
U65	21β-Hydroxy-3-oxo-2,3-*seco*-urs-12-en-2-oic acid	*Siphonodon celastrineus* (stems)[54]
U66	22α-Hydroxy-3-oxo-urs-12-en-30-oic acid (Demethylregelin)	*Salacia chinensis* (stems)[39]
U67	22β-Hydroxy-3-oxo-urs-12-en-30-oic acid	*Tripterygium wilfordii* (roots)[61]
U68	28-Hydroxy-3-oxo-urs-12-en-30-oic acid	*Tripterygium wilfordii* (roots)[61]
U69	2α,3β-Dihydroxy-urs-12-en-28-oic acid	*Euonymus alatus* (branches)[157]<br>*Microtropis fokienensis* (stems)[190]
U70	2α-Hydroxy-3-oxo-urs-12-en-28-oic acid	*Tripterygium doianum* (branches)[233]
U71	3,11-Dioxo-urs-12-en-28-oic acid	*Maytenus diversifolia* (aereal parts)[185]
U72	3α-Hydroxy-11-oxo-urs-12-en-28-oic acid	*Maytenus diversifolia* (aereal parts)[185]
U73	3β,11α,19α-Trihydroxy-urs-12-en-23,28-dioic acid	*Elaeodendron buchananii* (stem barks)[232]

Tabela S1: Pentacyclic triterpenoids isolated from Celastraceae species (2001-2021).

	Name	Species (plant part) Reference
U74	3*β*,22*α*-Dihydroxy-urs-12-en-30-oic acid (Tripterygic acid A)	*Salacia chinensis* (stems)[39]
U75	3*β*,7*β*,24-Trihydroxy-urs-12-en-28-oic acid (Gymnosporin C)	*Gymnosporia royleana* (aereal parts)[316]
U76	3*β*-Acetylxy-urs-12-en-28-oic acid	*Microtropis fokienensis* (stems)[190]
U77	3*β*-Hydroxy-urs-12-en-28-oic acid (Ácido ursólico)	*Elaeodendron buchananii* (stem barks)[232]<br>*Euonymus alatus* (branches)[157]<br>*Euonymus grandiflorus* (leaves, branches)[306]<br>*Salacia chinensis* (leaves)[258]<br>*Siphonodon celastrineus* (fruits)[317]
U78	2*α*,3*β*,19*α*-Trihydroxy-urs-12-en-28-oic acid 28-O-*β*-D-glucopyranosyl-(1→2)-*β*-D-glucopyranoside (Officinoterpenoside B)	*Salacia cochinchinensis* (leaves, branches)[194]
U79	Urs-12-en-3,11-dione	*Maytenus cuzcoina* (root barks)[68]
U80	Urs-12-en-3*β*,11*α*,12,15*α*-tetrol	*Siphonodon celastrineus* (stems)[54]
U81	Urs-12-en-3*β*,11*α*,12,24-tetrol	*Siphonodon celastrineus* (stems)[193]
U82	Urs-12-en-3*β*,11*α*,15*α*-triol	*Siphonodon celastrineus* (stems)[54]
U83	Urs-12-en-3*β*,25,30-triol	*Salacia crassifolia* (roots)[243]
U84	Urs-12-en-3*β*,28-diol	*Euonymus alatus* (root barks)[269]<br>*Microtropis fokienensis* (leaves)[189]<br>*Salacia chinensis* (leaves)[258]
U85	Urs-12-en-3*β*-ol (*α*-amirin)	*Cheiloclinium cognatum* (branches)[268]<br>*Hippocratea excelsa* (stem barks)[187]<br>*Maytenus distichophylla* (leaves)[265]<br>*Maytenus phyllanthoides* (leaves)[311]<br>*Maytenus salicifolia* (leaves)[235]<br>*Salacia amplifolia* (roots)[259]<br>*Salacia crassifolia* (leaves)[265]<br>*Salacia hainanensis* (roots)[73]<br>*Salacia impressifolia* (stems, branches)[285]
U86	Urs-14-en-3*β*-ol (Isoursenol)	*Salacia chinensis* (leaves)[258]
U87	Urs-9(11),12-dien-3-one	*Maytenus cuzcoina* (root barks)[68]
U88	Urs-9(11),12-dien-3*β*-ol	*Maytenus cuzcoina* (root barks)[68]
	**OTHERS**	
OT1	Gammacer-12-en-3*β*-ol (12-gammateraen-3*β*-ol; tetrehymanol; Monechmol)	*Celastrus hypoleucus* (aereal parts)[198]
OT2	18α(*H*)-taraxastane-3*β*,20*α*-diol	*Salacia chinensis* (leaves)[258]
OT3	21*β*-*H*-hop-22(29)-en-3-one	*Maytenus robusta* (leaves)[94]
OT4	21*β*-*H*-hop-22(29)-en-3*β*-ol	*Maytenus robusta* (leaves)[94]

Tabela S1: Pentacyclic triterpenoids isolated from Celastraceae species (2001-2021).

	Name	Species (plant part) Reference
**OT5**	25(9→8),26(13→14)Abeo-24-nor-8,14-*seco*-friedelan-2,3-dihydroxy-1,3,5(10),6,8-pentaen-29(13)-olide	*Celastrus orbiculatus* (root barks)[139]
**OT6**	Glutin-5-en-3*β*,29-diol (29-Hydroxy-glutinol)	*Cassine xylocarpa* (root barks)[196]<br>*Celastrus vulcanicola* (root barks)[196]<br>*Elaeodendron schlechteranum* (root barks)[63]<br>*Hippocratea excelsa* (root barks)[213]<br>*Maytenus apurimacensis* (roots)[160]<br>*Maytenus salicifolia* (fruit pulp)[283]
**OT7**	14-Taraxen-3*β*,29-diol (29-Hydroxy-taraxerol)	*Hippocratea excelsa* (root barks)[213]
**OT8**	24,25,26-Trinoroleana-4,7-dien-6*α*,10*α*-dihydroxy-9*β*,13*α*-dimethyl-2,3-dioxo-29-oic acid, methyl ester (6-*epi*-barudiona)	*Cheiloclinium hippocratioides* (root barks)[138]
**OT9**	25(9→8)Abeo-24-nor-8,14-*seco*-friedelan-2,3-dihydroxy-1,3,5(10),6,8,14(27)-hexaen-29-oic acid	*Celastrus orbiculatus* (root barks)[139]
**OT10**	25(9→8)Abeo-24-nor-friedelan-2,3-dihydroxy-1,3,5(10),6,8,11-hexaen-29-oic acid	*Celastrus orbiculatus* (root barks)[139]
**OT11**	25(9→8)abeo-24-norfriedelan-2,3-dioxo-1(10),4,6,9(11)-tetraen-29-oic acid	*Celastrus orbiculatus* (root barks)[139]
**OT12**	(21*β*)-13,17-Dimethyl-A'-neo-26,28-dinorgammacer-9(11)-en-3-one (Arborinone)	*Euonymus alatus* (cork)[305]
**OT13**	Campestrine I	*Peritassa campestris* (roots)[252]
**OT14**	Campestrine II	*Peritassa campestris* (roots)[252]
**OT15**	Cassinolide	*Cassine xylocarpa* (root barks)[319]
**OT16**	Glutin-5-en-3*α*,29-diol	*Salacia staudtiana* (root barks)[278]
**OT17**	Olean-18-en-3*β*-ol (Germanicol)	*Cassine xylocarpa* (stems)[191]<br>*Euonymus alatu*s (cork)[305]
**OT18**	Glutin-5-en-3*β*,21*α*-diol	*Cassine xylocarpa* (root barks)[196]<br>*Celastrus vulcanicola* (root barks)[196]
**OT19**	Glutin-5-en-3*β*-ol (Glutinol)	*Cassine xylocarpa* (root barks)[196]<br>*Celastrus vulcanicola* (root barks)[196]<br>*Cheiloclinium cognatum* (branches)[268]<br>*Euonymus alatus* (branches)[157]<br>*Maytenus cuzcoina* (root barks)[68]<br>*Maytenus salicifolia* (root barks)[105]

Tabela S1: Pentacyclic triterpenoids isolated from Celastraceae species (2001-2021).

	Name	Species (plant part) Reference
		Salacia amplifolia (roots)[259]
**OT20**	(21*β*)-13,17-Dimethyl-A'-neo-26,28-dinorgammacer-9(11)-en-3*β*-ol (Isoarborinol)	*Euonymus fortunei* (aerial parts)[320]
**OT21**	14-Taraxen-3*β*–ol (Taraxerol)	*Euonymus alatus* (cork)[305]
		Maytenus undata (leaves)[284]
